# Comparative Efficacy of East Asian Herbal Formulae Containing Astragali Radix–Cinnamomi Ramulus Herb-Pair against Diabetic Peripheral Neuropathy and Mechanism Prediction: A Bayesian Network Meta-Analysis Integrated with Network Pharmacology

**DOI:** 10.3390/pharmaceutics15051361

**Published:** 2023-04-28

**Authors:** Hee-Geun Jo, Eunhye Baek, Donghun Lee

**Affiliations:** 1 Department of Herbal Pharmacology, College of Korean Medicine, Gachon University, 1342 Seongnamdae-ro, Sujeong-gu, Seongnam 13120, Republic of Korea; 2 Naturalis Inc., 6 Daewangpangyo-ro, Bundang-gu, Seongnam 13549, Republic of Korea; 3 RexSoft Inc., 1 Gwanak-ro, Gwanak-gu, Seoul 08826, Republic of Korea

**Keywords:** herbal medicine, diabetic peripheral neuropathy, network meta-analysis, network pharmacology, herb-pair

## Abstract

The Astragali Radix–Cinnamomi Ramulus herb-pair (ACP) has been widely used in the treatment of diabetic peripheral neuropathy (DPN) as part of East Asian herbal medicine (EAHM). Eligible randomized controlled trials (RCTs) were identified by searching 10 databases. The outcomes investigated were response rate, sensory nerve conduction velocity (SNCV), and motor nerve conduction velocity (MNCV) in four regions of the body. The compounds in the ACP and their targets of action, disease targets, common targets, and other relevant information were filtered using network pharmacology. Forty-eight RCTs, with 4308 participants, and 16 different interventions were identified. Significant differences were observed in the response rate, MNCV, and SNCV, as all EAHM interventions were superior to conventional medicine or lifestyle modification. The EAHM formula containing the ACP ranked highest in more than half of the assessed outcomes. Furthermore, major compounds, such as quercetin, kaempferol, isorhamnetin, formononetin, and beta-sitosterol, were found to suppress the symptoms of DPN. The results of this study suggest that EAHM may increase therapeutic efficacy in DPN management, and EAHM formulations containing the ACP may be more suitable for improving treatment response rates to NCV and DPN therapy.

## 1. Introduction

Diabetic peripheral neuropathy (DPN) is a common complication, affecting around half of diabetic patients [1,2]. Both diabetes and prediabetes can cause DPN, leading to various types of nerve damage and accompanying clinical findings [3,4,5]. Accordingly, approximately 40% of patients with DPN develop neuropathic pain that does not respond to treatment, and various motor dysfunctions and sensory losses [4,6]. Owing to these pathophysiological characteristics, DPN not only reduces the quality of life of patients but also imposes an immense social burden. A recent study reinforced this problem by reporting that the medical expenses of patients with painful DPN are 20% higher than those of diabetic patients without corresponding complications, and the cost increases over time [7]. Various interventions for the treatment and management of DPN have been discussed; however, no disease-modifying treatment is available [1,8]. Currently, the main therapies focus on symptomatic pain relief using conventional medicines (CM), such as anticonvulsants, antidepressants, methylcobalamin, and alpha-lipoic acid [9,10]. Therefore, further investigation is needed to develop candidate drugs that can alleviate DPN-related systemic pathophysiology while exhibiting fewer adverse events.

Natural products are considered a promising alternative treatment because they can be applied to the multifaceted lesions of DPN owing to their safety and higher patient compliance [11,12,13,14,15]. Recent studies have reported that widely used plant compounds can improve DPN because of their neuroprotective, antioxidative, and anti-neuroinflammatory effects [16]. Flavonoids, alkaloids, phenolic compounds, terpenoids, saponins, and phytosterol-type constituents of herbal medicines used worldwide are being actively studied as new drug candidates for the treatment of various diabetic complications [17]. Among these, East Asian herbal medicine (EAHM) is an area of natural medicine in which therapeutic candidates for DPN have been the most actively investigated [10,12,18,19,20,21]. EAHM is a generic term for natural materials used as medicines for the treatment of diseases in many countries in East Asia, including Korea, China, Taiwan, and Japan, and the study thereof [22,23,24,25,26]. EAHM is operated under a unique prescription principle that seeks to maximize the synergistic effect of polyherbal formulae and is distinct from herbal medicine in other regions of the world in that treatments using the same materials are practiced in several countries [27,28,29,30]. EAHM is still being actively used for treatment in the aforementioned regions, and substantial clinical and preclinical evidence establish its efficacy [19,31,32,33]. Therefore, considering the efficacy of EAHM in DPN treatment, the EAHM constituents conducive to DPN mitigation must be investigated.

As described above, in EAHM, the synergistic effect of drug combinations is as important as that of a single material. A combination of two or three materials capable of obtaining such a synergistic effect is called an “herb-pair”, which is the smallest unit of an EAHM polyherbal formula and an appropriate unit of analysis for investigating candidate therapeutics [34,35,36,37]. One useful herb-pair for DPN, as suggested in several previous studies and by traditional experience, is the Astragali Radix–Cinnamomi Ramulus herb-pair (ACP). Previous reviews have reported the effectiveness of prescriptions, including ACP, for cervical radiculopathy, neuropathy with DPN, and other mechanisms [38,39]. Furthermore, previous studies assessing the effect of EAHM on peripheral neuropathy and association rule mining on individual materials have revealed a pattern of relationships between the two herbs constituting the ACP [40]. In addition, preclinical evidence related to Hwanggi Gyeji Omul-tang (Huangqi Guizhi Wuwu decoction in Chinese), a representative EAHM formula containing the ACP, shows that this combination may exert neuroprotective effects against various causes of neuropathy and nerve damage [41,42,43,44]. Several approaches are being used to explore effective EAHM formulae or herb-pairs that are believed to have synergistic effects. However, a research method for comparing the benefits of the anticipated synergistic effects of individual herb-pairs, rather than multiherbal formula units, has not yet been fully established.

Accordingly, we hypothesized that the ACP might be a combination that can show high efficacy in the treatment of DPN. To verify this hypothesis, this study was conducted as follows: (1) After a systematic search for randomized controlled trials in patients with DPN, a Bayesian network meta-analysis was performed to determine whether the EAHM formula containing the ACP was superior to that without the ACP. (2) Further network pharmacology analyses of the ACP were performed to predict the compounds and gene targets involved in the putative synergistic mechanisms. Therefore, we aimed to explore the efficacy of ACP in the treatment of DPN and its potential as a candidate drug.

## 2. Materials and Methods

This study was conducted in accordance with the Preferred Reporting Items for Systematic Reviews and Meta-Analyses extension statement for network meta-analysis [45]. The protocol for this systematic review was registered in PROSPERO (registration number: CRD42021290004). This study was conducted as a process of building multidisciplinary-integrative-decision making-actual achievement-scientific creativity (M.I.D.A.S) research platform.

### 2.1. Search Strategy

A comprehensive electronic search of four databases in English (PubMed, Cochrane Library, Cumulative Index to Nursing and Allied Health Literature (CINAHL), and EMBASE, four Korean databases (Korean Studies Information Service System (KISS), Research Information Service System (RISS), Oriental Medicine Advanced Searching Integrated System (OASIS), and Korea Citation Index (KCI)), one Chinese database (Chinese National Knowledge Infrastructure Database (CNKI)), and one Japanese database (CiNii) was conducted by two investigators from inception to 20 July 2021. The following Boolean format was used for the search: (mononeuropathy (MeSH) OR nerve compression syndromes (MeSH) OR neuralgia (MeSH) OR polyneuropathies (MeSH)) AND (“neuropathy”(Title/Abstract) OR “peripheral neuropathy”(Title/Abstract) OR “neuropathic pain”(Title/Abstract) OR “neuralgia”(Title/Abstract)) AND (“Medicine, Chinese Traditional”(MeSH) OR “Medicine, Kampo”(MeSH) OR “Medicine, Korean Traditional”(MeSH) OR “Herbal Medicine”(MeSH)). These search terms were appropriately modified to perform a search in the Korean, Chinese, and Japanese databases. The detailed search strategy is shown in Supplementary Table S1.

### 2.2. Study Selection

#### 2.2.1. Type of Studies

Only randomized controlled trials (RCTs) evaluating the efficacy and safety of oral administration of EAHM for DPN were included. There were no restrictions on language, publication date, or type of diabetes. Studies were excluded if they met any of the following criteria: (a) not an RCT or quasi-RCT; (b) a control group was not used or was inappropriate; (c) unrelated to manifestations due to DPN; (d) animal experiments; (e) case reports or reviews; or (f) not published in peer-reviewed scientific journals, including postgraduate theses or dissertations. 

#### 2.2.2. Type of Participants

Trials were considered eligible for inclusion if they were conducted on adults (age > 18 years) diagnosed with DPN with no restrictions on age, sex, or race.

#### 2.2.3. Type of Interventions

RCTs trials comparing EAHM as an active intervention in the treatment group with CM in the control group were included. However, RCTs that used a combination of EAHM and CM as an intervention were beyond the scope of this review and were omitted. All dosage forms of EAHM intervention for symptom management in DPN, such as decoctions, granules, and capsules, were included. Studies in which East Asian medical interventions such as acupuncture, massage, or other nondrug therapies were only combined in the treatment group were excluded. Studies in which the control group included other EAHM were excluded. Even if all other inclusion criteria were satisfied, RCTs in which the exact constituent herbs of the EAHM formulation used as an intervention were not identified were excluded.

#### 2.2.4. Type of Outcome Measures

The remission rate of DPN-related global symptoms observed according to the explicit criteria was selected as the outcome measure. However, most of the included studies reported the remission rates of complete remission (CR), partial remission (PR), mild remission (MR), and no remission (NR) as CR + PR/all patients. Considering that the remission rates reported by individual studies would have led to inconsistencies in the outcomes because different studies used different categorization criteria, the proportion of patients who achieved symptom alleviation in each group was used as the response rate in this review, and various study results were converted into this system.

The first set of secondary outcomes was indices evaluating motor nerve conduction velocities associated with neurological abnormalities in patients with DPN. Therefore, to evaluate the neurological improvement of the motor nerves, the median motor nerve conduction velocity (MMNCV), ulnar motor nerve conduction velocity (UMNCV), peroneal motor nerve conduction velocity (PMNCV), and tibial motor nerve conduction velocity (TMNCV) were selected as indices for each upper and lower extremity. The second set of secondary outcomes was indices evaluating sensory nerve conduction velocities associated with neurological abnormalities in patients with DPN. Accordingly, the median sensory nerve conduction velocity (MSNCV), ulnar sensory nerve conduction velocity (USNCV), peroneal sensory nerve conduction velocity (PSNCV), and tibial sensory nerve conduction velocity (TSNCV) were selected as indices for the upper and lower limbs. Finally, the adverse events occurring in each intervention and control group were used as safety evaluation indicators.

### 2.3. Data Extraction

According to the aforementioned search strategy, titles and abstracts of potentially eligible studies were independently screened by two investigators (H.-G.J. and D.L.). Subsequently, a full-text review was conducted based on the inclusion and exclusion criteria. Information from the included studies was independently extracted by two reviewers (H.-G.J. and D.L.). The following information was collected: title, author name, the country where the clinical trial was conducted, diagnostic criteria, trial design, publication year, sample size, participant age, sex distribution, interventions in the treatment group and comparators, treatment duration, outcome index, reported adverse events, EAHM composition, and dosage. Discrepancies were resolved through discussions between the two investigators.

### 2.4. Methodological Quality Assessment

Two investigators (H.-G.J. and D.L.) independently evaluated the methodological quality of each included study using the revised tool for the risk of bias in randomized trials, RoB 2 [46]. RoB 2 is characterized by the following five bias domains: bias arising from the randomization process, bias due to deviations from intended interventions, bias due to missing outcome data, bias in selecting the reported results, and bias in the measurement of the outcome. Methodological quality was assessed at three levels: “high risk of bias”, “low risk of bias”, and “some concerns”. Disagreements between the two investigators were resolved through discussions.

### 2.5. Data Analysis

#### 2.5.1. Pairwise Meta-Analysis

A pairwise meta-analysis (PMA) was performed to directly compare the EAHM with the comparator. Evidence synthesis of the included studies using the available data was performed by calculating the effect size and 95% confidence interval (CI) using a random-effects model. Heterogeneity was considered statistically significant when the *p*-value based on the χ^2^ test was <0.10 or I² was ≥50%. Two-sided *p* < 0.05 was considered statistically significant. Statistical synthesis of individual research results was performed using the software R (version 4.1.2; R Foundation for Statistical Computing, Vienna, Austria) and RStudio version 2022.02.3 build 492 (Integrated Development for R. RStudio, PBC, Boston, MA, USA) using the default settings of the ”meta” and ”metafor” package [47]. The RR and 95% confidence interval (CI) were calculated for the response rate. The mean difference (MD) and 95% confidence interval (CI) were calculated for the motor and sensory nerve conduction velocities. If heterogeneity was observed in the synthesized meta-analysis results for outcome measures involving > 10 trials, the cause of heterogeneity was traced using sensitivity analysis. To distinguish publication bias, a contour-enhanced funnel plot that included most of the studies was used [48]. To address the asymmetry of the visually confirmed funnel plot, Egger’s test [49] and Begg’s test [50] were performed to confirm publication bias. The overall quality of evidence for each outcome was evaluated using the Grading of Recommendations Assessment, Development, and Evaluation (GRADE) Pro [51]. The GRADE assessment evaluated the overall quality of evidence on four levels: very low, low, moderate, and high. The level of evidence is lowered by factors such as the risk of bias, inconsistency, indirectness, imprecision, and publication bias.

#### 2.5.2. Network Meta-Analysis

A network meta-analysis (NMA) was performed to evaluate the relative efficacy of EAHM formulae containing AC herb-pairs and other interventions. In this review, Bayesian NMAs were performed using R v. 4.1.2, and RStudio V. 2022.02.3 build 492 to evaluate the comparative effectiveness of treatments, using the most commonly used control intervention as a common comparator. The default settings of the R packages ”BUGSnet” and ”GeMTC” were used for the implementation of NMA [52,53]. For the response rate results, the effect was measured as an odds ratio (OR) with a 95% credible interval (Crl) using the binomial distribution assumption and logit link function. For the results of the eight nerve conduction velocity indices, the effects were analyzed as MD with 95% Crl using the normal likelihood model and identity link function. Markov chain Monte Carlo (MCMC) simulations were set up with a burn-in of 20,000 iterations and a total of 50,000 iterations, and every 10th value was extracted. Convergence was graphically assessed using trace and density plots. Node splitting was performed to assess the consistency of the response rate and a leverage plot was used to compare the DIC of the model based on the consistency assumption and the inconsistent model for the secondary outcome. A heat map with all feasible comparisons was constructed using the relative effect estimates from the NMA. We used a surface under the curve cumulative ranking probabilities (SUCRA) plot to demonstrate the ranking of treatments. 

#### 2.5.3. Network Pharmacology Analysis of the Synergistic Mechanism of the ACP against DPN

All bioactive ingredients in the ACP were screened and retrieved from the Traditional Chinese Medicine Systems Pharmacology (TCMSP; https://tcmsp-e.com/) analysis platform [54]. In this study, components with oral bioavailability (OB) ≥ 30% and drug-likeness (DL) index ≥ 0.18 were selected as candidate ingredients. The target information of active ingredients was standardized using the Uniprot database (http://www.uniprot.org/) with the species filter “*Homo sapiens*”. Using “diabetic peripheral neuropathy” as the keyword, data on DPN-related target genes were obtained from the GeneCards database (http://www.genecards.org). For targets in GeneCards, only those with a score ≥ 10 were screened [55]. Venn diagrams of consensus targets between the ACP and DPN were constructed using the Bioinformatics and Evolutionary Genomics website (https://bioinformatics.psb.ugent.be/webtools/Venn/). Using Cytoscape (v. 3.9.1; https://cytoscape.org/), a network of the components of the ACP and DPN targets was created to graphically depict the complex interactions between compounds and targets. The degree of each node is measured using a layout tool: the larger the node in the network, the higher the degree. The STRING protein analysis platform (v. 11.5; https://string-db.org/), together with the protein categorization “Homo sapiens”, was used to import the interacting gene targets of the ACP and DPN [56]. Protein interaction network analysis was performed and Cytoscape software version 3.9.1 [57] was used to construct protein–protein interaction (PPI) network maps. Gene targets with a degree of centrality above the average value were selected as hub targets. Gene ontology (GO) functional analysis was used as the primary method to describe the functions of gene targets, including biological processes, cellular components, and molecular functions. Kyoto Encyclopedia of Genes and Genomes (KEGG) enrichment analysis was used to identify common targets of the ACP and DPN in the signaling pathways. Metascape (https://metascape.org/), an online tool for gene enrichment analysis, incorporates more than 40 functional annotation datasets [58]. Hub targets were uploaded to the Metascape platform for GO and KEGG analyses. The data selection criterion was set at *p* < 0.05.

## 3. Results

### 3.1. Study Identification

**Figure 1:**
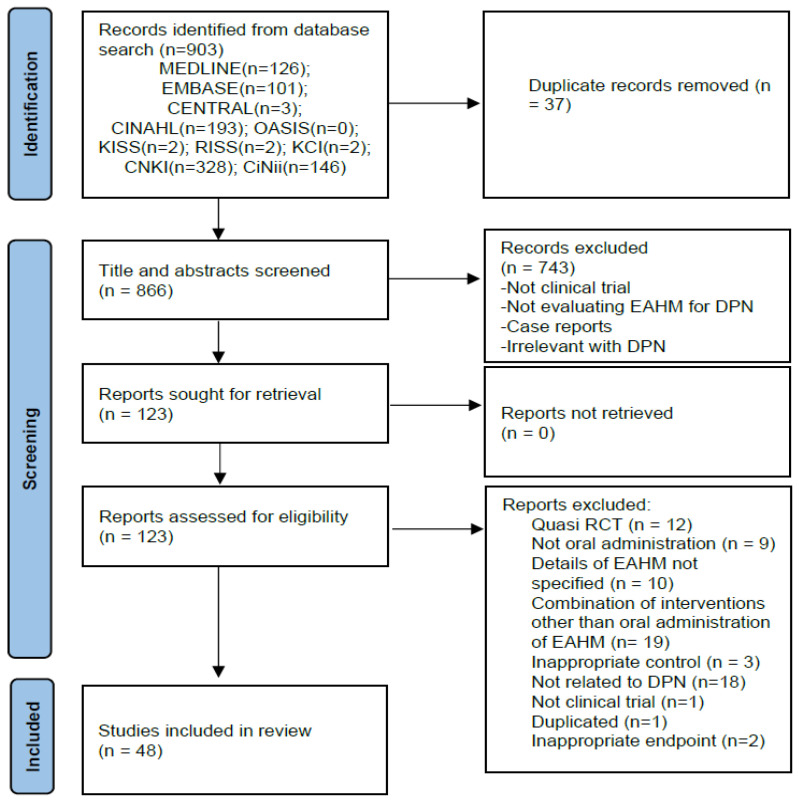
PRISMA 2020 flow diagram.

Based on the search strategy, 903 potentially relevant articles were identified through electronic searches of 10 databases. After excluding 37 duplicates, 866 articles were retrieved. After screening titles and abstracts, 743 articles that met at least one of the exclusion criteria were excluded. The full texts of the remaining 123 studies were assessed, and 75 articles were excluded for the reasons listed in Figure 1. Finally, 48 eligible studies were included in our meta-analysis [59,60,61,62,63,64,65,66,67,68,69,70,71,72,73,74,75,76,77,78,79,80,81,82,83,84,85,86,87,88,89,90,91,92,93,94,95,96,97,98,99,100,101,102,103,104,105,106]. The screening process is summarized in the PRISMA 2020 flow diagram (Figure 1).

### 3.2. Study Characteristics

The basic characteristics of the included studies are summarized in Table 1. The 48 trials included in this review were published between 2004 and 2021. A total of 4308 participants in the included studies were divided into experimental (n = 2175) and control groups (n = 2133), with sample sizes ranging from 29 to 202 participants. The average age of participants ranged from 38.7 to 69.8 years. The duration of neuropathy manifestations ranged from one month to >15 years. In 21 trials, the effects of EAHM monotherapy were compared with those of the comparator [59,61,64,65,70,75,76,79,81,82,83,86,87,88,90,93,95,99,100,101,102]. All studies that used EAHM monotherapy compared its effects with those of CM, except for one trial that adopted lifestyle modification as a control [83]. In contrast, 27 trials adopted EAHM and CM combination therapy as an intervention [60,62,63,66,67,68,69,71,72,73,74,77,78,80,84,85,89,91,92,94,96,97,98,103,104,105,106]. In all studies that adopted EAHM and CM combination therapy as an intervention, CM was used as a control group. Twenty-one trials included the ACP in herbal formulae [63,68,71,72,73,76,79,80,83,84,85,86,90,92,94,96,98,101,102,103,106]. Detailed information on the EAHM formulae, including ingredients, dosage, preparation type, and administration route, is provided in Supplementary Table S2. The interventions adopted as controls in the various trials were as follows: methylcobalamin (MCB, n = 27) [59,60,61,62,63,64,66,67,68,72,76,78,80,81,82,85,88,89,91,92,93,94,95,97,102,103,104], epalrestat (ERT, n = 7) [77,79,86,90,96,99,105], α-lipoic acid (ALA, n = 4) [69,74,100,106], methylcobalamin plus epalrestat (MpE, n = 2) [87,98], gabapentin (GBP, n = 1), nimodipine (NMD, n = 1) [101], methylcobalamin plus α-lipoic acid injection (MpA, n = 1) [73], methylcobalamin plus gabapentin (MpG, n = 1) [101], vitamin B1 plus vitamin B6 (V1pV6, n = 1) [65], methylcobalamin plus vitamin B1 plus vitamin B6 (MpV1pV6, n = 1) [70], adenosylcobalamin plus oryzanol plus vitamin B1 (ApOpV1, n = 1). [75] All included studies reported treatment duration, which ranged from 4 to 24 weeks, with 13 studies adopting a treatment period of ≥12 weeks [63,64,70,77,79,81,84,86,87,96,100,102,105].

### 3.3. Risk of Bias

The methodological quality of the 48 included studies is summarized in Table 2. The risk of bias in studies was assessed using the RoB 2 tool [46]. All the included studies had a high risk of bias in one or more domains. According to the RoB 2 evaluation criteria, the “overall risk of bias” is also regarded as high if the risk of bias is assessed to be high even for one domain. The overall risk of bias for all studies included in this review was also considered high. Studies that were rated as having a “high” overall risk of bias frequently lacked information on the randomization method, and the absence of a preregistered protocol made it difficult to address concerns about selective outcome reporting. Additionally, as most studies lacked a blinded design, every variation in the intended intervention had a substantial risk of bias.

**Table 1:** Basic demographic data and intervention of studies includes in the review.

Included Study (Reference)	Type of Diabetes/Diagnosis Criteria	Trial Design/Randomization Method	Number of Participants (Male/Female); Age (Mean ± SD)		Interventions		Morbidity Period (Mean ± SD or Range)		Outcome Index (Intergroup Differencies *p*-Value)	Course of Treatment	Adverse Event (Case/Symptom)
			Trial	Control	Trial	Control	Trial	Control			
Jin 2004 [59]	T1DM and T2DM/ WHO criteria, 1999	Randomized; Single center; Parallel/NR	103 (54/49) 59.4 ± 5.61 y	99 (51/48) 58.81 ± 6.01 y	Tangmaitong tablets (0.5 g × 4 t, t.i.d.)	Mecobalamin tablets (500 mg, t.i.d.)	3.31 ± 1.25 y	3.82 ± 1.17 y	1. MMNCV (*p* > 0.05) 2. MSNCV (*p* < 0.01) 3. PMNCV (*p* < 0.05) 4. PSNCV (*p* < 0.01)	8 w	Trial: 1 AE/diarrhea Control: 3 AEs/abdomnial pain with diarrhea
Sun 2008 [60]	T2DM/WHO criteria, 1999	Randomized; Single center; Parallel/NR	30 (18/12) 40~70 y	30 (16/14) 43~69 y	1. Ziyinbushenhuoxuetonglou fang decoction (300 mL, b.i.d.) 2. Mecobalamin tablets (500 mg, t.i.d.)	Mecobalamin tablets (500 mg, t.i.d.)	1~33 m	1~34 m	1. Response rate (*p* < 0.05)	4 w	NR
Shen 2009 [61]	T2DM/WHO criteria, 1999	Randomized; Single center; Parallel/Block randomization method	50 (21/29) 60 ± 4.2 y	50 (27/23) 58.81 ± 6.01 y	Tangmaining capsule (4.5 g × 5 c, b.i.d.)	Mecobalamin tablets (500 mg, t.i.d.)	8.5 y	7.9 y	1. Response rate (*p* < 0.05) 2. MMNCV (*p* < 0.05) 3. MSNCV (*p* < 0.05) 4. UMNCV (*p* < 0.01) 5. USNCV (*p* < 0.01) 6. PMNCV (*p* < 0.05) 7. PSNCV (*p* > 0.05) 8. TMNCV (*p* > 0.05) 9. TSNCV (*p* < 0.01)	8 w	Trial: No AE Control: No AE
Lin 2010 [62]	T2DM/WHO criteria, 1999	Randomized; Single center; Parallel/NR	40 (22/18) median 55.6 y	40 (23/19) median 54.2 y	1. Tongxinluo capsule (3c, t.i.d.) 2. Mecobalamin tablets (500 mg, t.i.d.)	Mecobalamin tablets (500 mg, t.i.d.)	NR	NR	1. Response rate (*p* < 0.05) 2. PMNCV (*p* < 0.01) 3. PSNCV (*p* < 0.01) 4. TMNCV (*p* < 0.01) 5. TSNCV (*p* < 0.01)	4 w	NR
Wang 2010 [63]	T2DM/WHO criteria, 1999	Randomized; Single center; Parallel/Simple randomization using random number table	80 (45/35) 62.68 ± 7.35 y	79 (43/36) 62.78 ± 7.57 y	1. Huangqiguizhiwuwu decoction (300 mL, b.i.d.) 2. Mecobalamin injection (0.5 mg, q.d., i.m.)	Mecobalamin injection (0.5 mg, q.d., i.m.)	7.12 ± 4.25 y	6.98 ± 4.62 y	1. Response rate (*p* < 0.01) 2. MMNCV (*p* < 0.01) 3. MNSCV (*p* < 0.01) 4. PMNCV (*p* < 0.01) 5. PSNCV (*p* < 0.01)	12 w	NR
Yan 2010 [64]	T2DM/WHO criteria, 1999	Randomized; Single center; Parallel/NR	14 (7/7) 57.79 ± 6.73 y	15 (6/9) 52.53 ± 8.0 y	Shutangluofang granule (b.i.d.)	Methylcobalamine (500 mg, t.i.d.)	13.14 ± 10.58 m	10.67 ± 11.14 m	1. Response rate (*p* < 0.05)	12 w	NR
Wu 2011 [65]	T1DM and T2DM/Guidelines for the Prevention and Treatment of Diabetes in China, 2004	Randomized; Single center; Parallel/NR	30 (16/14) mean 49.9 y	27 (15/12) mean 48 y	Modified yiqihuoxue decoction (300 mL, b.i.d.)	Vitamin B1 (20 mg, t.i.d.) Vitamin B6 (20 mg, t.i.d.)	mean 12 m	mean 11.4 m	1. Response rate (*p* < 0.01) 2. PMNCV (*p* < 0.01) 3. PSNCV (*p* < 0.01)	6 w	NR
Gao 2012 [66]	T2DM/WHO criteria, 1999	Randomized; Single center; Parallel/NR	30 (16/14) NR	30 (17/13) NR	1. Nourishing the liver to stop the wind and tongluo decoction 2. Methylcobalamine (0.5 mg, t.i.d.)	Methylcobalamine (0.5 mg, t.i.d.)	NR	NR	1. Response rate (*p* < 0.05) 2. MMNCV (*p* < 0.01) 3. MSNCV (*p* < 0.01) 4. PMNCV (*p* < 0.01) 5. PSNCV (*p* < 0.01)	8 w	Trial: 2 AEs/ nausea, upper abdominal discomfort Control: No AE
Gong 2013 [67]	T1DM and T2DM/WHO criteria, 1999	Randomized; Single center; Parallel/NR	60 (32/28) 56.42 ± 5.28 y	60 (33/27) 57.16 ± 5.34 y	1. Modified aconiti decoction (400 mL, b.i.d.) 2. Methylcobalamine (500 mg, t.i.d.)	Methylcobalamine (500 mg t.i.d.)	7.65 ± 3.84 m	7.83 ± 3.29 m	1. Response rate (*p* < 0.05) 2. PMNCV (*p* < 0.01) 3. PSNCV (*p* > 0.05)	30 d	Trial: No AE Control: No AE
Han 2013 [68]	T1DM and T2DM/WHO criteria, 1999	Randomized; Single center; Parallel/Simple randomization using random number table	31 (17/14) 54.2 ± 9.6 y	31 (16/15) 55.3 ± 10.1 y	1. Modified huangqiguizhiwuwu decoction (400 mL, b.i.d.) 2. Methylcobalamine (0.5 mg, t.i.d.)	Methylcobalamine (0.5 mg, t.i.d.)	NR	NR	1. Response rate (*p* < 0.05) 2. PMNCV (*p* < 0.01) 3. PSNCV (*p* < 0.01) 4. MMNCV (*p* < 0.01) 5. MSNCV (*p* < 0.01)	8 w	NR
Zhang 2013a [69]	T2DM/WHO criteria, 1999	Randomized; Single center; Parallel/NR	30 (16/14) 54.32 ± 7.14 y	30 (15/15) 56.24 ± 7.40 y	1. Mudan tong luo fang (b.i.d.) 2. α-Lipoic acid injection (600 mg, q.d., i.v. drip)	α-Lipoic acid injection (600 mg, q.d., i.v. drip)	8.3 ± 1.67 y	8.5 ± 1.54 y	1. Response rate (*p* < 0.05) 2. MMNCV (*p* < 0.05) 3. MSNCV (*p* < 0.05) 4. PMNCV (*p* < 0.05) 5. PSNCV (*p* < 0.05)	3 w	NR
Zhang 2013b [70]	T1DM and T2DM/Only diagnostic criteria are presented without reference.	Randomized; Single center; Parallel/NR	30 Total 60 (36/14) 56 ± 8 y	30 Total 60 (36/14) 56 ± 8 y	Tang bao kang (20 pills, t.i.d.)	1. Methylcobalamine (500 mg, t.i.d.) 2. Vitamin B1 (30 mg, t.i.d.) 3. Vitamin B6 (30 mg, t.i.d.)	Total 5~10 y	Total 5~10 y	1. Response rate (*p* < 0.01) 2. MMNCV (*p* < 0.01) 3. MSNCV (*p* < 0.01) 4. UMNCV (*p* < 0.01) 5. USNCV (*p* < 0.01) 6. PMNCV (*p* < 0.01) 7. PSNCV (*p* < 0.01)	24 w	Trial: No AE Control: 1 AE/skin rash
Guo 2014 [71]	T1DM and T2DM/WHO criteria, 1999	Randomized; Single center; Parallel/NR	32 (19/13) 64.78 ± 8.90 y	32 (15/17) 65.59 ± 8.35 y	1. Modified huangqiguizhiwuwu decoction (b.i.d.) 2. Mecobalamin tablets (0.5 mg, t.i.d.) 3. Gabapentin (600 mg, t.i.d.)	1. Mecobalamin tablets (0.5 mg, t.i.d.) 2. Gabapentin (600 mg, t.i.d.)	NR	NR	1. Response rate (*p* < 0.01)	8 w	NR
Yang 2014a [73]	T2DM/Diagnostic criteria of Chinese guidelines for the prevention and treatment of type 2 diabetes, 2008	Randomized; Single center; Parallel/NR	60 (35/25) 51.30 ± 6.03 y	60 (37/23) 51.26 ± 5.38 y	1. Shenqixuebi feng (b.i.d.) 2. α-Lipoic acid injection (0.3 g, q.d., i.v. drip) 3. Mecobalamin injection (0.5 mg, q.d., i.v. drip)	1. α-Lipoic acid injection (0.3 g, q.d., i.v. drip) 2. Mecobalamin injection (0.5 mg, q.d., i.v. drip)	3.65 ± 1.12 y	3.36 ± 1.18 y	1. Response rate (*p* < 0.05)	4 w	NR
Yang 2014b [72]	T1DM and T2DM/WHO criteria, 1999	Randomized; Single center; Parallel/NR	36 (23/13) 47.8 ± 8.3 y	36 (20/16) 46.5 ± 8.1 y	1. Modified huangqiguizhiwuwu decoction (200 mL, q.d.) 2. Methylcobalamine injection (500 mg, q.d., i.m.)	1. Methylcobalamine injection (500 mg, q.d., i.m.)	4.1 ± 1.3 m	3.9 ± 1.4 m	1. Response rate (*p* < 0.05)	4 w	NR
Qi 2015 [74]	T1DM and T2DM/WHO criteria, 1999	Randomized; Single center; Parallel/NR	32 (17/15) 53.2 ± 7.1 y	32 (16/16) 52.4 ± 7.0 y	1. Mudan granule (7 g, t.i.d.) 2. 0.9% Sodium chloride 200 mL + α-Lipoic acid injection (450 mg, q.d., i.v. drip)	1. 0.9% Sodium chloride 200 mL + α-Lipoic acid injection (450 mg, q.d., i.v. drip)	2.3 ± 2.1 y	2.6 ± 1.9 y	1. Response rate (*p* < 0.05) 2. PMNCV (*p* < 0.01) 3. PSNCV (*p* < 0.01)	4 w	Trial: No AE Control: No AE
Wang 2015 [75]	T2DM/TCM diagnosis and treatment plan for 95 diseases in 22 specialties	Randomized; Single center; Parallel/NR	40 (20/20) mean 68.5 y	40 (23/17) mean 71.2 y	1. Yinxinshu capsule (3c, t.i.d.) 2. Maixuekang capsule (3c, t.i.d.)	1. Oryzanol (20 mg, t.i.d.) 2. Vitamin B1 (10 mg, t.i.d.) 3. Adenosylcobalamin (1 mg, t.i.d.)	10~12 y	10~12 y	1. Response rate (*p* < 0.05)	4 w	Trial: No AE Control: No AE
Xue 2015 [76]	T2DM/WHO criteria, 1999	Randomized; Single center; Parallel/Simple randomization using random number table	42 (23/19) 36~78 y	42 (22/20) 35~78 y	1. Modified liutengshuilushexian decoction (150 mL, q.d.)	1. Methylcobalamine tablet (0.5 mg, t.i.d.)	28~73 d	30~73 d	1. Response rate (*p* < 0.01) 2. MSNCV (*p* < 0.01) 3. TSNCV (*p* < 0.01) 4. PSNCV (*p* < 0.01)	3 w	Trial: No AE Control: No AE
Guo 2016 [77]	T1DM and T2DM/Diabetic peripheral neuropathy diagnosis and treatment guidelines of China, 2009	Randomized; Single center; Parallel/Simple randomization using random number table	51 (26/25) 69.54 ± 5.06 y	51 (28/23) 69.78 ± 5.96 y	1. Qitengtongluo decoction (b.i.d.) 2. Epalrestat (50 mg, 1t, t.i.d.)	1. Epalrestat (50 mg, 1 t, t.i.d.)	1.91 ± 2.09 y	6.59 ± 1.91 y	1. Response rate (*p* < 0.05) 2. NCSS (*p* < 0.05) 3. MSNCV (*p* < 0.05) 4. TSNCV (*p* < 0.05) 5. PMNCV (*p* < 0.05) 6. PSNCV (*p* < 0.05)	12 w	NR
Han 2016 [78]	TIDM and T2DM/Diagnostic criteria for diabetic peripheral neuropathy formulated by the Chinese Medical Doctor Association	Randomized; Single center; Parallel/NR	20 (12/8) 54.3 ± 7.2 y	20 (11/9) 53.7 ± 6.8 y	1. Zhanjin tongluo Chinese medicine (b.i.d.) 2. Mecobalamin tablets (500 mg, t.i.d.)	1. Mecobalamin tablets (500 mg, t.i.d.)	2.4 ± 1.2 y	2.6 ± 1.3 y	1. Response rate (*p* < 0.05)	4 w	NR
Lan 2016 [79]	T2DM/WHO criteria, 1999	Randomized; Single center; Parallel/NR	54 Other information NR	54 Other information NR	Yiqihuoxue tongluo capsule (1.2 g, t.i.d.)	Epalrestat tablets (50 mg, t.i.d.)	NR	NR	1. Response rate (*p* < 0.05) 2. PMNCV (*p* < 0.05)	12 w	Trial: No AE Control: No AE
Mo 2016 [82]	T2DM/Guidelines for the Prevention and Treatment of Type 2 Diabetes in China, 2013	Randomized; Single center; Parallel/Simple randomization using random number table	33 (19/14) 65.28 ± 9.098 y	32 (17/15) 62.34 ± 8.168 y	Yangyinjiedu decoction (300 mL, b.i.d.)	Methylcobalamine (0.5 mg t.i.d.)	2~23 y	2~19 y	1. Response rate (*p* < 0.01)	8 w	NR
Wang 2016 [83]	T2DM/Guidelines for the Prevention and Treatment of Type 2 Diabetes in China, 2013	Randomized; Single center; Parallel/Simple randomization using random number table	124 (72/52) 57.3 ± 6.8 y	103 (58/45) 58.1 ± 7.2 y	Modified tangbitong feng (150 mL, b.i.d.)	Lifestyle modification	22.1 ± 5.4 m	23.5 ± 4.8 m	1. Response rate (*p* < 0.01)	8 w	Trial: No AE Control: No AE
Li 2016a [80]	T1DM and T2DM/Diabetic peripheral neuropathy diagnosis and treatment of China, 2009	Randomized; Single center; Parallel/Simple randomization using random number table	30 (18/12) 49.6 ± 5.6 y	30 (17/13) 50.3 ± 5.4 y	1. Wenyanghuoxuetongbi feng (b.i.d.) 2. Methylcobalamine (0.5 mg, t.i.d.)	1. Methylcobalamine (0.5 mg, t.i.d.)	18.21 ± 12.37 m	17.97 ± 12.54 m	1. Response rate (*p* < 0.01) 2. TSNCV (*p* < 0.01) 3. PSNCV (*p* < 0.05)	8 w	Trial: No AE Control: No AE
Zhang 2016a [85]	T1DM and T2DM/WHO criteria, 1999	Randomized; Single center; Parallel/Simple randomization using random number table	48 (26/22) 54.6 y	48 (28/20) 55.2 y	1. Huangichifeng decoction combined Dangguisini decoction (q.d.) 2. Methylcobalamine injection (500 mg, q.d., i.m.)	1. Methylcobalamine injection (500 mg, q.d., i.v.)	2.8 y	3.2 y	1. Response rate (*p* < 0.01) 2. MSNCV (*p* < 0.01) 3. USNCV (*p* < 0.01) 4. PMNCV (*p* < 0.01) 5. TMNCV (*p* < 0.01)	4 w	NR
Li 2016b [81]	T2DM/WHO criteria, 1999	Randomized; Single center; Parallel/NR	60 (37/23) 57 y	60 (35/25) 56 y	Huangzhitongnaoluo capsule (3c, t.i.d.)	Mecobalamin dispersible tablets (500 mg, t.i.d.)	1~13 y	1~12 y	1. Response rate (*p* < 0.05) 2. MSNCV (*p* < 0.05) 3. TMNCV (*p* < 0.05)	12 w	NR
Zhang 2016b [84]	T2DM/WHO criteria, 1999	Randomized; Single center; Parallel/Simple randomization using random number table	60 (36/24) 55.3 ± 6.4 y	60 (35/25) 55.6 ± 5.5 y	1. Qiming granule (4.5 g, t.i.d.) 2. Nimodipine injection (8 mg, q.d., i.v. drip)	1. Nimodipine injection (8 mg, q.d., i.v. drip)	2.0 ± 1.1 y	2.2 ± 1.0 y	1. Response rate (*p* < 0.01) 2. MMNCV (*p* < 0.01) 3. MSNCV (*p* < 0.01) 4. UMNCV (*p* < 0.05) 5. USNCV (*p* < 0.01) 6. TMNCV (*p* < 0.05) 7. TSNCV (*p* < 0.01)	12 w	Trial: No AE Control: 1 AE/mild dizziness
Chen 2017 [86]	T2DM/Guidelines for the Prevention and Treatment of Type 2 Diabetes in China, 2013	Randomized; Single center; Parallel/Simple randomization using random number table	30 (14/16) 38.72 ± 20.02 y	30 (13/17) 39.11 ± 19.57 y	Dagguisini decoction (300 mL, b.i.d.)	Epalrestat capsule (50 mg, t.i.d.)	4.32 ± 2.05 y	4.20 ± 2.01 y	1. Response rate (*p* < 0.05)	12 w	Trial: No AE Control: No AE
Shi 2017 [87]	T2DM/WHO criteria, 1999	Randomized; Single center; Parallel/NR	32 (20/12) 38.7 ± 8.1 y	32 (22/10) 40.3 ± 10.1 y	1. Fufang danshen dripping pill (10 pill, t.i.d.)	1. Methylcobalamine (0.5 mg, t.i.d.) 2. Epalrestat (50 mg, t.i.d.)	3.87 ± 1.5 y	3.69 ± 1.3 y	1. TSNCV (*p* < 0.01)	15 w	NR
Wang 2017 [88]	T2DM/WHO criteria, 1999	Randomized; Single center; Parallel/Simple randomization using random number table	30 (15/15) 58.76 ± 4.32 y	30 (16/14) 57.21 ± 3.56 y	Dangguisini decoction (200 mL, b.i.d.)	Mecobalamin tablets (500 mg, t.i.d.)	3.56 ± 1.21 y	3.84 ± 1.36 y	1. Response rate (*p* < 0.05) 2. MMNCV (*p* > 0.05) 3. MSNCV (*p* > 0.05) 4. PMNCV (*p* < 0.05) 5. PSNCV (*p* < 0.05) 6. TMNCV (*p* < 0.05) 7. TSNCV (*p* < 0.05)	8 w	NR
Chen 2018 [89]	T2DM/WHO criteria, 1999	Randomized; Single center; Parallel/Simple randomization using random number table	40 (19/21) 55.8 ± 4.7 y	40 (20/20) 56.2 ± 2.8 y	1. Dangguisinin decoction (b.i.d.) 2. Mecobalamin tablets (500 mg, t.i.d.)	Mecobalamin tablets (500 mg, t.i.d.)	3.6 ± 1.8 y	2.4 ± 2.1 y	1. Response rate (*p* < 0.05)	4 w	Trial: 2 AEs/skin rash, gastrointestinal discomfort Control: 3 AEs/diarrhea (2), skin rash
Dai 2018 [90]	T2DM/Guidelines for the Prevention and Treatment of Type 2 Diabetes in China, 2013	Randomized; Single center; Parallel/NR	40 45~85 y Other information NR	40 45~85 y Other information NR	Modified huangqiguizhiwuwu decoction (500 mL, b.i.d.)	Epalrestat capsule (50 mg, t.i.d.)	NR	NR	1. Response rate (*p* < 0.05) 2. UMNCV (*p* < 0.05) 3. USNCV (*p* < 0.05) 4. PMNCV (*p* < 0.05) 5. PSNCV (*p* < 0.05)	3 w	NR
Hu 2018 [92]	T2DM/Guidelines for the Prevention and Treatment of Type 2 Diabetes in China, 2013	Randomized; Single center; Parallel/NR	31 (13/18) 55.45 ± 11.52 y	31 (15/16) 53.76 ± 2.03 y	1. Modified jiajianhuangqiguizhiwuwu decoction (200 mL, b.i.d.) 2. Methylcobalamine (0.5 mg, t.i.d.)	1. Methylcobalamine tablet (0.5 mg, t.i.d.)	7.13 ± 2.01 y	6.52 ± 1.95 y	1. Response rate (*p* < 0.05) 2. PMNCV (*p* < 0.05)	8 w	NR
Huang 2018 [93]	T1DM and T2DM/Diagnostic and therapeutic effect evaluation criteria of diseases and syndromes in traditional Chinese medicine, 1994	Randomized; Single center; Parallel/Simple randomization using random number table	120 (52/68) 51.3 ± 11.4 y	120 (51/69) 50.9 ± 11.6 y	Matong powder (7 g, t.i.d.)	Methylcobalamine tablet (0.5 mg, t.i.d.)	8.92 ± 8.6 m	8.97 ± 8.5 m	1. Response rate (*p* < 0.05) 2. PMNCV (*p* < 0.05) 3. TSNCV (*p* < 0.05)	8 w	Trial: 3 AEs/ Abdominal bloating with anorexia (3) Control: 2 AEs/Abdominal bloating with anorexia (2)
She 2018 [94]	T2DM/Guidelines for the Prevention and Treatment of Type 2 Diabetes in China, 2010	Randomized; Single center; Parallel/Simple randomization using random number table	30 (18/12) 63.35 ± 7.12 y	30 (17/13) 65.13 ± 6.21 y	1. Huangqiguizhiwuwu granule (b.i.d.) 2. Mecobalamin tablet (1 mg, t.i.d.)	Mecobalamin tablet (1 mg, t.i.d.)	3.31 ± 2.06 y	3.82 ± 1.97 y	1. Response rate (*p* < 0.05)	6 w	NR
Xin 2018 [95]	T2DM/Diabetic peripheral neuropathy diagnosis and treatment of China, 2009	Randomized; Single center; Parallel/NR	30 Total 60 (36/24) 55.3 y	30 Total 60 (36/24) 55.3 y	1. Mongolian medicine garidi-13 weiwan (3 g, q.d.)	Mecobalamin tablet (0.5 mg, t.i.d.)	Total 4.2 y	Total 4.2 y	1. Response rate (*p* < 0.05)	4 w	NR
Gao 2019 [91]	T1DM and T2DM/WHO criteria, 1999	Randomized; Single center; Parallel/NR	50 (26/24) 60.83 ± 5.26 y	50 (25/25) 61.17 ± 6.05 y	1. Modified shegmaisan (300 mL, b.i.d.) 2. Mecobalmin tablet (500 mg, t.i.d.)	Mecobalmin tablet (500 mg, t.i.d.)	3.82 ± 1.04 y	3.77 ± 1.12 y	1. Response rate (*p* < 0.05) 2. MMNCV (*p* > 0.05) 3. MSNCV (*p* > 0.05) 4. PMNCV (*p* < 0.05) 5. PSNCV (*p* < 0.05) 6. TMNCV (*p* < 0.05) 7. TSNCV (*p* < 0.05)	8 w	Trial: No AE Control: No AE
Wu 2019 [99]	T2DM/Guidelines for the Prevention and Treatment of Type 2 Diabetes in China, 2013	Randomized; Single center; Parallel/Simple randomization using random number table	30 (16/14) 57.60 ± 7.20 y	30 (16/14) 57.03 ± 7.63 y	Taohongsiwu decoction (t.i.d.)	Epalrestat tablet (50 mg, t.i.d.)	4.3 y	4.3 y	1. Response rate (*p* < 0.05) 2. MSNCV (*p* < 0.05) 3. PSNCV (*p* < 0.05)	4 w	Trial: No AE Control: No AE
Yi 2019 [100]	T1DM and T2DM/Diabetic neuropathy diagnosis criteria of American Diabetes Association, 2017	Randomized; Single center; Parallel/Simple randomization using random number table	60 (31/29) 61.36 ± 4.37 y	60 (29/31) 61.53 ± 4.64 y	Mongolian medicine zhenbo pills (0.2 g × 15 p, b.i.d.)	α-Lipoic acid tablet (0.3 g × 2 c, q.d.)	8.23 ± 3.21 y	8.23 ± 3.12 y	1. MMNCV (*p* < 0.05) 2. MSNCV (*p* < 0.05) 3. PMNCV (*p* < 0.05) 4. PSNCV (*p* < 0.05)	24 w	Trial: 5 AEs/ nausea (2), anorexia (3) Control: 6 AEs/ nausea (2), gastric pain (2)
Ji 2019 [96]	T2DM/Guidelines for the Prevention and Treatment of Type 2 Diabetes in China, 2010	Randomized; Single center; Parallel/Simple randomization using random number table	54 (32/22) 54.47 ± 9.81 y	53 (33/20) 54.81 ± 9.44 y	1. Yangyinzhuyu decoction (150 mL, b.i.d.) 2. Epalrestat tablet (50 mg, t.i.d.)	Epalrestat tablet (50 mg, t.i.d.)	10.24 ± 3.08 y	10.53 ± 2.66 y	1. Response rate (*p* < 0.05)	90 d	Trial: No AE Control: No AE
Liu 2019a [97]	T2DM/Guidelines for the Prevention and Treatment of Type 2 Diabetes in China, 2013	Randomized; Single center; Parallel/Simple randomization using random number table	40 Other information NR	40 Other information NR	1. Shengjinsan combined Taohongyin (200 mL, b.i.d.) 2. Mecobalamin tablet (500 mg, t.i.d.)	Mecobalamin tablet (500 mg, t.i.d.)	NR	NR	1. MMNCV (*p* < 0.05) 2. MSNCV (*p* < 0.05) 3. TMNCV (*p* < 0.05) 4. TSNCV (*p* < 0.05)	4 w	NR
Liu 2019b [98]	T2DM/Guidelines for the Prevention and Treatment of Type 2 Diabetes in China, 2013	Randomized; Single center; Parallel/Simple randomization	45 (27/18) 58.77 ± 4.26 y	45 (26/19) 59.46 ± 4.77 y	1. Huangqiguizhiwuwu decoction (400 mL, b.i.d.) 2. Epalrestat tablets (t.i.d.) 3. Mecobalamin tablet (t.i.d.)	1. Epalrestat tablets (t.i.d.) 2. Mecobalamin tablet (t.i.d.)	3.28 ± 1.45 m	3.31 ± 1.13 m	1. Response rate (*p* < 0.05)	8 w	NR
Chen 2021 [101]	T2DM/Guidelines for the Prevention and Treatment of Type 2 Diabetes in China, 2013	Randomized; Single center; Parallel/NR	28 (15/13) 57.2 ± 8.1 y	29 (16/13) 56.5 ± 7.6 y	1. Zicuijuanbi decoction (150 mL, b.i.d.) 2. Normal saline injection (250 mL, i.v.)	1. gabapentin capsule (0.3 g, t.i.d.) 2. Normal saline injection (250 mL, i.v.)	15.57 ± 3.68 y	14.59 ± 4.35 y	1. Response rate (*p* < 0.05)	10 w	NR
Hou 2021 [102]	T2DM/WHO criteria, 1999	Randomized; Single center; Parallel/NR	39 (24/15) 56.74 ± 11.79 y	28 (18/10) 55.83 ± 10.60 y	Jiuchongdan (40 pills, t.i.d.)	Mecobalamin tablet (500 mg, t.i.d.)	15.28 ± 11.23 m	16.72 ± 10.96 m	1. Response rate (*p* < 0.05) 2. PSNCV (*p* < 0.05) 3. MSNCV (*p* < 0.05) 4. USNCV (*p* < 0.05)	12 w	NR
Li 2021 [103]	T2DM/Guidelines for the Prevention and Treatment of Diabetic Peripheral Neuropathy by Traditional Chinese Medicine, 2011	Randomized; Single center; Parallel/Simple randomization using random number table	41 (22/19) 59.81 ± 5.63 y	41 (23/18) 60.20 ± 5.62 y	1. Huangqiguizhiwuwu decoction (200 mL, t.i.d.) combined Mudan granule (7 g, t.i.d.) 2. Mecobalmin tablet (500 mg, t.i.d.)	1. Mecobalamin tablet (500 mg, t.i.d.)	3.15 ± 0.45 y	3.12 ± 0.43 y	1. Response rate (*p* < 0.05) 2. MMNCV (*p* < 0.05) 3. MSNCV (*p* < 0.05) 4. PMNCV (*p* < 0.05) 5. PSNCV (*p* < 0.05)	8 w	Trial: 5 AEs/diarrhea (1), nausea (1), constipation (2), dizziness (1) Control: 1 AE/ nausea (1)
Wang 2021a [105]	T1DM and T2DM/diagnostic criteria are presented without reference	Randomized; Single center; Parallel/Simple randomization using random number table	30 (16/14) 64.63 ± 4.72 y	30 (17/13) 64.71 ± 4.68 y	1. Yiqiyangyintongluo decoction (200 mL, b.i.d.) 2. Epalrestat tablets (50 mg, t.i.d.)	1. Epalrestat tablets (50 mg, t.i.d.)	6.14 ± 1.24 y	6.12 ± 1.22 y	1. Response rate (*p* < 0.05)	12 w	NR
Wang 2021b [104]	T2DM/diagnostic criteria are presented without reference	Randomized; Single center; Parallel/Simple randomization using random number table	50 (34/16) 67.13 ± 6.29 y	50 (32/18) 67.13 ± 6.29 y	1. Taohongsiwu decoction (b.i.d.) 2. Mecobalmin capsule (0.5 mg, t.i.d.)	1. Mecobalamin capsule (0.5 mg, t.i.d.)	1.57 ± 0.51 y	1.42 ± 0.83 y	1. MMNCV (*p* < 0.05) 2. MSNCV (*p* < 0.05) 3. PMNCV (*p* < 0.05) 4. PSNCV (*p* < 0.05) 5. TMNCV (*p* < 0.05) 6. TSNCV (*p* < 0.05)	4 w	NR
Zhang 2021 [106]	T2DM/Guidelines for the Prevention and Treatment of Diabetic Peripheral Neuropathy by Traditional Chinese Medicine, 2011	Randomized; Single center; Parallel/NR	74 Total 148 (78/70) 59.64 ± 8.94 y	74 Total 148 (78/70) 59.64 ± 8.94 y	1. Buqi Huoxue Zhitong decoction (b.i.d.) 2. α-Lipoic acid injection (0.6 g, q.d.) combined 0.9% Sodium chloride injection (250 mL, q.d.)	1. α-Lipoic acid injection (0.6 g, q.d.) combined 0.9% Sodium chloride injection (250 mL, q.d.)	Total 9.33 ± 1.25 y	Total 9.33 ± 1.25 y	1. TSNCV (*p* < 0.05) 2. PSNCV (*p* < 0.05)	8 w	NR

AEs: adverse events; b.i.d.: bis in die; c: capsules; d: days; EAHM: East Asian herbal medicine; g: grams; i.v.: intravenous; m: months; mg: milligrams; MMNCV: median motor nerve conduction velocity; MSNCV: median sensory nerve conduction velocity; NR: not reported; *p*: packs; p.o.: per os; PMNCV: peroneal motor nerve conduction velocity; PSNCV: peroneal sensory nerve conduction velocity; q.d: quaque die; SD: standard deviation; t: tablets; t.i.d.: ter in die; T1DM: type one diabetes mellitus; T2DM: type two diabetes mellitus; TMNCV: tibial motor nerve conduction velocity; TSNCV: tibial sensory nerve conduction velocity; UMNCV: ulnar motor nerve conduction velocity; USNCV: ulnar sensory nerve conduction velocity; w: weeks; WHO: World Health Organization; y: years; µg: microg.

### 3.4. Pairwise Meta-Analysis

A pairwise meta-analysis was conducted for each intervention (EACP, ECCP, EAWP, and ECWP) to evaluate the effects on the response rate, motor nerve conduction velocity (MNCV), and sensory nerve conduction velocity (SNCV) compared to the control group.

#### 3.4.1. Response Rate

**Figure 2:**
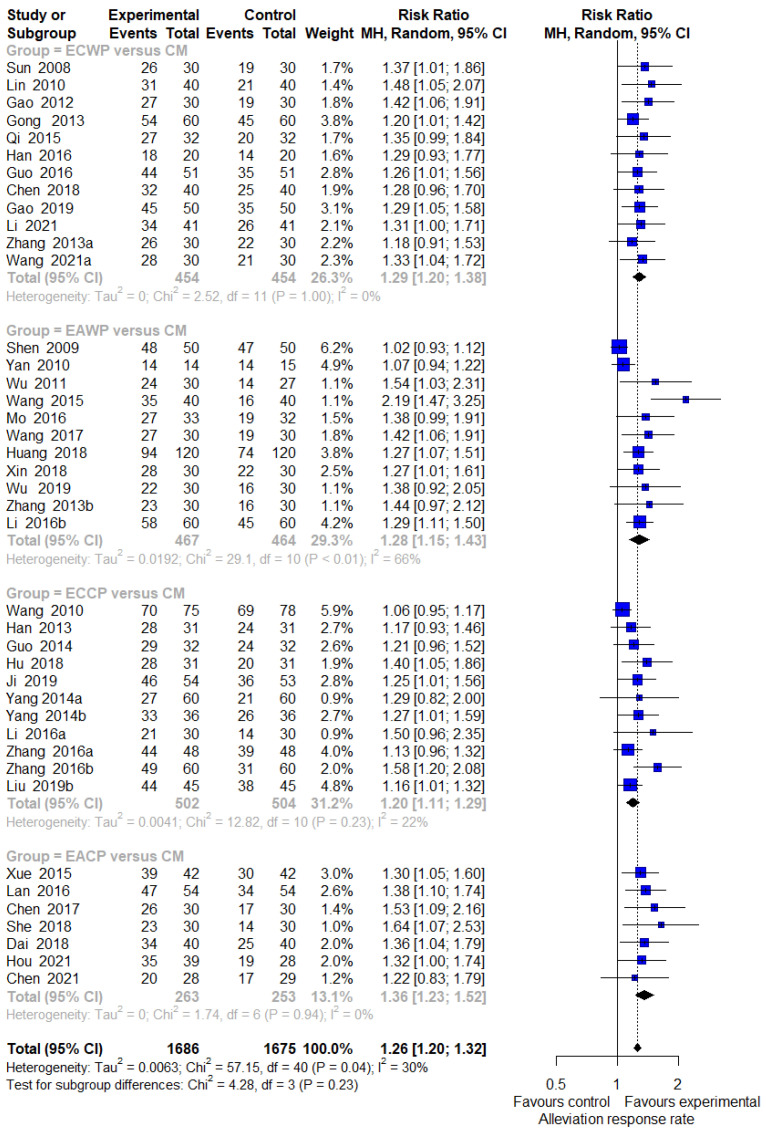
Forest plot of the trials that compared EAHM with CM for response rate. CI: confidence interval; CM: conventional medicine; EAHM: East Asian herbal medicine.

In seven studies comparing the effect of EACP with the CM control, EACP significantly improved the response rate compared with the CM control (7 trials, n = 516; RR:1.3629; 95% CI:1.2259 to 1.5151; *p* < 0.0001; I^2^ = 0%, *p* = 0.9420; Figure 2). In 12 trials, ECCP was significantly more effective than the CM control in terms of response rate (11 trials, n = 1006; RR:1.1978; 95% CI:1.1129 to 1.2892; *p* < 0.0001; I^2^= 22%, *p* = 0.2341; Figure 2). ECWP was superior to CM control in response rate (12 trials, n = 908; RR: 1.2863; 95% CI: 1.1959 to 1.3835; *p* < 0.0001; I^2^ = 0%, *p* = 0.9957; Figure 2). Compared with the CM control, EAWP demonstrated a superior response rate (11 trials, n = 931; RR:1.2830; 95% CI:1.1472 to 1.4349; *p* < 0.0001; I^2^ = 65.6%, *p* < 0.0012; Figure 2). One study evaluating the effect of EACP versus lifestyle modification control was excluded from the pairwise meta-analysis. In this study, EACP showed a stronger effect on the response rate than the CM control (1 trial, n = 227; RR:1.174; 95% CI:1.0221 to 1.3484; *p* < 0.01).

#### 3.4.2. Motor Nerve Outcomes: MMNCV, PMNCV, UMNCV, TMNCV

The meta-analysis results indicated that ECCP significantly increased MMNCV compared to the CM control (4 trials, n = 423; MD: 5.0142; 95% CI, 3.4682 to 6.5602; *p* < 0.0001; I^2^ = 74.6%, *p* = 0.0081; Supplementary Figure S1). ECWP remarkably increased MMNCV compared to the CM control (6 trials, n = 520; MD: 2.6593; 95% CI: 1.3840 to 3.9345; *p* < 0.0001; I^2^ = 70.6%, *p* = 0.0044; Supplementary Figure S1). EAWP also increased MMNCV compared with the CM control (4 trials, n = 422; MD: 1.6437; 95% CI: 0.7178 to 2.5696; *p* = 0.0005; I^2^ = 0%, *p* = 0.8350; Supplementary Figure S1).

Compared to CM control, EACP (2 trials, n = 188; MD: 3.2718; 95% CI: 2.0037 to 4.5364; *p* < 0.0001; I^2^ = 0%, *p* = 0.7087; Supplementary Figure S2) and ECCP (4 trials, n = 379; MD: 3.3977; 95% CI: 2.0446 to 4.7508; *p* < 0.0001; I^2^ = 61.2%, *p* = 0.0521; Supplementary Figure S2) increased PMNCV, and EAWP (6 trials, n = 782; MD: 2.2025; 95% CI: 1.1826 to 3.2225; *p* < 0.0001; I^2^ = 65.4%, *p* = 0.0130; Supplementary Figure S2) and ECWP significantly increased PMNCV (9 trials, n = 743; MD: 3.2034; 95% CI: 2.2196 to 4.1871; *p* < 0.0001; I^2^ = 81.9%, *p* < 0.0001; Supplementary Figure S2). 

Compared to the CM control, EACP (1 trial, n = 80; MD: 4.5500; 95% CI: 2.8743 to 6.2257; *p* < 0.0001; I^2^ = not applicable; Supplementary Figure S3) and ECCP (1 trial, n = 120; MD: 3.3000; 95% CI: 2.2787 to 4.3213; *p* < 0.0001; I^2^ = not applicable; Supplementary Figure S3) significantly increased UMNCV. In the two studies comparing the effect of EAWP with that of the CM control, EAWP significantly increased UMNCV compared to the CM control (2 trials, n = 160; MD:2.5186; 95% CI: 0.6061 to 4.4312; *p* < 0.0001; I^2^ = 61.6%, *p* = 0.1064; Supplementary Figure S3).

Compared to the CM control, both ECCP (2 trials, n = 216; MD: 2.9846; 95% CI:1.9157 to 4.0535; *p* < 0.0001, I^2^ = 0%; *p* = 0.6993; Supplementary Figure S4) and ECWP (4 trials, n = 370; MD: 3.7942; 95% CI:1.8227 to 5.7658; *p* =0.0002; I^2^ = 88.9%, *p* < 0.0001; Supplementary Figure S4) significantly increased TMNCV. In contrast, there was no significant difference between the effects of EAWP and the CM control on TMNCV (3 trials, n = 280; MD: 3.9412; 95% CI: −0.0158 to 7.8982; *p* = 0.0509; I^2^ = 94.2%, *p* < 0.0001; Supplementary Figure S4).

#### 3.4.3. Sensory Nerve Outcomes: MSNCV, PSNCV, USNCV, TSNCV

Compared to the CM control, both EACP (2 trials, n = 151; MD: 4.1171; 95% CI: 3.1335 to 5.1007; *p* < 0.0001; I^2^ = 0%, *p* = 0.3491; Supplementary Figure S5) and ECCP (4 trials, n = 437; MD: 4.9293; 95% CI: 4.1356 to 5.7229; *p* < 0.0001; I^2^ = 20.1%, *p* = 0.2893; Supplementary Figure S5) increased MSNCV. Compared to the CM control, EAWP (7 trials, n = 722; MD:2.4150; 95% CI: 1.1971 to 3.6329; *p* < 0.0001; I^2^ = 86.5%, *p* < 0.0001; Supplementary Figure S5) and ECWP (7 trials, n = 584; MD:2.2200; 95% CI:1.1962 to 3.2439; *p* < 0.0001; I^2^ = 76.5%, *p* = 0.0003; Supplementary Figure S5) significantly increased MSNCV. 

Compared to the CM control, both EACP (3 trials, n = 231; MD: 2.8905; 95% CI: 1.7993 to 3.9818; *p* < 0.0001; I^2^ = 5.5%, *p* = 0.3471; Supplementary Figure S6) and ECCP (5 trials, n = 511; MD:3.5114; 95% CI: 2.0661 to 4.9567; *p* < 0.0001; I^2^ = 83%, *p* = 0.0001; Supplementary Figure S6) significantly increased PSNCV. EAWP was superior to the CM control in increasing PSNCV (7 trials, n = 659; MD: 3.3038; 95% CI:2.0664 to 4.5413; *p* < 0.0001; I^2^ = 86%, *p* < 0.0001; Supplementary Figure S6). Compared with the CM control, ECWP was superior in increasing PNSCV (8 trials, n = 656; MD: 2.0450; 95% CI: 1.0524 to 3.0375; *p* < 0.0001; I^2^ = 80.7%, *p* < 0.0001; Supplementary Figure S6).

Compared to the CM control, EACP (2 trials, n = 147; MD: 3.4537; 95% CI: 1.5180 to 5.3895; *p* < 0.0001; I^2^ = 0%, *p* = 0.3843; Supplementary Figure S7) and ECCP (2 trials, n = 216; MD: 5.0567; 95% CI: 4.2339 to 5.8795; *p* < 0.0001; I^2^ = 0%, *p* = 0.9061; Supplementary Figure S7) significantly increased USNCV. EAWP was superior in increasing USNCV compared to the CM control (2 trials, n = 160; MD: 1.9357; 95% CI: 0.0310 to 3.8404; *p* < 0.0001; I^2^ = 68.8%, *p* = 0.0733; Supplementary Figure S7).

EACP (1 trial, n = 84; MD: 2.1000; 95% CI: 0.9369 to 3.2631; *p* = 0.0004; I^2^ = not applicable; Supplementary Figure S8), ECCP (3 trials, n = 328; MD: 4.5060; 95% CI: 3.3591 to 5.6592; *p* < 0.0001; I^2^ = 61.9%, *p* = 0.0724; Supplementary Figure S8), EAWP (4 trials; MD: 3.1575; 95% CI: 2.5478 to 3.7672; *p* < 0.0001; I^2^ = 0%, *p* = 0.7979; Supplementary Figure S8), and ECWP were significantly more effective than the CM control in increasing TSNCV (5 trials, n = 472; MD: 3.1596; 95% CI: 2.0694 to 4.2497; *p* < 0.0001; I^2^ = 79.2%, *p* = 0.0007; Supplementary Figure S8).

#### 3.4.4. Safety Assessment 

Of the studies included in this review, 20 reported adverse events [59,61,66,67,70,74,75,76,79,80,83,84,86,89,91,93,96,99,100,103]. Of these, 12 studies reported no adverse events in either the treatment or control group [61,67,74,75,76,79,80,83,86,91,96,99]. The adverse events reported in eight trials were mostly digestive disorders such as anorexia, nausea, abdominal blotting, and diarrhea. Additionally, skin rash was observed in two trials and mild dizziness was reported in one trial [70,84,89]. No serious adverse events were reported in any of the included trials, and no significant differences were observed in the frequency or characteristics of adverse events between the EAHM intervention and CM control groups. The details of all adverse events reported in each trial are summarized in Table 1. 

#### 3.4.5. Sensitivity Analysis

**Figure 3:**
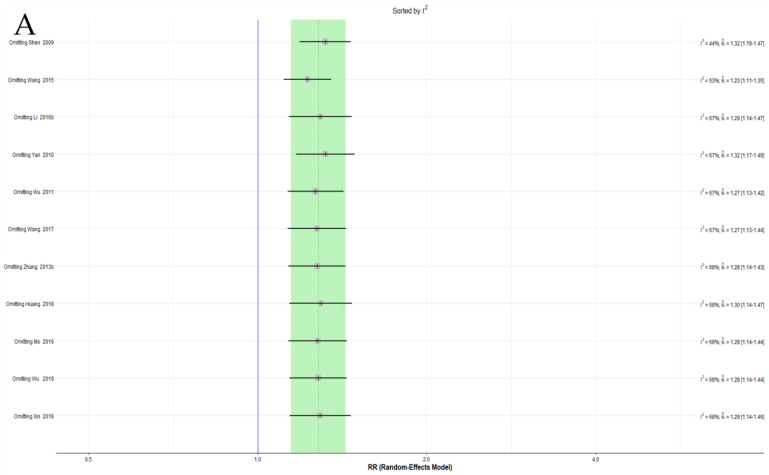

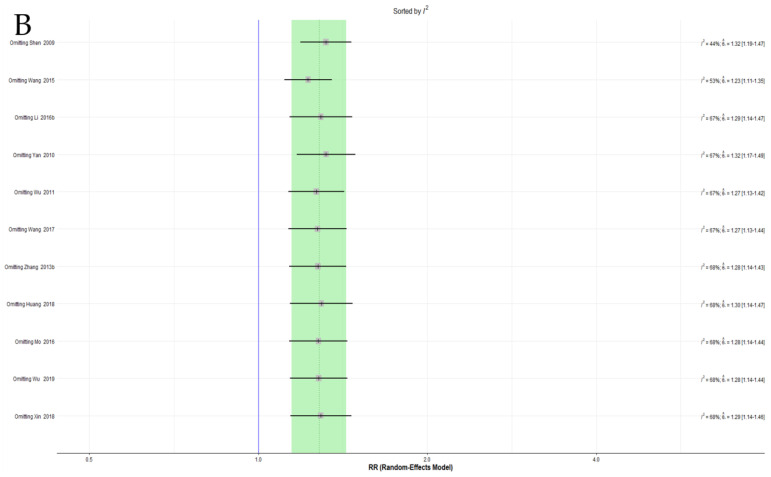
(**A**) Forest plot of the sensitivity analysis ordered by heterogeneity for response rate. (**B**) Forest plot of the sensitivity analysis ordered by effect size for the response rate.

More than 10 trials were included in the meta-analysis of the EAWP and CM on the response rate. Because severe heterogeneity was observed in this analysis, a sensitivity analysis of the leave-one-out method was performed, and one trial that significantly affected heterogeneity was identified [61]. However, this study did not show evident differences from other studies, and no separate effect on the overall effect size (Figure 3A,B). No additional sensitivity analysis was performed for the other pairwise meta-analysis items because there were no reports of more than 10 trials per outcome.

#### 3.4.6. Publication Bias

**Figure 4:**
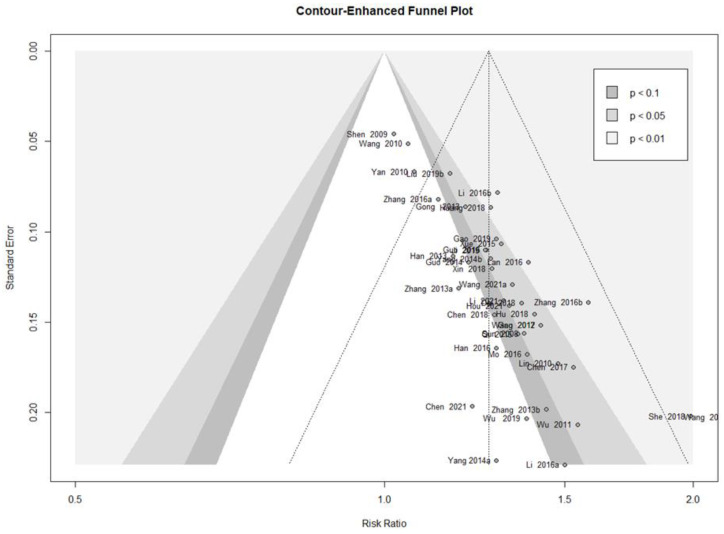
Contour-enhanced funnel plot of the response rate of the trials.

A contour-enhanced funnel plot analysis was performed to explore publication bias through the response rate, which was the outcome of most of the included studies. Since the pattern in the funnel plot displayed asymmetry, publication bias was deemed possible (Figure 4). This finding was further confirmed using Egger’s test (t = 10.10, df = 39, *p* < 0.0001) and Begg’s test (z = 4.23, *p* < 0.0001).

#### 3.4.7. Quality of Evidence According to Outcome Measures

In the comparison between the EAHM interventions and CM controls, the overall quality of evidence according to all outcome measures ranged from very low to moderate. The results of the GRADE assessment are presented in Table 3 and Table S3. 

### 3.5. Network Meta-Analysis 

**Figure 5:**
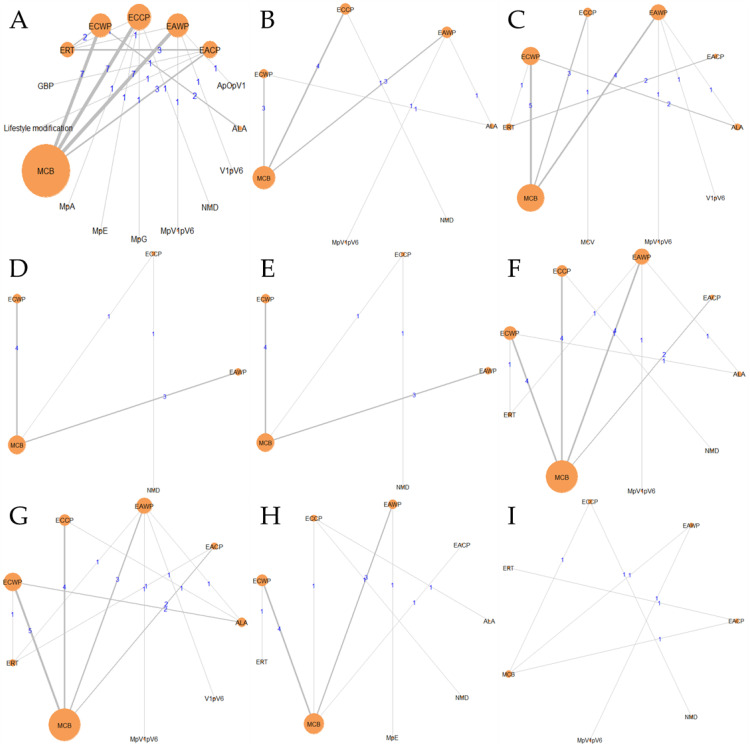
Geometry of the network: (**A**) response rate; (**B**) MMNCV; (**C**) PMNCV; (**D**) TMNCV; (**E**) UMNCV; (**F**) MSNCV; (**G**) PSNCV; (**H**) TSNCV; (**I**) USNCV.

NMA was performed for all 16 treatments, and the network relationships between the treatments for each outcome are shown in Figure 5. Detailed information is summarized in Table 4, including the number of interventions and networks for each outcome, the number of patients, whether the network is closed, and the number of direct comparisons.

#### 3.5.1. Response Rate

**Figure 6:**
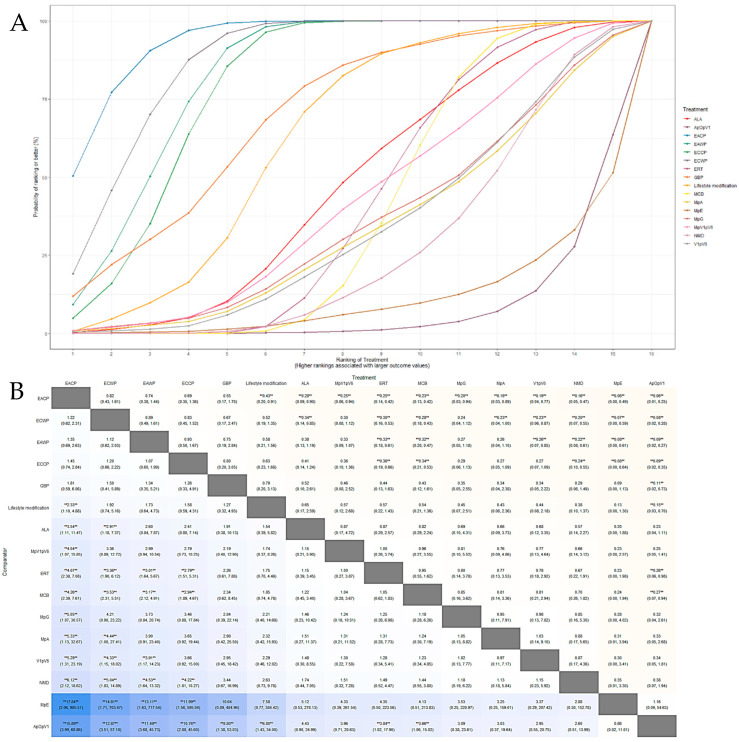
(**A**) SUCRA plot for response rate; SUCRA: surface under the curve cumulative ranking probabilities, shows probability of ranking for each treatment illustrated by graphs. (**B**) League heat plot for response rate; OR is statistically significant when the 95% credible interval does not include 1 and are indicated by a double asterisk in league heat plot.

The SUCRA plot for the response rate with MCB as a comparator is shown in Figure 6A. The highest-ranked treatments were EACP (SUCRA = 0.945), EAWP (SUCRA = 0.831), ECWP (SUCRA = 0.875), ECCP (SUCRA = 0.800), and GBP (SUCRA = 0.72). The heat map in Figure 6B shows similar trends for all comparisons. EACP showed significantly better results than lifestyle modification (OR 2.32; 95% CrI 1.09 to 4.94), ALA (OR 3.69; 95% CrI 1.21 to 12.09), ERT (OR 4.08; 95% CrI 2.38 to 7.15), MpV1pV6 (OR 4.21; 95% CrI 1.12 to 17.99), MCB (OR 4.34; 95% CrI 2.52 to 7.83), V1pV6 (OR 5.23; 95% CrI 1.22 to 22.02), MpA (OR 5.20; 95% CrI 1.17 to 32.78), MpG (OR 5.39; 95% CrI 1.17 to 31.05), NMD (OR 6.30; 95% CrI 2.12 to 20.10), MpE (OR 17.34; 95% CrI 2.10 to 356.64), and ApOpV1 (OR 4.28; 95% CrI 1.09 to 71.43). These results show that the EACP is superior to all other interventions and has significantly different effects from those of most treatments.

#### 3.5.2. Motor Nerve Outcomes: MMNCV, PMNCV, UMNCV, TMNCV

**Figure 7:**
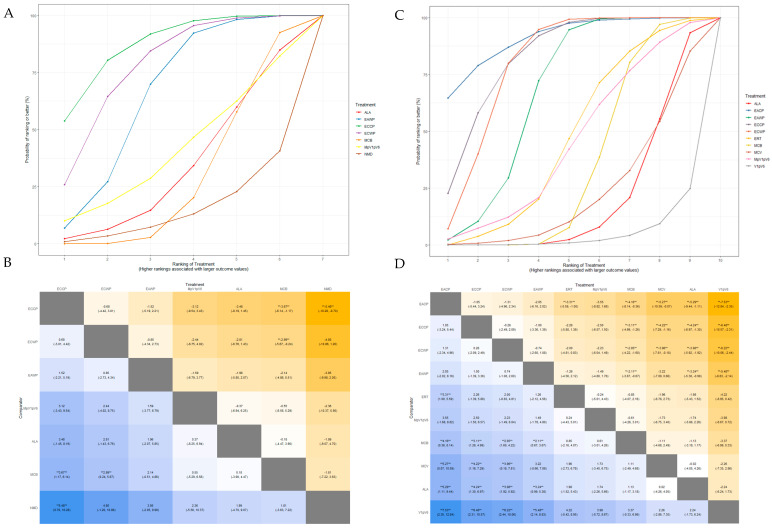
(**A**) SUCRA plot for MMNCV. (**B**) League heat plot for MMNCV. (**C**) SUCRA plot for PMNCV. (**D**) League heat plot for PMNCV. MD is statistically significant when the 95% credible interval does not include 0 and are indicated by a double asterisk in league heat plot.

The SUCRA plot for the MMNCV with MCB as a comparator is shown in Figure 7A. The highest-ranked treatments were ECCP (SUCRA = 0.872), ECWP (SUCRA = 0.783), and EAWP (SUCRA = 0.656). The heat map in Figure 7B shows similar trends for all comparisons. ECCP showed significantly better results than MCB (MD 3.67; 95% CrI 1.17 to 6.14) and NMD (MD 5.48; 95% CrI 0.70 to 10.28). ECCP was the best-ranked intervention in the network.

The SUCRA plot for the PMNCV with MCB as a comparator is shown in Figure 7C. The highest-ranked treatments were EACP (SUCRA = 0.915), ECCP (SUCRA = 0.834), ECWP (SUCRA = 0.803), and EAWP (SUCRA = 0.675). The heat map in Figure 7D shows similar trends for all comparisons EACP showed significantly better results than ERT (MD 3.31; 95% CrI, 1.00 to 5.59), MCB (MD 4.16; 95% CrI 0.36 to 8.14), MCV (MD 5.27; 95% CrI 0.07 to 10.59), ALA (MD 5.29; 95% CrI 1.11 to 9.44), and V1pV6 (MD 7.53; 95% CrI 2.32 to 12.84). EACP was the most effective intervention in the network. 

The SUCRA plot for TMNCV with MCB as the comparator is shown in Supplementary Figure S9. The highest-ranked treatments were ECWP (SUCRA = 0.728), EAWP (SUCRA = 0.727), and ECCP (SUCRA = 0.585). The heat map in Supplementary Figure S10 shows similar trends for all comparisons. ECWP showed remarkably better results than MCB (MD 3.89; 95% CrI 0.02 to 7.80). The ECWP was the best-ranked intervention in the network. 

The SUCRA plot for UMNCV with MCB as the comparator is shown in Supplementary Figure S11. The highest-ranked treatments were EAWP (SUCRA = 0.610), EACP (SUCRA = 0.601), ECCP (SUCRA = 0.572), and ALA (SUCRA = 0.503). The heat map in Supplementary Figure S12 shows similar trends for all comparisons. The EAWP was the best-ranked intervention in the network, but the difference was not statistically significant.

#### 3.5.3. Sensory Nerve Outcomes: MSNCV, PSNCV, USNCV, TSNCV

**Figure 8:**
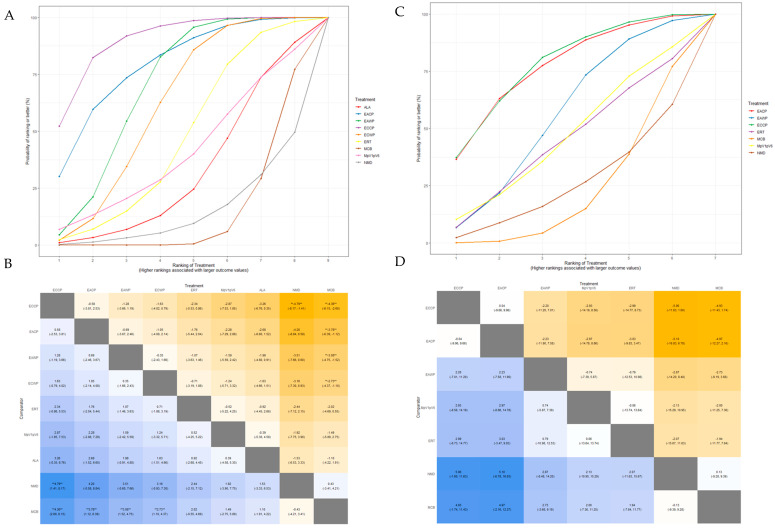
(**A**) SUCRA plot for MSNCV. (**B**) League heat plot for MSNCV. (**C**) SUCRA plot for USNCV. (**D**) League heatplot for USNCV. MD is statistically significant when the 95% credible interval does not include 0 and are indicated by a double asterisk in league heat plot.

The SUCRA plot for MSNCV with MCB as the comparator is shown in Figure 8A. The highest-ranked treatments were ECCP (SUCRA = 0.903), EACP (SUCRA = 0.794), EAWP (SUCRA = 0.697), and ECWP (SUCRA = 0.618). The heat map in Figure 8B shows similar trends for all comparisons. ECCP showed significantly better results than NMD (MD 4.79; 95% CrI 1.41 to 8.17), and NMD (MD 4.36; 95% CrI 2.60 to 6.15). ECCP was the best-ranked intervention in the network.

The SUCRA plot for PSNCV, with MCB as the comparator, is shown in Supplementary Figure S13. The highest-ranked treatments were EAWP (SUCRA = 0.915), ECCP (SUCRA = 0.740), EACP (SUCRA = 0.675), and V1pV6 (SUCRA = 0.672). The heat map in Supplementary Figure S14 shows similar trends for all comparisons. EAWP showed significantly better results than ECWP (MD 2.10; 95% CrI 0.19 to 4.05), MCB (MD 4.05; 95% CrI 2.46 to 5.73), ERT (MD 4.25; 95% CrI, 2.04 to 6.43), and ALA (MD 4.38; 95% CrI 2.20 to 6.51). The EAWP was the best-ranked intervention in the network. 

The SUCRA plot for TSNCV, with MCB as the comparator, is shown in Supplementary Figure S15. The highest-ranked treatments were EAWP (SUCRA = 0.855), ECCP (SUCRA = 0.740), EACP (SUCRA = 0.675), MpV1pV6 (SUCRA = 0.676), and V1pV6 (SUCRA = 0.671). The heat map in Supplementary Figure S16 shows similar trends for all comparisons. ECWP showed significantly better results than MCB (MD 3.55; 95% CrI 2.26 to 5.09), ALA (MD 5.16; 95% CrI 1.25 to 9.21), and NMD (MD 5.84; 95% CrI 1.93 to 10.00). The ECWP was the best-ranked intervention in the network.

The SUCRA plot for USNCV with MCB as the comparator is shown in Figure 8C. The highest-ranked treatments were ECCP (SUCRA = 0.785), EACP (SUCRA = 0.771), and EAWP (SUCRA = 0.552). The heat map in Figure 8D shows similar trends for all comparisons. ECCP was the best-ranked intervention in the network but was not statistically significant.

#### 3.5.4. Inconsistency Test

Regarding the response rate, as a result of node-splitting analysis of six interventions including multiple studies, no significant heterogeneity was observed in any comparison (EACP vs. ERT, *p* = 0.9615; EACP vs. MCB, *p* = 0.9997; EAWP vs. ERT, *p* = 0.7399; EAWP vs. MCV, *p* = 0.6651; ECCP vs. ERT, *p* = 0.9645; ECCP vs. MCV, *p* = 0.9968; ECWP vs. ERT, *p* = 0.6720; ECWP vs. MCV, *p* = 0.7770). Additionally, for all studies related to response rate, no finding supporting heterogeneity was confirmed in the comparison of the posterior mean deviance between the consistency and inconsistency models (Figure S17). In the case of secondary outcomes, DIC was compared using a leverage plot, and no significant inconsistency model DIC values were observed for any outcome that violated the consistency assumption (Figure S18A–H). 

### 3.6. Analysis of the Mechanism of the ACP on DPN through Network Pharmacology

#### 3.6.1. Active Ingredients and Anti-DPN Gene Targets of the ACP 

**Figure 9:**
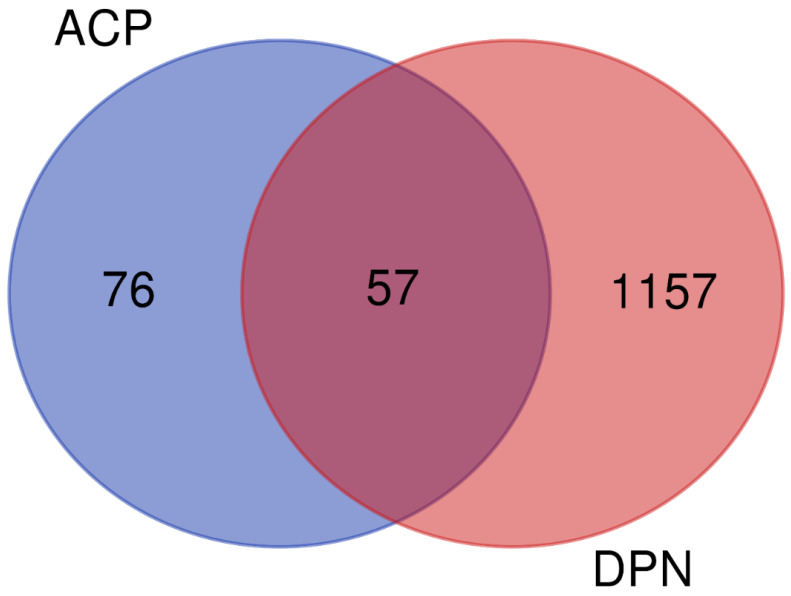
Venn diagram of targets of the ACP against DPN.

The TCMSP platform was screened using the absorption, distribution, metabolism, and excretion (ADME) criterion index of OB ≥ 30% and DL ≥ 0.18 to identify the active components in the ACP. A total of 27 active ingredients derived from the ACP were identified. Of these, 20 compounds occurred in Astragali Radix, and seven occurred in Cinnamomi Ramulus (Table 5). The DrugBank database contains information on 364 component–target relationships (Supplementary Table S4), and the GeneCards database contains information on 1157 human target genes associated with DPN (Supplementary Table S5). After intersection mapping, 57 consensus genes were identified as potential therapeutic targets of the ACP against DPN (Figure 9). 

#### 3.6.2. Network Analysis of the ACP and DPN Targets

**Figure 10:**
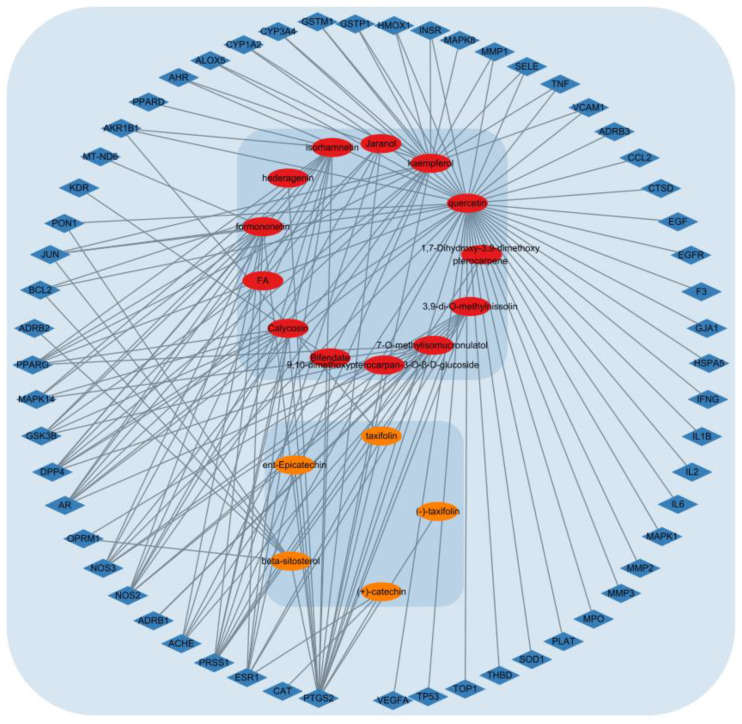
ACP–DPN network graph. Red nodes are active components of Astragali Radix; orange nodes are active components of Cinnamomi Ramulus; Blue nodes are potential multiple targets of ACP for the treatment of DPN.

The ACP component–DPN target network was mapped using Cytoscape software version 3.9.1. As shown in Figure 10, the network contained 78 nodes and 148 edges. The degree of a single target in the ACP–DPN network indicates the number of linked nodes. Network tools were analyzed to examine the network, and the degree of the active component was rated. Table 6 lists the top ten active ingredients according to degree, betweenness, and closeness centralities.

#### 3.6.3. PPI Network Construction

**Figure 11:**
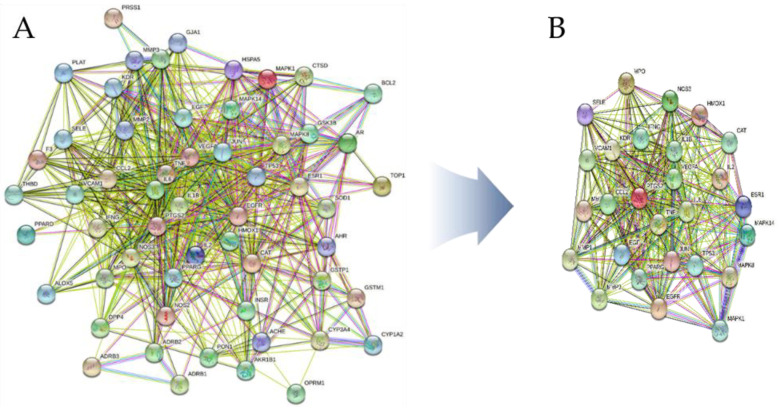
(**A**) ACP–DPN PPI network. (**B**) Top 27 hub targets ranked by degree centrality.

Using the STRING 11.5 platform, we imported the common targets and constructed a PPI network, as shown in Figure 11A. One target (MT-ND6) was excluded from the PPI network as it did not interact with any other target. The PPI network of intersecting targets contained 56 nodes and 612 edges. Nodes that satisfied the average value of degree centrality (21.47) were retrieved through an additional examination of topological attributes, and 30 targets were eliminated during screening. Figure 11B shows the PPI network of the hub targets. Table 7 lists the top 27 hub targets based on their degree of centrality. On the other hand, every PPI pair analyzed on the STRING platform is assigned a score. This score does not indicate the strength or specificity of the PPI, but rather its reliability based on the available evidence. Calculated on a scale of 0 to 1, the closer the score is to 1, the more likely it is that the PPI is true. The interaction scores for all PPI pairs utilized in the study are presented in Table S6.

#### 3.6.4. Gene Ontology and KEGG Pathway Enrichment Analysis

**Figure 12:**
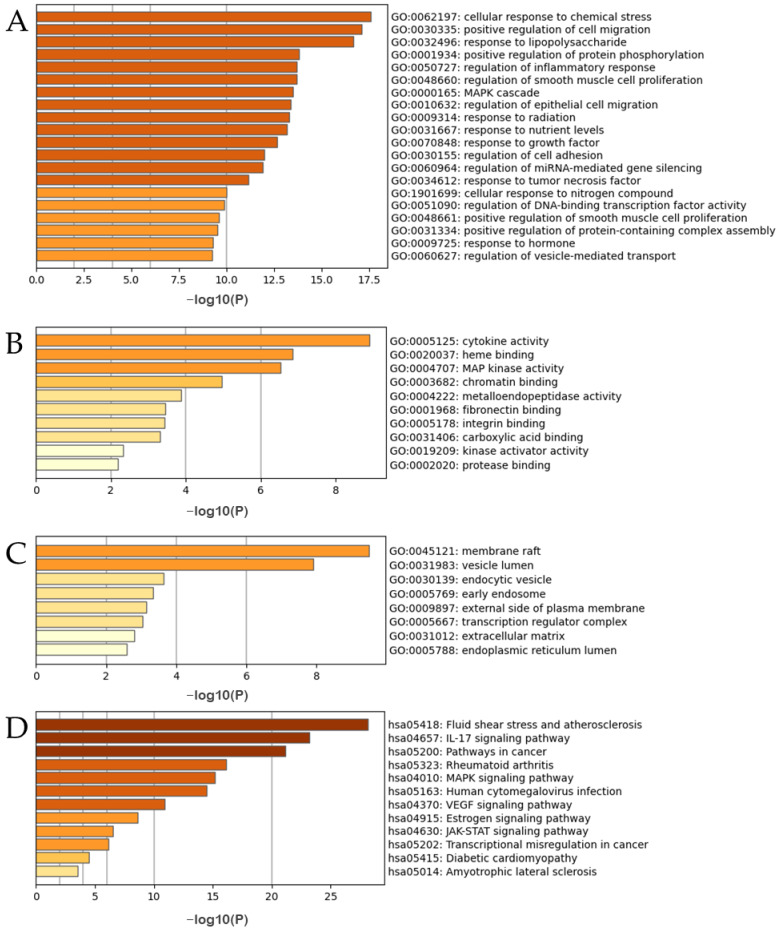
(**A**–**C**) GO enrichment analyses of DPN hub targets. (**D**) KEGG analysis of the DPN hub targets.

The results of the GO and KEGG analyses of the top 27 hub targets are shown in Figure 12. A total of 510 biological processes (BP) were identified, including the cellular response to chemical stress, positive regulation of cell migration, response to lipopolysaccharide, positive regulation of protein phosphorylation, regulation of inflammatory response, regulation of smooth muscle cell proliferation, the mitogen-activated protein kinase signaling (MAPK) cascade, and regulation of epithelial cell migration (Figure 12A). A total of 93 molecular functions were identified including cytokine activity, heme binding, MAP kinase activity, chromatin binding, metalloendopeptidase activity, fibronectin binding, integrin binding, carboxylic acid binding, kinase activator activity, and protease binding (Figure 12B). A total of 33 cellular components were identified, including the membrane raft, vesicle lumen, endocytic vesicle, early endosome, external side of the plasma membrane, transcription regulator complex, extracellular matrix, and endoplasmic reticulum lumen (Figure 12C). A total of 135 pathways were identified using KEGG pathway analysis (Figure 12D). The results suggested that the mechanisms of the ACP were mainly linked to fluid shear stress, atherosclerosis, and the interleukin-17 (IL-17), MAPK, and vascular endothelial growth factor (VEGF) signaling pathways (Table S7).

## 4. Discussion

### 4.1. Summary of the Findings

In our study, EAHM interventions were classified into four categories depending on the inclusion of the ACP and combination therapy with CM, and the comprehensive efficacy of EAHM interventions against DPN was compared with that of the CM control. EAHM showed considerably higher efficacy against DPN than the CM control, as determined by the response rate, SNCV, and MNCV indices, regardless of the mode of usage. The EAHM formula containing ACP was ranked highest in NMA for each treatment in terms of response rate, MMNCV, PMNCV, MSNCV, and USNCV. As a result, the ACP appears to be a candidate combination that can significantly influence the therapeutic response and nerve damage recovery in DPN. Based on network pharmacology analysis, the aforementioned study predicted that 10 compounds, including quercetin, kaempferol, isorhamnetin, formononetin, and beta-sitosterol, would act on 27 targets.

### 4.2. Strengths and Limitations

This study has the following strengths: First, there are countless meta-analyses related to EAHM; however, to the best of our knowledge, this is the first network meta-analysis to investigate the synergistic effect of an herb-pair. The analysis performed in this study is expected to be useful in identifying the synergistic effects of EAHM through continuous improvements and developments in the future. Second, the mechanism of EAHM was reviewed at a deeper level using network pharmacology analysis in conjunction with NMA in clinical studies. Because the mechanism of action of EAHM is complex, detailed pharmacological information is often not discussed in clinical studies. Therefore, this study is valuable because it supports the efficacy hypothesis for DPN to be tested in EAHM clinical research. Third, the overall direction of this study was consistent with the proposal for determining candidate combinations for drug discovery. Meta-analysis is one of the most important clinical research methodologies; however, in the case of EAHM, personalized prescription is advantageous, and it is difficult to draw a firm conclusion about which material is valid owing to the heterogeneity between different EAHM formulae. The authors suggest that meta-analysis may be a useful tool for developing new drug candidates by scientifically validating the tacit knowledge associated with complex EAHM combinations.

Due to the following limitations, caution should be exercised when interpreting the results of this study: First, although the EAHM formula containing the ACP at various NMA endpoints occupied the highest rank, the results were not consistent in terms of all indicators. This is mainly because the interactions with herbs other than the ACP also affect the efficacy, and few studies have performed a stable-effect comparison between multiple treatments. However, the design of this study was based on the premise that the ACP is a combination with appropriate compatibility, and its synergistic effect is stronger than that for other herbal combinations. To overcome these limitations, additional clinical trials are required to conduct updated NMA. Second, the quality of the studies included in the NMA was generally low, and no RCTs employed a double-blind design. This is another limitation that can affect the results. As a follow-up to this review, the validation of the effect of the ACP may be firmly established with new clinical trials with an improved design in the future. Third, in this study, the mechanism was analyzed using network pharmacology. However, as the compounds and targets of ACP have not yet been fully identified, database-based mechanism analysis based on data from previous studies revealed only predictive and not definitive mechanisms. Therefore, conclusions regarding the synergistic effects of the ACP and DPN can only be drawn through experimental studies. Prior to experimental testing, this study should be accepted to provide guidance.

### 4.3. Implications for Clinical Decision-Making

The significant difference between the effects of EAHM and the CM control, which was supported by the PMA data, is important because CM was used as a comparative treatment in most studies. Moreover, these results are encouraging because they are consistent with previous studies of similar design that investigated the effect of EAHM on DPN [31,32,33,38,107]. However, such a meta-analysis, which includes several types of EAHM formulae, has many limitations in its direct application to clinical decision-making, owing to strong heterogeneity due to differences in intervention composition and dose. Nevertheless, the consistent efficacy demonstrated by the findings of PMA in several previous studies and this review reinforces the idea that EAHM is a highly valuable candidate for drug discovery, at least for DPN treatment.

The EAHM formula containing the ACP occupied the highest rank among the multiple indicators included in the NMA target. Clinical evidence has established that EAHM formulae containing the ACP are useful for DPN, and the related mechanisms have been extensively explored [38,41,42,108]. Considering this and the fact that the ACP has long been used in combination with several EAHM prescriptions, the compatibility between the two components of the ACP is supported academically and historically. Moreover, both Astragali Radix and Cinnamomi Ramulus that make up the ACP have been shown to separately exert a wide range of pharmacological effects on systemic diseases, including the nervous, immune, endocrine, and cardiovascular systems, and are widely used medicinal plants [109,110,111,112]. Overall, EAHM formulae containing the ACP are considered superior for the treatment of DPN, and the development of a new drug for DPN using ACP or an EAHM combination containing the ACP as a candidate component seems valuable.

### 4.4. Implications for Drug Discovery

It is important to understand the herb-pair theory of EAHM outlined in the introduction to accurately predict the synergistic effects of herbal medicine combinations and apply it for drug discovery [22,24,25,26,113,114,115]. EAHM is often used as a polyherbal mixture following established academic principles. The synergistic effects of these mixtures are expected to improve their efficacy while lowering the potential toxicity of the individual herbs. This is made feasible by the basic prescription premise of EAHM, which is “Gun-Shin-Jwa-Sa” (King–Retainer–Officer–Messenger in English) [27]. The places of “Gun” and “Shin” are given to herbs that have the strongest influence and in greater doses. In contrast, relatively smaller doses of herbs are considered at “Jwa” and “Sa” to reduce adverse effects or boost synergistic effects. Thus, a suitable herbal combination can exhibit amplified efficacy compared to a single herb [30,116,117,118,119,120]. To establish these synergistic effects, an appropriate combination of EAHMs must be selected for drug development. Herb-pair theory is the most fundamental theory for compatibility [28,121,122]. This is extremely helpful as a research hypothesis for evaluating synergistic effects because it facilitates the development of an EAHM formula through the combination of two or three herbs.

Therefore, in recent years, an increasing number of studies have used various methods to identify the synergistic mechanisms of potentially useful herb-pairs [123,124,125]. A previous study using a combination of network pharmacology and bioinformatics reported that Astragali Radix, which was also used in this study, could form a promising herbal pair for the treatment of DPN with Notoginseng Radix [126,127]. We combined network meta-analysis and network pharmacology analyses with reference to the latest studies to investigate the clinical effects and synergistic mechanisms of the ACP simultaneously. We found that DPN treatment using an EAHM involving the ACP is closely related to the IL-17 signaling pathway. The IL-17 cytokine family is primarily associated with acute and chronic inflammation. Accordingly, this pathway is considered a therapeutic target for chronic inflammatory diseases in humans, and blocking this pathway prevents the onset of type 1 diabetes in rodent models [128,129]. In addition, a recent cross-sectional study confirmed that the development of peripheral neuropathy in patients with type 2 diabetes was independently and positively associated with elevated IL-17 levels. This study suggests that IL-17 may have greater diagnostic value for DPN than other inflammatory cytokines [130]. The ACP is also involved in the regulation of the MAPK pathway. This is important because the MAPK cascade is a major factor in DPN pathogenesis. Recent studies have shown that nerve growth factors induced by high blood glucose levels promote an increase in MAPK levels, which contributes to an increase in the levels of inflammatory mediators that cause DPN, such as tumor necrosis factor (TNF) and IL-1. Increased levels of MAPKs are also involved in the pathogenesis of DPN via inflammatory cytokines via the activation of c-Jun/JNK. Therefore, the inhibitory effect of the ACP on this mechanism is significant because activation of MAPKs contributes to the overall progression of DPN [131]. Additionally, neuroinflammation and neurodegeneration are important pathologies in diabetic complications including DPN. Hyperglycemia-induced reactive metabolites damage the blood vessels and promote capillary thickening and endothelial proliferation. The resulting decreased oxygen supply and increased reactive oxygen species (ROS) synthesis further damage the neurons and induce vascular endothelial growth factor (VEGF) expression. Therefore, VEGF has been extensively studied as a primary single-molecule target for the treatment of DPN, and our study predicted that the ACP could exert its therapeutic effect on DPN based on its action on this target [132]. Collectively, these potential predictive mechanisms and the fact that neuroinflammation is one of the major pathologies of DPN suggest that the ACP’s mechanism of action is likely related to the inhibition of inflammation-induced neuronal degeneration. [10]. In this regard, the ACP may have neuroprotective effects similar to those of berberine, which served as a target in a rat model of diabetic neuropathy, resulting in better neuritin expression and micropathology [133,134].

The results of our study showed that ten active components, according to degree centrality, ensured the main effects of the ACP. Among these compounds, quercetin, kaempferol, isorhamnetin, formononetin, and beta-sitosterol are thought to exert synergistic effects [135,136]. Quercetin is a promising candidate compound for a multitargeted approach to the complications of type 2 diabetes and has been reported to reduce oxidative stress, protect beta cells, and stimulate glucose uptake in muscle cells via the AMPK pathway [137]. In addition, it prevented diabetic complications by alleviating oxidative stress-induced apoptosis in a rat model of type 1 diabetes [138]. Kaempferol inhibits hyperglycemia-induced RhoA activation and diabetic kidney disease by reducing oxidative stress and proinflammatory cytokine levels [139]. Isorhamnetin is known for its various physiological activities, including neuroprotective, anti-inflammatory, antioxidative, and immunomodulatory effects [140,141]. DPN-related mechanisms have also been reported to prevent hyperglycemia by promoting glucose uptake by skeletal muscle cells and inhibiting insulin resistance [142,143]. Formononetin suppresses neuronal damage by controlling hyperglycemia in a rat model of diabetic neuropathy, improves nerve conduction velocity, and elicits synergistic effects by reducing thermal hyperalgesia and mechanical allodynia [144]. Finally, beta-sitosterol has been reported to have neuroprotective and antinociceptive effects in an animal model of diabetic neuropathic pain, based on insulin secretion promotion, alpha-glucosidase inhibition, blood sugar suppression, and antioxidant action [145]. In summary, several physiologically active ingredients present in the ACP may have synergistic effects on the prevention of nerve damage, repair of damaged nerves, and inhibition of DPN progression through an antidiabetic action via multiple pathways. This finding is consistent with the results of NMA in clinical trials. Therefore, the ACP is a promising candidate combination and its synergistic effects must be verified through subsequent experimental studies.

## 5. Conclusions

EAHM may promote therapeutic efficacy in the management of DPN; when combined with CM, it can treat DPN significantly more effectively than CM alone. EAHM formulae containing the ACP may be more suitable than other EAHM formulae for improving NCV and the treatment response rate to DPN therapy, as determined by a network meta-analysis. The ACP has also been shown to cure DPN synergistically via multiple pathways and may work primarily through the IL-17 and MAPK signaling pathways to alter the pathophysiology of chronic diabetes mellitus in peripheral nerves. These observations suggest that ACP could be used to treat pain, paresthesia, neuroinflammation, and neurodegeneration in patients with DPN.

The key ingredients in the ACP, including quercetin, kaempferol, isorhamnetin, formononetin, and beta-sitosterol, might have synergistic effects in neuroprotection, anti-inflammation, antioxidation, immunomodulation, and antinociception. These results suggest that ACP is a useful candidate for the treatment of DPN and should be studied further. However, the findings of this study should be verified through clinical trials and experimental studies. Despite these limitations, this study is valuable for proposing research hypotheses for candidate natural therapeutics based on herbal pairs and their synergistic effects.

Finally, the above study exploring the synergistic effects of herbal medicine combinations is expected to verify the efficacy of licensed herbal medicines and classical EAHM prescriptions consisting of multiple herbs based on a traditional holistic perspective. Currently, the standardization of herbal formulations mainly depends on the content of a single indicator component; however, this information is insufficient to support efficacy. Therefore, an advanced test method to clarify the multilayered indications of herbal medicines is essential. To clarify the indications for complex herbal medicine prescriptions beyond the mechanisms and effects of individual herbal medicines, it is necessary to continuously and multidimensionally verify the synergistic effects explored in this study.

## Figures and Tables

**Table 2:** Methodological quality of the included studies according to the Risk of Bias 2.0 tool.

Included Study	D1	D2	D3	D4	D5	Overall
Jin 2004 [59]	Sc	H	H	L	Sc	H
Sun 2008 [60]	Sc	H	H	Sc	Sc	H
Shen 2009 [61]	L	H	H	L	Sc	H
Lin 2010 [62]	Sc	H	H	L	Sc	H
Wang 2010 [63]	L	H	H	L	Sc	H
Yan 2010 [64]	Sc	H	H	Sc	Sc	H
Wu 2011 [65]	Sc	H	H	L	Sc	H
Gao 2012 [66]	Sc	H	H	L	Sc	H
Gong 2013 [67]	Sc	H	H	L	Sc	H
Han 2013 [68]	L	H	H	Sc	Sc	H
Zhang 2013a [69]	L	H	H	L	Sc	H
Zhang 2013b [70]	Sc	H	H	L	Sc	H
Guo 2014 [71]	Sc	H	H	Sc	Sc	H
Yang 2014a [73]	Sc	H	H	Sc	Sc	H
Yang 2014b [72]	L	H	H	Sc	Sc	H
Qi 2015 [74]	Sc	H	H	L	Sc	H
Wang 2015 [75]	Sc	H	H	Sc	Sc	H
Xue 2015 [76]	L	H	H	L	Sc	H
Guo 2016 [77]	L	H	H	L	Sc	H
Han 2016 [78]	Sc	H	H	L	Sc	H
Lan 2016 [79]	H	H	H	L	Sc	H
Mo 2016 [82]	L	H	H	Sc	Sc	H
Wang 2016 [83]	L	H	H	Sc	Sc	H
Li 2016a [80]	L	H	H	L	Sc	H
Zhang 2016a [85]	L	H	H	L	Sc	H
Li 2016b [81]	Sc	H	H	L	Sc	H
Zhang 2016b [84]	L	H	H	L	Sc	H
Chen 2017 [86]	L	H	H	L	Sc	H
Shi 2017 [87]	Sc	H	H	L	Sc	H
Wang 2017 [88]	L	H	H	L	Sc	H
Chen 2018 [89]	L	H	H	Sc	Sc	H
Dai 2018 [90]	H	H	H	L	Sc	H
Hu 2018 [92]	Sc	H	H	L	Sc	H
Huang 2018 [93]	L	H	H	L	Sc	H
She 2018 [94]	L	H	H	L	Sc	H
Xin 2018 [95]	H	H	H	Sc	Sc	H
Gao 2019 [91]	Sc	H	H	L	Sc	H
Wu 2019 [99]	L	H	H	L	Sc	H
Yi 2019 [100]	L	H	H	L	Sc	H
Ji 2019 [96]	L	H	H	Sc	Sc	H
Liu 2019a [97]	Sc	H	H	L	Sc	H
Liu 2019b [98]	Sc	H	H	Sc	Sc	H
Chen 2021 [101]	Sc	H	Sc	L	Sc	H
Hou 2021 [102]	Sc	H	H	L	Sc	H
Li 2021 [103]	L	H	H	L	Sc	H
Wang 2021a [105]	L	H	H	L	Sc	H
Wang 2021b [104]	L	H	H	L	Sc	H
Zhang 2021 [106]	H	H	H	L	Sc	H

D1–D5: five domain criteria; D1: bias arising from the randomization process; D2: bias due to deviations from intended interventions; D3: bias due to missing outcome data; D4: bias in the measurement of the outcome; D5: bias in the selection of the reported results; H: high risk of bias; L: low risk of bias; Sc: some concerns.

**Table 3:** Quality of evidence ratings for the response rate in pairwise meta-analysis.

Intervention and Comparator Intervention	Outcomes	Number of Participants (Studies)	Anticipated Absolute Effects (95% CI)	Quality of the Evidence (GRADE)
EACP compared to CM for diabetic peripheral neuropathy	Response rate	516 (7)	224 more per 1000 (from 139 more to 318 more)	⨁⨁⨁◯ MODERATE a
ECCP compared to CM for diabetic peripheral neuropathy	Response rate	1006 (11)	134 more per 1000 (from 77 more to 196 more)	⨁⨁◯◯ LOW a,c
EAWP compared to CM for diabetic peripheral neuropathy	Response rate	931 (11)	184 more per 1000 (from 96 more to 283 more)	⨁⨁◯◯ LOW a,c
ECWP compared to CM for diabetic peripheral neuropathy	Response rate	908 (12)	190 more per 1000 (from 130 more to 255 more)	⨁⨁◯◯ LOW a,c

CM: conventional medicine; EACP: East Asian herbal medicine monotherapy containing the Astragali Radix–Cinnamomi Ramulus herb-pair; EAWP: East Asian herbal medicine monotherapy without the Astragali Radix–Cinnamomi Ramulus herb-pair; ECCP: East Asian herbal medicine and conventional medicine combined therapy containing the Astragali Radix–Cinnamomi Ramulus herb-pair; ECWP: East Asian herbal medicine and conventional medicine combined therapy without the Astragali Radix–Cinnamomi Ramulus herb-pair. GRADE working group grades of evidence. High quality(⨁⨁⨁⨁): further research is unlikely to change our confidence in estimating this effect. Moderate quality(⨁⨁⨁◯): further research is likely to have an important impact on our confidence in estimating the effect, and may change the estimate. Low quality(⨁⨁◯◯): further research is likely to impact our confidence in the estimate of the effect and is likely to change the estimate. Very low quality(⨁◯◯◯): very uncertain about the estimate. a: study design with some bias in randomized or distributed blind. b: 95% confidence interval passes 0 (MD and SMD) or 1 (RR and OR), and other interventions are not satisfied. c: confidence intervals are less overlapping, or the heterogeneity is high.

**Table 4:** Network characteristics of each outcome included in the NMA.

Characteristic	Response Rate	Motor Nerve Outcomes				Sensory Nerve Outcomes			
		MMNCV	PMNCV	TMNCV	UMNCV	MSNCV	PSNCV	TSNCV	USNCV
Number of interventions	16	7	10	5	7	9	10	9	7
Number of included trials	42	14	21	9	4	20	21	13	6
Total number of patients in network	3588	1365	2092	866	360	1894	2092	1348	523
Total possible pairwise comparisons	120	21	45	10	5	36	45	36	21
Total number of pairwise comparisons with direct data	18	7	10	4	4	10	10	8	6
Is the network connected?	TRUE	TRUE	TRUE	TRUE	FALSE	TRUE	TRUE	TRUE	TRUE
Number of two-arm studies	42	14	21	9	4	20	21	13	6
Number of multiple arm studies	0	0	0	0	0	0	0	0	0
Average outcome (for continuous variables)	NA	49.34	42.44	43.07	46.76	44.37	42.44	39.83	42.38
Total number of events in network (for dichotomous variables)	2777	NA	NA	NA	NA	NA	NA	NA	NA
Number of studies with at least one zero events (for dichotomous variables)	42	NA	NA	NA	NA	NA	NA	NA	NA
Number of studies with all zero events (for dichotomous variables)	0	NA	NA	NA	NA	NA	NA	NA	NA

**Table 5:** Detailed information on the active compounds in the ACP.

Mol ID	Mol Name	Structure	OB (%)	DL
MOL000359	sitosterol	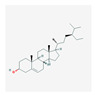	36.91	0.75
MOL000358	beta-sitosterol	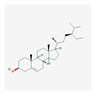	36.91	0.75
MOL011169	Peroxyergosterol	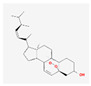	44.39	0.82
MOL000073	ent-Epicatechin	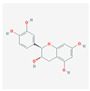	48.96	0.24
MOL000492	(+)-catechin	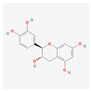	54.83	0.24
MOL004576	taxifolin	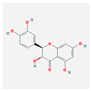	57.84	0.27
MOL001736	(−)-taxifolin	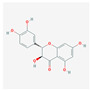	60.51	0.27
MOL000387	Bifendate	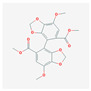	31.10	0.67
MOL000033	(3S,8S,9S,10R,13R,14S,17R)-10,13-dimethyl-17-[(2R,5S)-5-propan-2-yloctan-2-yl]-2,3,4,7,8,9,11,12,14,15,16,17-dodecahydro-1H-cyclopenta[a]phenanthren-3-ol	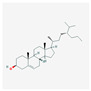	36.23	0.78
MOL000379	9,10-dimethoxypterocarpan-3-O-β-D-glucoside	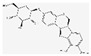	36.74	0.92
MOL000296	hederagenin	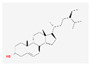	36.91	0.75
MOL000442	1,7-Dihydroxy-3,9-dimethoxy pterocarpene	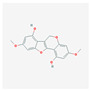	39.05	0.48
MOL000374	5′-hydroxyiso-muronulatol-2′,5′-di-O-glucoside	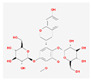	41.72	0.69
MOL000422	kaempferol	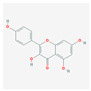	41.88	0.24
MOL000098	quercetin	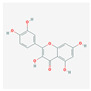	46.43	0.28
MOL000417	Calycosin	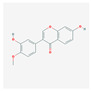	47.75	0.24
MOL000439	isomucronulatol-7,2′-di-O-glucosiole	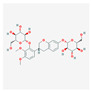	49.28	0.62
MOL000354	isorhamnetin	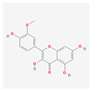	49.60	0.31
MOL000239	Jaranol	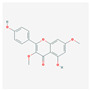	50.83	0.29
MOL000371	3,9-di-O-methylnissolin	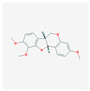	53.74	0.48
MOL000211	Mairin	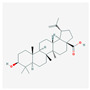	55.38	0.78
MOL000380	(6aR,11aR)-9,10-dimethoxy-6a,11a-dihydro-6H-benzofurano [3,2-c]chromen-3-ol	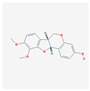	64.26	0.42
MOL000438	(3R)-3-(2-hydroxy-3,4-dimethoxyphenyl)chroman-7-ol	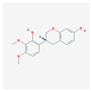	67.67	0.26
MOL000433	FA	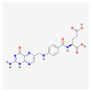	68.96	0.71
MOL000392	formononetin	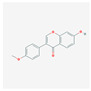	69.67	0.21
MOL000378	7-O-methylisomucronulatol	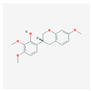	74.69	0.30
MOL000398	isoflavanone	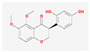	109.99	0.30

Mol ID: molecule ID; Mol name: molecule name; OB: oral bioavailability; DL: drug-likeness.

**Table 6:** The top ten active compounds of the ACP.

Molecule Name	Degree Centrality	Betweenness Centrality	Closeness Centrality	Included Herb
quercetin	45	0.636	0.632	AR
kaempferol	23	0.140	0.460	AR
isorhamnetin	13	0.067	0.413	AR
formononetin	13	0.066	0.413	AR
7-O-methylisomucronulatol	11	0.042	0.404	AR
Calycosin	9	0.022	0.396	AR
3,9-di-O-methylnissolin	8	0.034	0.387	AR
beta-sitosterol	6	0.044	0.379	CR
Jaranol	5	0.003	0.376	AR
(+)-catechin	3	0.028	0.368	CR

AR: Astragali Radix; CR: Cinnamomi Ramulus.

**Table 7:** The top 27 hub targets.

Number	Gene Name	Degree Centrality	Betweenness Centrality	Closeness Centrality
1	*TNF*	45	0.054	0.846
2	*IL6*	44	0.047	0.833
3	*VEGFA*	43	0.041	0.821
4	*TP53*	41	0.042	0.797
5	*IL1B*	40	0.028	0.786
6	*PTGS2*	38	0.025	0.764
7	*CAT*	37	0.046	0.753
8	*EGFR*	37	0.024	0.753
9	*JUN*	37	0.032	0.743
10	*NOS3*	36	0.047	0.733
11	*PPARG*	36	0.037	0.733
12	*EGF*	35	0.014	0.733
13	*CCL2*	34	0.013	0.724
14	*ESR1*	33	0.033	0.714
15	*HMOX1*	32	0.010	0.705
16	*MMP2*	32	0.014	0.705
17	*VCAM1*	30	0.009	0.688
18	*IFNG*	28	0.019	0.671
19	*IL2*	28	0.004	0.671
20	*MPO*	26	0.008	0.655
21	*MAPK8*	25	0.008	0.647
22	*SELE*	25	0.003	0.640
23	*MAPK14*	24	0.002	0.632
24	*MMP3*	24	0.022	0.632
25	*MAPK1*	23	0.008	0.632
26	*KDR*	22	0.018	0.611
27	*MMP1*	22	0.002	0.625

## Data Availability

The data in this study were all utilized from public databases and published studies and had unrestricted access.

## Supplementary Materials

The following supporting information can be downloaded at: https://www.mdpi.com/article/10.3390/pharmaceutics15051361/s1, Supplementary Table S1. Search strategies; Supplementary Table S2. EAHM ingredients used in clinical trials included in this review; Supplementary Table S3. Quality of evidence ratings for secondary outcomes in pairwise meta-analysis; Supplementary Table S4. The protein targets from the candidate bioactive components in ACP; Supplementary Table S5. The detailed information of potential DPN targets from GeneCard database; Supplementary Table S6. Protein–protein interaction score; Supplementary Table S7. KEGG enrichment analysis; Supplementary Figure S1. Forest plot of the trials that compared EAHM with CM for MMNCV; Supplementary Figure S2. Forest plot of the trials that compared EAHM with CM for PMNCV; Supplementary Figure S3. Forest plot of the trials that compared EAHM with CM for UMNCV; Supplementary Figure S4. Forest plot of the trials that compared EAHM with CM for TMNCV; Supplementary Figure S5. Forest plot of the trials that compared EAHM with CM for MSNCV; Supplementary Figure S6. Forest plot of the trials that compared EAHM with CM for PSNCV; Supplementary Figure S7. Forest plot of the trials that compared EAHM with CM for USNCV; Supplementary Figure S8. Forest plot of the trials that compared EAHM with CM for TSNCV; Supplementary Figure S9. SUCRA plot for TMNCV; Supplementary Figure S10. League heat plot for TMNCV; Supplementary Figure S11. SUCRA plot for UMNCV; Supplementary Figure S12. League heat plot for UMNCV; Supplementary Figure S13. SUCRA plot for PSNCV; Supplementary Figure S14. League heat plot for PSNCV; Supplementary Figure S15. SUCRA plot for TSNCV; Supplementary Figure S16. League heat plot for TSNCV; Supplementary Figure S17. Posterior mean deviance compare plot for response rate; Supplementary Figure S18A. Leverage plots and fit statistics for MMNCV; (B) Leverage plots and fit statistics for PMNCV; (C) Leverage plots and fit statistics for TMNCV; (D) Leverage plots and fit statistics for UMNCV; (E) Leverage plots and fit statistics for MSNCV; (F) Leverage plots and fit statistics for PSNCV; (G) Leverage plots and fit statistics for TSNCV; (H) Leverage plots and fit statistics for USNCV.

## References

[1] Selvarajah D., Kar D., Khunti K., Davies M.J., Scott A.R., Walker J., Tesfaye S. (2019). Diabetic Peripheral Neuropathy: Advances in Diagnosis and Strategies for Screening and Early Intervention. Lancet Diabetes Endocrinol..

[2] Saeedi P., Petersohn I., Salpea P., Malanda B., Karuranga S., Unwin N., Colagiuri S., Guariguata L., Motala A.A., Ogurtsova K. (2019). Global and Regional Diabetes Prevalence Estimates for 2019 and Projections for 2030 and 2045: Results from the International Diabetes Federation Diabetes Atlas, 9th Edition. Diabetes Res. Clin. Pract..

[3] Jensen T.S., Karlsson P., Gylfadottir S.S., Andersen S.T., Bennett D.L., Tankisi H., Finnerup N.B., Terkelsen A.J., Khan K., Themistocleous A.C. (2021). Painful and Non-Painful Diabetic Neuropathy, Diagnostic Challenges and Implications for Future Management. Brain J. Neurol..

[4] Gonçalves N.P., Vægter C.B., Andersen H., Østergaard L., Calcutt N.A., Jensen T.S. (2017). Schwann Cell Interactions with Axons and Microvessels in Diabetic Neuropathy. Nat. Rev. Neurol..

[5] Pang L., Lian X., Liu H., Zhang Y., Li Q., Cai Y., Ma H., Yu X. (2020). Understanding Diabetic Neuropathy: Focus on Oxidative Stress. Oxid. Med. Cell Longev..

[6] Calcutt N.A. (2020). Diabetic Neuropathy and Neuropathic Pain: A (Con)Fusion of Pathogenic Mechanisms?. Pain.

[7] Kiyani M., Yang Z., Charalambous L.T., Adil S.M., Lee H.-J., Yang S., Pagadala P., Parente B., Spratt S.E., Lad S.P. (2020). Painful Diabetic Peripheral Neuropathy: Health Care Costs and Complications from 2010 to 2015. Neurol. Clin. Pract..

[8] Waldfogel J.M., Nesbit S.A., Dy S.M., Sharma R., Zhang A., Wilson L.M., Bennett W.L., Yeh H.-C., Chelladurai Y., Feldman D. (2017). Pharmacotherapy for Diabetic Peripheral Neuropathy Pain and Quality of Life: A Systematic Review. Neurology.

[9] Asrar M.M., Kumari S., Sekhar B.C., Bhansali A., Bansal D. (2021). Relative Efficacy and Safety of Pharmacotherapeutic Interventions for Diabetic Peripheral Neuropathy: A Systematic Review and Bayesian Network Meta-Analysis. Pain Physician.

[10] Yang K., Wang Y., Li Y.-W., Chen Y.-G., Xing N., Lin H.-B., Zhou P., Yu X.-P. (2022). Progress in the Treatment of Diabetic Peripheral Neuropathy. Biomed. Pharmacother. Biomed. Pharmacother..

[11] Huang D.-D., Shi G., Jiang Y., Yao C., Zhu C. (2020). A Review on the Potential of Resveratrol in Prevention and Therapy of Diabetes and Diabetic Complications. Biomed. Pharmacother..

[12] Addepalli V., Suryavanshi S.V. (2018). Catechin Attenuates Diabetic Autonomic Neuropathy in Streptozotocin Induced Diabetic Rats. Biomed. Pharmacother..

[13] Ebrahimi F., Farzaei M.H., Bahramsoltani R., Heydari M., Naderinia K., Rahimi R. (2019). Plant-Derived Medicines for Neuropathies: A Comprehensive Review of Clinical Evidence. Rev. Neurosci..

[14] Arora K., Tomar P.C., Mohan V. (2021). Diabetic Neuropathy: An Insight on the Transition from Synthetic Drugs to Herbal Therapies. J. Diabetes Metab. Disord..

[15] Bao X.-Y., Deng L.-H., Huang Z.-J., Daror A.S., Wang Z.-H., Jin W.-J., Zhuang Z., Tong Q., Zheng G.-Q., Wang Y. (2021). Buyang Huanwu Decoction Enhances Revascularization via Akt/GSK3*β*/NRF2 Pathway in Diabetic Hindlimb Ischemia. Oxid. Med. Cell Longev..

[16] Zhu C., Liu N., Tian M., Ma L., Yang J., Lan X., Ma H., Niu J., Yu J. (2020). Effects of Alkaloids on Peripheral Neuropathic Pain: A Review. Chin. Med..

[17] Singh R., Kaur N., Kishore L., Gupta G.K. (2013). Management of Diabetic Complications: A Chemical Constituents Based Approach. J. Ethnopharmacol..

[18] Lee J.Y., Choi H.Y., Park C.S., Pyo M.K., Yune T.Y., Kim G.W., Chung S.H. (2019). GS-KG9 Ameliorates Diabetic Neuropathic Pain Induced by Streptozotocin in Rats. J. Ginseng Res..

[19] Ji H.Y., Liu K.H., Kong T.Y., Jeong H.-U., Choi S.-Z., Son M., Cho Y.-Y., Lee H.S. (2013). Evaluation of DA-9801, a New Herbal Drug for Diabetic Neuropathy, on Metabolism-Mediated Interaction. Arch. Pharm. Res..

[20] Nam Y.H., Moon H.W., Lee Y.R., Kim E.Y., Rodriguez I., Jeong S.Y., Castañeda R., Park J.-H., Choung S.-Y., Hong B.N. (2019). Panax Ginseng (Korea Red Ginseng) Repairs Diabetic Sensorineural Damage through Promotion of the Nerve Growth Factor Pathway in Diabetic Zebrafish. J. Ginseng Res..

[21] Yang D., Liang X.-C. (2018). Strategies and Research Progress of Chinese Medicine in Prevention and Treatment of Diabetic Peripheral Neuropathy. Chin. J. Integr. Med..

[22] Yin C.S., Ko S.-G. (2014). Introduction to the History and Current Status of Evidence-Based Korean Medicine: A Unique Integrated System of Allopathic and Holistic Medicine. Evid.-Based Complement. Altern. Med. ECAM.

[23] Shim J.-M., Kim J. (2018). Cross-National Differences in the Holistic Use of Traditional East Asian Medicine in East Asia. Health Promot. Int..

[24] Shim J.-M., Lee Y.-S. (2017). The Association between the Use of Biomedical Services and the Holistic Use of Traditional East Asian Medicine: A National Survey of Outpatients in South Korea. BMJ Open.

[25] Cha W.-S., Oh J.-H., Park H.-J., Ahn S.-W., Hong S.-Y., Kim N.-I. (2007). Historical Difference between Traditional Korean Medicine and Traditional Chinese Medicine. Neurol. Res..

[26] Shim J.-M. (2016). The Relationship Between the Use of Complementary and Alternative Medicine and the Use of Biomedical Services: Evidence From East Asian Medical Systems. Asia. Pac. J. Public Health.

[27] Kim H.U., Ryu J.Y., Lee J.O., Lee S.Y. (2015). A Systems Approach to Traditional Oriental Medicine. Nat. Biotechnol..

[28] Wang S., Hu Y., Tan W., Wu X., Chen R., Cao J., Chen M., Wang Y. (2012). Compatibility Art of Traditional Chinese Medicine: From the Perspective of Herb Pairs. J. Ethnopharmacol..

[29] Yang Y., Zhang Z., Li S., Ye X., Li X., He K. (2014). Synergy Effects of Herb Extracts: Pharmacokinetics and Pharmacodynamic Basis. Fitoterapia.

[30] Zhou X., Seto S.W., Chang D., Kiat H., Razmovski-Naumovski V., Chan K., Bensoussan A. (2016). Synergistic Effects of Chinese Herbal Medicine: A Comprehensive Review of Methodology and Current Research. Front. Pharmacol..

[31] Xu H.-B., Jiang R.-H., Chen X.-Z., Li L. (2012). Chinese Herbal Medicine in Treatment of Diabetic Peripheral Neuropathy: A Systematic Review and Meta-Analysis. J. Ethnopharmacol..

[32] Hao C., Wu F., Lu L., Wang J., Guo Y., Liu A., Liao W., Zheng G. (2013). Chinese Herbal Medicine for Diabetic Peripheral Neuropathy: An Updated Meta-Analysis of 10 High-Quality Randomized Controlled Studies. PLoS ONE.

[33] Deng Z., Wang M., Fan Y.-H., Huang R., Rao Y., Mai X., Liu M. (2019). A Systematic Review of Randomized Controlled Trials of the Wenyang Huoxue Method in Treating Diabetic Peripheral Neuropathy. Medicine.

[34] Zhou W., Wang J., Wu Z., Huang C., Lu A., Wang Y. (2016). Systems Pharmacology Exploration of Botanic Drug Pairs Reveals the Mechanism for Treating Different Diseases. Sci. Rep..

[35] Zhao J., Li Y., Xin L., Sun M., Yu C., Shi G., Bao T., Liu J., Ni Y., Lu R. (2020). Clinical Features and Rules of Chinese Herbal Medicine in Diabetic Peripheral Neuropathy Patients. Evid.-Based Complement. Altern. Med. ECAM.

[36] Yue S.-J., Xin L.-T., Fan Y.-C., Li S.-J., Tang Y.-P., Duan J.-A., Guan H.-S., Wang C.-Y. (2017). Herb Pair Danggui-Honghua: Mechanisms Underlying Blood Stasis Syndrome by System Pharmacology Approach. Sci. Rep..

[37] Jeong S.J., Lim H.-S., Seo C.-S., Kim J.-H., Jin S.-E., Yoo S.-R., Shin H.-K., Jeong S.-J., Lim H.-S., Seo C.-S. (2015). Traditional Herbal Formula Jakyakgamcho-Tang (*Paeonia lactiflora* and *Glycyrrhiza uralensis*) Impairs Inflammatory Chemokine Production by Inhibiting Activation of STAT1 and NF-ΚB in HaCaT Cells. Phytomedicine.

[38] Pang B., Zhao T.-Y., Zhao L.-H., Wan F., Ye R., Zhou Q., Tian F., Tong X.-L. (2016). Huangqi Guizhi Wuwu Decoction for Treating Diabetic Peripheral Neuropathy: A Meta-Analysis of 16 Randomized Controlled Trials. Neural Regen. Res..

[39] Liang L., Wei X., Feng M., Zhu L., Yu J., Yang G., Yin X., Zhou S., Li K., Yang M. (2020). Huangqi Guizhi Wuwu Decoction for Treating Cervical Radiculopathy: A Systematic Review and Meta-Analysis of Randomized Controlled Trials. Medicine.

[40] Jo H.-G., Lee D. (2021). Oral Administration of East Asian Herbal Medicine for Peripheral Neuropathy: A Systematic Review and Meta-Analysis with Association Rule Analysis to Identify Core Herb Combinations. Pharmaceuticals.

[41] He Y., Zheng H., Zhong L., Zhong N., Wen G., Wang L., Zhang Y. (2021). Identification of Active Ingredients of Huangqi Guizhi Wuwu Decoction for Promoting Nerve Function Recovery After Ischemic Stroke Using HT22 Live-Cell-Based Affinity Chromatography Combined with HPLC-MS/MS. Drug Des. Devel. Ther..

[42] Lv Z., Shen J., Gao X., Ruan Y., Ling J., Sun R., Dai J., Fan H., Cheng X., Cao P. (2021). Herbal Formula Huangqi Guizhi Wuwu Decoction Attenuates Paclitaxel-Related Neurotoxicity via Inhibition of Inflammation and Oxidative Stress. Chin. Med..

[43] Zheng H.-Z., Shen X., He Y.-Y., Yan X.-L., Wang S.-X., Yu A.-M., Wang L.-S. (2020). Pharmacokinetic Analysis of Huangqi Guizhi Wuwu Decoction on Blood and Brain Tissue in Rats with Normal and Cerebral Ischemia-Reperfusion Injury by Microdialysis with HPLC-MS/MS. Drug Des. Devel. Ther..

[44] Liu W., Shi L., Wan Q., Wu Y., Huang D., Ou J., Liu Q., Guan X., Yang Y., Zhang X. (2021). Huangqi Guizhi Wuwu Decoction Attenuates Podocyte Cytoskeletal Protein Damage in IgA Nephropathy Rats by Regulating AT1R/Nephrin/c-Abl Pathway. Biomed. Pharmacother. Biomed. Pharmacother..

[45] Hutton B., Salanti G., Caldwell D.M., Chaimani A., Schmid C.H., Cameron C., Ioannidis J.P.A., Straus S., Thorlund K., Jansen J.P. (2015). The PRISMA Extension Statement for Reporting of Systematic Reviews Incorporating Network Meta-Analyses of Health Care Interventions: Checklist and Explanations. Ann. Intern. Med..

[46] Sterne J.A.C., Savović J., Page M.J., Elbers R.G., Blencowe N.S., Boutron I., Cates C.J., Cheng H.-Y., Corbett M.S., Eldridge S.M. (2019). RoB 2: A Revised Tool for Assessing Risk of Bias in Randomised Trials. BMJ.

[47] Lortie C.J., Filazzola A. (2020). A Contrast of Meta and Metafor Packages for Meta-Analyses in R. Ecol. Evol..

[48] Zhang Y., Akl E.A., Schünemann H.J. (2019). Using Systematic Reviews in Guideline Development: The GRADE Approach. Res. Synth. Methods.

[49] Egger M., Davey Smith G., Schneider M., Minder C. (1997). Bias in Meta-Analysis Detected by a Simple, Graphical Test. BMJ.

[50] Meng Z., Wu C., Lin L. (2023). The Effect Direction Should Be Taken into Account When Assessing Small-Study Effects. J. Evid.-Based Dent. Pract..

[51] Guyatt G.H., Oxman A.D., Vist G.E., Kunz R., Falck-Ytter Y., Alonso-Coello P., Schünemann H.J., GRADE Working Group (2008). GRADE: An Emerging Consensus on Rating Quality of Evidence and Strength of Recommendations. BMJ.

[52] Béliveau A., Boyne D.J., Slater J., Brenner D., Arora P. (2019). BUGSnet: An R Package to Facilitate the Conduct and Reporting of Bayesian Network Meta-Analyses. BMC Med. Res. Methodol..

[53] Xu C., Niu Y., Wu J., Gu H., Zhang C. (2018). Software and Package Applicating for Network Meta-Analysis: A Usage-Based Comparative Study. J. Evid.-Based Med..

[54] Ru J., Li P., Wang J., Zhou W., Li B., Huang C., Li P., Guo Z., Tao W., Yang Y. (2014). TCMSP: A Database of Systems Pharmacology for Drug Discovery from Herbal Medicines. J. Cheminform..

[55] Stelzer G., Rosen N., Plaschkes I., Zimmerman S., Twik M., Fishilevich S., Stein T.I., Nudel R., Lieder I., Mazor Y. (2016). The GeneCards Suite: From Gene Data Mining to Disease Genome Sequence Analyses. Curr. Protoc. Bioinforma..

[56] Szklarczyk D., Gable A.L., Nastou K.C., Lyon D., Kirsch R., Pyysalo S., Doncheva N.T., Legeay M., Fang T., Bork P. (2021). The STRING Database in 2021: Customizable Protein-Protein Networks, and Functional Characterization of User-Uploaded Gene/Measurement Sets. Nucleic Acids Res..

[57] Shannon P., Markiel A., Ozier O., Baliga N.S., Wang J.T., Ramage D., Amin N., Schwikowski B., Ideker T. (2003). Cytoscape: A Software Environment for Integrated Models of Biomolecular Interaction Networks. Genome Res..

[58] Zhou Y., Zhou B., Pache L., Chang M., Khodabakhshi A.H., Tanaseichuk O., Benner C., Chanda S.K. (2019). Metascape Provides a Biologist-Oriented Resource for the Analysis of Systems-Level Datasets. Nat. Commun..

[59] Jin J., Chen H., Zhao D., Feng Z., Liu Q., Zhang Z., Qin R. (2004). Clinical study on tangmaitong tablets in treating 103 cases of diabetic peripheral neuropathy. J. Tradit. Chin. Med..

[60] Sun L. (2008). Clinical Observation on Treatment of 30 Cases of Peripheral Neuroapthy of Type 2 Diabetes with Nourishing Yin Bushen Huoxue Tongluo Decoction. Chin. J. Med. Drug Appl..

[61] Shen J., Shu X. (2009). Clinical study of tangmaining capsule in the treatment of diabetic peripheral neuropathy. Chin. J. Exp. Tradit. Med..

[62] Lin M., Yu J., Deng Y., Tan S. (2010). Tongxinluo capsule combined with methyl vitamin B12 in the treatment of multiple diabetes observation of therapeutic effect of neuropathy. Chin. J. Clin. Ration. Drug Use.

[63] Wang Z., Wang J. (2010). Observation on curative effect of huangqi guizhi wuwu decoction in treating diabetic peripheral neuropathy. Qingdao Med. Health.

[64] Yan Q., Yu J. (2010). Evaluation of clinical therapeutic effect of the method for nourishing yin and activating blood circulation on diabetic peripheral neuropathy. Chin. Arch. Tradit. Chin. Med..

[65] Wu Y., Zhang J., Qin F. (2011). Modified yiqi huoxue decoction for the treatment of 30 cases of diabetic peripheral neuropathy. China Foreign Med. Treat..

[66] Gao Z., Wang X. (2012). Clinical observation of diabetic peripheral neuropathy by the interfere of nourishing the liver to stop the wind and tonglu decoction. Chin. J. Basic Med. Tradit. Chin. Med..

[67] Gong Y., Wang J., Tan Y., Lu J. (2013). Clinical effects of modified aconiti deccotion for diabetic perpheral neuropathy and its influences on the glucose level. J. Clin. Med. Pract..

[68] Han J. (2013). Modified Huangqi Guizhi Wuwu Decoction Combined with Mecobalamin Tablets in Treating Diabetes Clinical Observation of 62 Cases of Neuropathy. Anhui Med. Pharm. J..

[69] Zhang J. (2013). Observation of mudan tongluo fang in the treatment of type 2 diabectic peripheral neuropathy. Clin. J. Tradit. Chin. Med..

[70] Zhang Y. (2013). Observation on therapeutic effect of modified “Tangbaokang” on 60 cases of diabetic peripheral neuropathy. J. Med. Pract..

[71] Guo Y., Liu Y., Sun Y., Qin B., Cai D. (2014). Clinical observation of modified “huangqi guizi wuwu decoction” for diabetic peripheral neuropathic pain. Shanghai J. Tradit. Chin. Med..

[72] Yang Q., Chen H. (2014). Treatment of 36 Cases of Diabetic Peripheral Neuropathy with Modified Huangqi Guizi Wuwu Decoction. J. Aerosp. Med..

[73] Yang Y. (2014). Shenqixuebi Decoction and Western Medicine Treat 60 Cases of Diabetic Peripheral Neuropathy. TCM Res..

[74] Qi Y., Yu S. (2015). Effect of Mudan Granules on Oxidative Stress in Painful Diabetic Peripheral Neuropathy. Lishizhen Med. Mater. Med. Res..

[75] Wang L., Su J., Jiao H., Zhang Y., Liu T., Sin L. (2015). 40 Cases of Senile Diabetic Peripheral Neuropathy Treated with Yixinshu Capsule and Maixuekang Capsule. J. Integr. Tradit. Chin. West. Med. Cardiovasc. Dis..

[76] Xue L., Chen H., Yang Y., Zhang Z. (2015). Clinical observation on the treatment of 42 cases of diabetic peripheral neuropathy with modified liuteng shuilu shexian decoction. J. Sichuan Tradit. Chin. Med..

[77] Guo H. (2016). Clinical research of the patients with type 2 diabetic peripheral neuropathy in the elderly treated by compound qiteng tongluo tang combined epalrestat. Acta Chin. Med..

[78] Han L. (2016). Therapeutic effect of Zhanjintongluo Chinese medicine combined with mecobalamin in the treatment of patients with diabetic peripheral neuropathy. Diabetes New World.

[79] Lan B., Wang X., Mi J., Wang G. (2016). Clinic study of yiqi huoxue tongluo in treating diabetic peripheral neuropathy. Chin. Med. Mod. Distance Educ. China.

[80] Li G., Huang D., Li M., Lin L. (2016). Effects of Wenyang Huoxue Tongbi Fang on Nerve Conduction Velocity and Plasma Hcy of Diabetic Peripheral Neurophathy with Yang-Deficiency, Congealing Cold and Blood Stasis Syndrome. J. Tradit. Chin. Med..

[81] Li H., Zhong Q. (2016). Clinical observatyion of huangzhi tonglnaoluo capsule in treating diabetic periphral neuropathy. Yunnan J. Tradit. Chin. Med. Mater. Med..

[82] Mo S., Xiao T. (2016). The observation on the clinical effect of yangyin jiedu decoction in the treatment of diabetic perpheral neuropathy of qi and yin deficiency with blood stasis type. World J. Integr. Tradit. West. Med..

[83] Wang Z., An X., Chen L., Aihua C., Wen H., Lin N. (2016). Clinical study on modified tangbitong recipe for treatment of type 2 diabetes distal symmetric polyneuropathy. Guangxi Tradit. Chin. Med..

[84] Zhang H., Su H., Wang Y., Zhang B., Li H., Guo Y., Shi Z., Cui S. (2016). Observation of the clinical effect of nimodipine combined with qiming granule in the treatment of diabetic peripheral neuropathy. Inf. Tradit. Chin. Med..

[85] Zhang J., Zhi D., Xie H. (2016). Treatment of 48 cases of diabetic peripheral neuropathy with huangqichifeng decoction combined danggisini decoction. World Latest Med. Inf..

[86] Chen H., Wu J., Tan H. (2017). Clinical observation of danggui sini tang for diabetic peripheral neuropathy. J. New Chin. Med..

[87] Shi Z., Li L., Wang K., Zhang J., Lu Y., Zhang H., Wang J., Zhou Y. (2017). Effect of compound danshen dripping pills on early peripheral neuropathy in patients with type 2 diabetes. New World Diabetes.

[88] Wang P., Cui P., Hong Y. (2017). Effect of danggui sini deccotion on treatment of diabetic peripheral neuropathy with cold congealing and blood stasis. Chin. Arch. Tradit. Chin. Med..

[89] Chen X. (2018). Clinical study on danggui sini tang in treatment of peripheral neuropathy of diabetes mellitus with cold and dampness obstraction spleen syndrome. Acta Chin. Med..

[90] Dai Q., Xu X. (2018). Clinical study of huangqi guizhi wuwu decoction combined with yunu decoction in the treatment of diabectic peripheral neuropathy. Shaanxi Tradit. Chin. Med..

[91] Gao S., Tian X., Jinag W., Ma Y. (2019). Clinical Study on the Treatment of 50 Cases of Diabetic Peripheral Neuropathy with Shengmai Powder Combined with Basic Therapy. Jiangsu Tradit. Chin. Med..

[92] Hu Y., Liu H., Liu M., Wang T. (2018). Clinical observation of modified jiajian huangqi guizhi wuwu decoction in treating diabetic peripheral neuropahty patients with Qi deficiency and blood stasis type. Clin. J. Tradit. Chin. Med..

[93] Huang X., Lin X., Chen C. (2018). Clinical study on matong powder in treating 120 cases of diabetic peripheral neuritis. Mod. Hosp..

[94] She Y., Yu J., Li R., Wang Y., Zhang S., Zhu F., Hu A., Wu J., Wang J., Peng M. (2018). Observations on curative effect of huangqi guizhi wuwu granules combined with acupucnture and moxibustion in treating diabetic peripheral neuropathy. J. Guangxi Univ. Tradit. Chin. Med..

[95] Xin Y., Ma D. (2018). Observation on the clinical curative effect of mongolian medicine garidi-13 weiwan in the treatment of diabetic peripheral neuropathy. J. Med. Pharm. Chin. Minor..

[96] Ji W., Hua W. (2019). Efficacy of Yangyin Zhuyu Decoction with Epalrestat in the Treatment of Bi Disease with Yin Deficiency and Blood Stasis Syndrome Caused by Consumptive Thirst. J. Chang. Univ. Chin. Med..

[97] Liu L., Bin J., Kong F. (2019). Clinical observation on treating diabetic peripheral neuropathy with shengjiang san and taohong yin. Clin. J. Chin. Med..

[98] Liu M. (2019). Huangqi guizhi wuwu decoction combined with western Medicine treat peripheral neuropathy in type 2 diabetes mellitus randomized parallel controlled study. J. Pract. Tradit. Chin. Intern. Med..

[99] Wu G., Meng C., Zhang D. (2019). A randomized controlled study of acupuncture combined with taohong siwu decocotion in treating diabetic peripheral neuropathy. J. Gansu Univ. Chin. Med..

[100] Yi W., Zhang F., Wang Y., Sun H., Hu Y., Wu S., Liu T. (2019). Clinical observation on 54 cases of diabetic peripheral neuropathy with phlegm and static blood syndrome treated with mongolian medicine zhenbao pills. J. Tradit. Chin. Med..

[101] Chen J., Zhang Y., Hu C., Feng Z., Liu M., Shi J., Shen Y., Jiang J., Yan J. (2021). Clinical Research on TCM Directional Penetration Combined Zicui Juanbi Decoction in Treating Painful Diabetic Peripheral Neuropathy. Mod. J. Integr. Tradit. Chin. West. Med..

[102] Hou Y., Guo L., Zhang Y., Li X. (2021). Clinical study of jiuchongdan in the treatment of diabetic peripheral neuropathy. China Tradit. Chin. Med. Technol..

[103] Li Q., Zhang B. (2021). Exploration of the effect of Huanquizhiwuwu decoction combined with mudan granules in 41 cases of diabetic peripheral neuropathy (Qi deficiency and blood stasis syndrome). Anhui Med. Pharm. J..

[104] Wang R. (2021). Clinical Effect of Taohong Siwu Decoction Combined with Mecobalamin on Diabetic Peripheral Neuropathy. Diabetes New World.

[105] Wang Y., Tang L., Song H. (2021). Clinical observation of yiqi yangyin tongluo decoction in treating diabetic peripheral neuropathy. China Tradit. Chin. Med. Sci. Technol..

[106] Zhang R., Zhong Y., Zhao L. (2021). The influence of buqi huoxue zhitong tang and A-lipoic acid in patients with diabetic peripheral neuropathy on inflammatory response and neurological function of lower extremity. Chin. Prim. Health Care.

[107] Panthi S., Jing X., Gao C., Gao T. (2017). Yang-Warming Method in the Treatment of Diabetic Peripheral Neuropathy: An Updated Systematic Review and Meta-Analysis. BMC Complement. Altern. Med..

[108] Liu W., Fan Y., Tian C., Jin Y., Du S., Zeng P., Wang A. (2020). Deciphering the Molecular Targets and Mechanisms of HGWD in the Treatment of Rheumatoid Arthritis via Network Pharmacology and Molecular Docking. Evid.-Based Complement Altern. Med. ECAM.

[109] Tang Z., Huang G. (2022). Extraction, Structure, and Activity of Polysaccharide from Radix Astragali. Biomed. Pharmacother. Biomed. Pharmacother..

[110] Fu J., Wang Z., Huang L., Zheng S., Wang D., Chen S., Zhang H., Yang S. (2014). Review of the Botanical Characteristics, Phytochemistry, and Pharmacology of Astragalus Membranaceus (Huangqi). Phytother. Res. PTR.

[111] Chen Z., Liu L., Gao C., Chen W., Vong C.T., Yao P., Yang Y., Li X., Tang X., Wang S. (2020). Astragali Radix (Huangqi): A Promising Edible Immunomodulatory Herbal Medicine. J. Ethnopharmacol..

[112] Liu J., Zhang Q., Li R.-L., Wei S.-J., Huang C.-Y., Gao Y.-X., Pu X.-F. (2020). The Traditional Uses, Phytochemistry, Pharmacology and Toxicology of Cinnamomi Ramulus: A Review. J. Pharm. Pharmacol..

[113] Yao Y., Zhang X., Wang Z., Zheng C., Li P., Huang C., Tao W., Xiao W., Wang Y., Huang L. (2013). Deciphering the Combination Principles of Traditional Chinese Medicine from a Systems Pharmacology Perspective Based on Ma-Huang Decoction. J. Ethnopharmacol..

[114] Guo S., Wang J., Wang Y., Zhang Y., Bi K., Zhang Z., Li Q. (2019). Study on the Multitarget Synergistic Effects of Kai-Xin-San against Alzheimer’s Disease Based on Systems Biology. Oxid. Med. Cell Longev..

[115] Han T., Liu Y., Chen Y., Chen T., Li Y., Li Q., Zhao M. (2022). Identification of the Mechanism of Matrine Combined with Glycyrrhizin for Hepatocellular Carcinoma Treatment through Network Pharmacology and Bioinformatics Analysis. Oxid. Med. Cell Longev..

[116] Caesar L.K., Cech N.B. (2019). Synergy and Antagonism in Natural Product Extracts: When 1 + 1 Does Not Equal 2. Nat. Prod. Rep..

[117] Yuan H., Ma Q., Cui H., Liu G., Zhao X., Li W., Piao G. (2017). How Can Synergism of Traditional Medicines Benefit from Network Pharmacology?. Molecules.

[118] Gertsch J. (2011). Botanical Drugs, Synergy, and Network Pharmacology: Forth and Back to Intelligent Mixtures. Planta Med..

[119] Zhong J., Liu Z., Zhou X., Xu J. (2017). Synergic Anti-Pruritus Mechanisms of Action for the Radix Sophorae Flavescentis and Fructus Cnidii Herbal Pair. Mol. Basel Switz..

[120] Ma X., Hao C., Zhang Z., Jiang H., Zhang W., Huang J., Chen X., Yang W. (2021). Shenjinhuoxue Mixture Attenuates Inflammation, Pain, and Cartilage Degeneration by Inhibiting TLR-4 and NF-*κ*B Activation in Rats with Osteoarthritis: A Synergistic Combination of Multitarget Active Phytochemicals. Oxid. Med. Cell Longev..

[121] Ung C.Y., Li H., Cao Z.W., Li Y.X., Chen Y.Z. (2007). Are Herb-Pairs of Traditional Chinese Medicine Distinguishable from Others? Pattern Analysis and Artificial Intelligence Classification Study of Traditionally Defined Herbal Properties. J. Ethnopharmacol..

[122] Jin Y., Qu C., Tang Y., Pang H., Liu L., Zhu Z., Shang E., Huang S., Sun D., Duan J.-A. (2016). Herb Pairs Containing Angelicae Sinensis Radix (Danggui): A Review of Bio-Active Constituents and Compatibility Effects. J. Ethnopharmacol..

[123] Wang M., Bi W., Fan K., Li T., Yan T., Xiao F., He B., Bi K., Jia Y. (2018). Ameliorating Effect of Alpinia Oxyphylla—Schisandra Chinensis Herb Pair on Cognitive Impairment in a Mouse Model of Alzheimer’s Disease. Biomed. Pharmacother..

[124] Dong P.L., Li H., Yu X.J., Li Q.N., Liu J.Q., Liu C.Y., Han H. (2022). Effect and Mechanism of “Danggui–Kushen” Herb Pair on Ischemic Heart Disease. Biomed. Pharmacother..

[125] Wang X.-P., Wang P.-F., Bai J.-Q., Gao S., Wang Y.-H., Quan L.-N., Wang F., Wang X.-T., Wang J., Xie Y.-D. (2019). Investigating the Effects and Possible Mechanisms of Danshen- Honghua Herb Pair on Acute Myocardial Ischemia Induced by Isoproterenol in Rats. Biomed. Pharmacother. Biomed. Pharmacother..

[126] Zhao J., Mo C., Shi W., Meng L., Ai J. (2021). Network Pharmacology Combined with Bioinformatics to Investigate the Mechanisms and Molecular Targets of Astragalus Radix-Panax Notoginseng Herb Pair on Treating Diabetic Nephropathy. Evid.-Based Complement. Altern. Med. ECAM.

[127] Zhang X., Zhou C., Miao L., Tan Y., Zhou Y., Cheong M.S., Huang Y., Wang Y., Yu H., Cheang W.S. (2021). *Panax Notoginseng* Protects against Diabetes-Associated Endothelial Dysfunction: Comparison between Ethanolic Extract and Total Saponin. Oxid. Med. Cell Longev..

[128] Bianchi E., Rogge L. (2019). The IL-23/IL-17 Pathway in Human Chronic Inflammatory Diseases-New Insight from Genetics and Targeted Therapies. Genes Immun..

[129] Rajendran S., Quesada-Masachs E., Zilberman S., Graef M., Kiosses W.B., Chu T., Benkahla M.A., Lee J.-H.M., von Herrath M. (2021). IL-17 Is Expressed on Beta and Alpha Cells of Donors with Type 1 and Type 2 Diabetes. J. Autoimmun..

[130] Zheng Y.-H., Ren C.-Y., Shen Y., Li J.-B., Chen M.-W. (2020). A Cross-Sectional Study on the Correlation Between Inflammatory Cytokines, Negative Emotions, and Onset of Peripheral Neuropathy in Type 2 Diabetes. Neuropsychiatr. Dis. Treat..

[131] Sanaye M.M., Kavishwar S.A. (2023). Diabetic Neuropathy: Review on Molecular Mechanisms. Curr. Mol. Med..

[132] Königs V., Pierre S., Schicht M., Welss J., Hahnefeld L., Rimola V., Lütjen-Drecoll E., Geisslinger G., Scholich K. (2022). GPR40 Activation Abolishes Diabetes-Induced Painful Neuropathy by Suppressing VEGF-A Expression. Diabetes.

[133] Zhou J., Du X., Long M., Zhang Z., Zhou S., Zhou J., Qian G. (2016). Neuroprotective Effect of Berberine Is Mediated by MAPK Signaling Pathway in Experimental Diabetic Neuropathy in Rats. Eur. J. Pharmacol..

[134] Guo J., Chen H., Zhang X., Lou W., Zhang P., Qiu Y., Zhang C., Wang Y., Liu W.J. (2021). The Effect of Berberine on Metabolic Profiles in Type 2 Diabetic Patients: A Systematic Review and Meta-Analysis of Randomized Controlled Trials. Oxid. Med. Cell Longev..

[135] Costa L.G., Garrick J.M., Roquè P.J., Pellacani C. (2016). Mechanisms of Neuroprotection by Quercetin: Counteracting Oxidative Stress and More. Oxid. Med. Cell Longev..

[136] Unuofin J.O., Lebelo S.L. (2020). Antioxidant Effects and Mechanisms of Medicinal Plants and Their Bioactive Compounds for the Prevention and Treatment of Type 2 Diabetes: An Updated Review. Oxid. Med. Cell Longev..

[137] Dhanya R. (2022). Quercetin for Managing Type 2 Diabetes and Its Complications, an Insight into Multitarget Therapy. Biomed. Pharmacother..

[138] Dong B., Shi Z., Dong Y., Chen J., Wu Z.-X., Wu W., Chen Z.-S., Han C. (2022). Quercetin Ameliorates Oxidative Stress-induced Cell Apoptosis of Seminal Vesicles via Activating Nrf2 in Type 1 Diabetic Rats. Biomed. Pharmacother..

[139] Sharma D., Gondaliya P., Tiwari V., Kalia K. (2019). Kaempferol Attenuates Diabetic Nephropathy by Inhibiting RhoA/Rho-Kinase Mediated Inflammatory Signalling. Biomed. Pharmacother..

[140] Gong G., Guan Y.-Y., Zhang Z.-L., Rahman K., Wang S.-J., Zhou S., Luan X., Zhang H. (2020). Isorhamnetin: A Review of Pharmacological Effects. Biomed. Pharmacother..

[141] Wei P.-C., Lee-Chen G.-J., Chen C.-M., Chen Y., Lo Y.-S., Chang K.-H. (2022). Isorhamnetin Attenuated the Release of Interleukin-6 from *β*-Amyloid-Activated Microglia and Mitigated Interleukin-6-Mediated Neurotoxicity. Oxid. Med. Cell Longev..

[142] Matboli M., Saad M., Hasanin A.H., A Saleh L., Baher W., Bekhet M.M., Eissa S. (2021). New Insight into the Role of Isorhamnetin as a Regulator of Insulin Signaling Pathway in Type 2 Diabetes Mellitus Rat Model: Molecular and Computational Approach. Biomed. Pharmacother. Biomed. Pharmacother..

[143] Jiang H., Yamashita Y., Nakamura A., Croft K., Ashida H. (2019). Quercetin and Its Metabolite Isorhamnetin Promote Glucose Uptake through Different Signalling Pathways in Myotubes. Sci. Rep..

[144] Oza M.J., Kulkarni Y.A. (2020). Formononetin Ameliorates Diabetic Neuropathy by Increasing Expression of SIRT1 and NGF. Chem. Biodivers..

[145] Raafat K., Hdaib F. (2017). Neuroprotective Effects of Moringa Oleifera: Bio-Guided GC-MS Identification of Active Compounds in Diabetic Neuropathic Pain Model. Chin. J. Integr. Med..

