# Supplementary material for: Comparative Efficacy of East Asian Herbal Formulae Containing Astragali Radix–Cinnamomi Ramulus Herb-Pair against Diabetic Peripheral Neuropathy and Mechanism Prediction: A Bayesian Network Meta-Analysis Integrated with Network Pharmacology
Source: Pharmaceutics. 2023 Apr 28;15(5):1361. doi: 10.3390/pharmaceutics15051361 (PMC10221388; doi:10.3390/pharmaceutics15051361)
Supplement: Supplementary file 1 [file pharmaceutics-15-01361-s001.zip › Supplementary Table S1. Search strategy.pdf]

Supplementary material Table S1. Search strategies

MEDLINE

Searching strategy	
#1	mononeuropathy [MeSH] OR nerve compression syndromes[MeSH] OR neuralgia[MeSH] OR polyneuropathies [MeSH]
#2	"neuropathy"[Title/abstract] OR "peripheral neuropathy"[Title/abstract] OR "neuropathic pain"[Title/abstract] OR "neuralgia"[Title/abstract]
#3	"Plants, Medicinal"[MeSH] OR "Drugs, Chinese Herbal"[MeSH] OR "Medicine, Chinese Traditional"[MeSH] OR "Medicine, Kampo"[MeSH] OR "Medicine, Korean Traditional"[MeSH] OR "Herbal Medicine"[MeSH] OR "Prescription Drugs"[MeSH] OR "traditional Korean medicine"[Title/abstract] OR "traditional Chinese medicine"[Title/abstract] OR "traditional oriental medicine"[Title/abstract] OR "Kampo medicine"[Title/abstract] OR herb*[Title/abstract] OR decoction*[Title/abstract] OR botanic*[Title/abstract]
#4	#1 AND #2 AND #3

EMBASE

Searching strategy	
#1	('neuralgia'/exp OR neuralgia OR 'mononeuropathy'/exp OR mononeuropathy OR 'nerve'/exp OR nerve) AND ('compression'/exp OR compression) AND syndromes OR 'polyneuropathies'/exp OR polyneuropathies
#2	'peripheral neuropathy'/exp OR 'neuropathy' OR 'neuropathic pain' OR 'neuralgia'
#3	'medicinal plant'/exp OR 'medicinal plant' OR 'herbaceous agent'/exp OR 'herbaceous agent' OR 'chinese medicine'/exp OR 'chinese medicine' OR 'kampo medicine'/exp OR 'kampo medicine' OR 'kampo medicine (drug)'/exp OR 'kampo medicine (drug)' OR 'korean medicine'/exp OR 'korean medicine' OR 'herbal medicine'/exp OR 'herbal medicine' OR 'prescription drug'/exp OR 'prescription drug' OR 'oriental medicine'/exp OR 'oriental medicine' OR 'alternative medicine'/exp OR 'alternative medicine' OR 'complementary medicine' OR 'herb'/exp OR 'herb' OR 'decoction' OR 'botanic'
#4	#1 AND #2 AND #3

CENTRAL

Searching strategy	
#1	MeSH descriptor: [Mononeuropathies] explode all trees
#2	(Mononeuropathies):ti,ab,kw
#3	MeSH descriptor: [Neuralgia] explode all trees
#4	(“neuropathy” OR “peripheral neuropathy” OR “neuropathic pain” OR “neuralgia”):ti,ab,kw
#5	MeSH descriptor: [Plants, Medicinal] explode all trees
#6	MeSH descriptor: [Drugs, Chinese Herbal] explode all trees
#7	MeSH descriptor: [Medicine, Chinese Traditional] explode all trees
#8	MeSH descriptor: [Medicine, Kampo] explode all trees
#9	MeSH descriptor: [Medicine, Korean Traditional] explode all trees
#10	MeSH descriptor: [Herbal Medicine] explode all trees
#11	MeSH descriptor: [Prescription Drugs] explode all trees
#12	(“traditional Korean medicine” OR “traditional Chinese medicine” OR “Traditional oriental medicine” OR “Kampo medicine” OR herb* OR decoction* OR botanic*): ti,ab,kw
#13	(#1 OR #2) AND (#3 OR #4) AND (#5 OR #6 OR #7 OR #8 OR #9 OR #10 OR #11 OR #12) in Trials

CINAHL

	Searches
1	(MH "Diabetic Neuropathies+") or DPN
2	MH “Plants, Medicinal” OR MH “Drugs, Chinese Herbal” OR MH “Medicine, Chinese Traditional” OR MH “Medicine, Kampo” OR MH “Medicine, Korean Traditional” OR MH “Herbal Medicine” OR MH “Prescription Drugs” OR TX “traditional Korean medicine” OR TX “traditional Chinese medicine” OR TX “traditional oriental medicine” OR TX “Kampo medicine” OR TX herb* OR TX decoction* OR TX botanic*
3	(MH “Randomized controlled trials”) AND (MH “Human”)
4	#1 AND #2 AND #3

KISS

	Searches
1	(당뇨병성 신경병증 OR 당뇨병성 말초 신경병증) AND 한약

RISS

	Searches
1	(당뇨병성 신경병증 OR 당뇨병성 말초 신경병증) AND 한약

OASIS

	Searches
1	(당뇨병성 신경병증 OR 당뇨병성 말초 신경병증) AND 한약

KCI

	Searches
1	(당뇨병성 신경병증 OR 당뇨병성 말초 신경병증) AND 한약

CNKI

	Searches
1	(SU='Diabetic peripheral neuropathy'+' Diabetic Neuropathies'+'糖尿病周围神经病变'+'DPN' + '糖尿病神经病变') AND (SU='中药'+'中医药'+'本草'+'汤'+'丸'+'散'+'方'+'颗粒'+'胶囊'+'自拟'+'止痛') AND (FT='随机')

CiNii

	Searches
1	("糖尿病性末梢神経障害" OR "Diabetic peripheral neuropathy" OR "糖尿病性ニューロパチー) AND ("traditional Korean medicine" OR "traditional Chinese medicine" OR "Traditional oriental medicine" OR "Kampo medicine" OR herb OR decoction OR botanic OR 漢方薬 OR ハーブ OR 散 OR 汤 OR 丸)
