# Supplementary material for: Comparative Efficacy of East Asian Herbal Formulae Containing Astragali Radix–Cinnamomi Ramulus Herb-Pair against Diabetic Peripheral Neuropathy and Mechanism Prediction: A Bayesian Network Meta-Analysis Integrated with Network Pharmacology
Source: Pharmaceutics. 2023 Apr 28;15(5):1361. doi: 10.3390/pharmaceutics15051361 (PMC10221388; doi:10.3390/pharmaceutics15051361)
Supplement: Supplementary file 1 [file pharmaceutics-15-01361-s001.zip › Supplementary Table S2.pdf]

Supplementary Table S2. EAHM ingredients used in clinical trials included in this review

Included study	Classification of intervention types	Ingredients of EAHM prescription (Latin name)	Ingredients of EAHM prescription (Scientific name)	Type of preparations / Administration route
Jin 2004	EAWP	Astragali Radix 30g, Scrophulariae Radix 15g, Salviae Miltiorrhizae Radix 30g, Draconis Sanguis 3g, Brassicae Semen 10g, Arisaematis Rhizoma 10g	Astragalus mongholicus Bunge [Fabaceae] 30g, Scrophularia ningpoensis Hemsl. [Scrophulariaceae] 15g, Salvia miltiorrhiza Bunge [Lamiaceae] 30g, Calamus draco Willd. [Arecaceae] 3g, Brassica rapa L. [Brassicaceae] 10g, Arisaema erubescens (Wall.) Schott [Araceae] 10g	Tablet / Oral administration
Sun 2008	ECWP	Rehmanniae Radix Preparata 30g, Moutan Radicis Cortex 10g, Poria Sclerotium 10g, Alismatis Rhizoma 10g, Dioscoreae Rhizoma 15g, Corni Fructus 10g, Persicae Semen 10g, Carthami Flos 10g, Angelicae Sinensis Radix 15g, Cnidii Rhizoma 10g, Achyranthis Radix 15g, Salviae Miltiorrhizae Radix 20g, Polygoni Cuspidati Rhizoma et Radix 20g, Spatholobi Caulis 30g, Lumbricus 12g	Rehmannia glutinosa (Gaertn.) DC. [Orobanchaceae] 30g, Paeonia × suffruticosa Andrews [Paeoniaceae] 10g, Poria cocos Wolf 10g, Alisma plantago-aquatica subsp. orientale (Sam.) Sam. [Alismataceae] 10g, Dioscorea, polystachya Turcz. [Dioscoreaceae] 15g, Cornus officinalis Siebold & Zucc. [Cornaceae] 10g, Prunus persica (L.) Batsch [Rosaceae] 10g, Carthamus tinctorius L. [Asteraceae] 10g, Angelica sinensis (Oliv.) Diels [Apiaceae] 15g, Conioselinum anthriscoides 'Chuanxiong' [Apiaceae] 10g, Achyranthes bidentata Blume [Amaranthaceae] 15g, Salvia miltiorrhiza Bunge [Lamiaceae] 20g, Reynoutria japonica Houtt. [Polygonaceae] 20g, Spatholobus suberectus Dunn [Fabaceae] 30g, Pericaeta communisma Gate et Hatai 12g	Tablet / Oral administration
Shen 2009	EAWP	Acori Graminei Rhizoma, Puerariae Radix, Achyranthis Radix, Coptidis Rhizoma, Salviae Miltiorrhizae Radix, Dipsaci Radix, Leonuri Herba	Acorus gramineus Aiton [Acoraceae], Pueraria montana var. lobata (Willd.) Maesen & S.M.Almeida ex Sanjappa & Predeep [Fabaceae], Achyranthes bidentata Blume [Amaranthaceae], Coptis japonica (Thunb.) Makino [Ranunculaceae], Salvia miltiorrhiza Bunge [Lamiaceae], Dipsacus asper Wall. ex DC. [Caprifoliaceae], Leonurus japonicus Houtt. [Lamiaceae]	Capsule / Oral administration
Yan 2010	EAWP	Rehmanniae Radix Recens, Coptidis Rhizoma, Puerariae Radix, Euonymi Ramuli Suberalatum, Campsitis Flos	Rehmannia glutinosa (Gaertn.) DC. [Orobanchaceae], Coptis japonica (Thunb.) Makino [Ranunculaceae], Pueraria montana var.	Granule / Oral administration

			lobata (Willd.) Maesen & S.M.Almeida ex Sanjappa & Predeep [Fabaceae], Euonymus alatus (Thunb.) Siebold [Celastraceae], Campsis grandiflora (Thunb.) K.Schum. [Bignoniaceae]	
Lin 2010	ECWP	Ginseng Radix, Eupolyphaga, Hirudo, Scorpio, Cicadidae Periostracum, Scolopendra, Dalbergiae Odoriferae Lignum	Panax ginseng C.A.Mey. [Araliaceae], Eupolyphaga sinensis Walker, Hirudo niponica Whitman, Buthus martensii Karsch, Dalbergia odorifera T.C.Chen [Fabaceae]	Capsule / Oral administration
Wang 2010	ECCP	Astragali Radix 20g, Cinnamomi Ramulus 12g, Paeoniae Radix Alba 12g, Zingiberis Rhizoma Recens 30g, Zizyphi Fructus 4 pieces	Astragalus mongholicus Bunge [Fabaceae] 20g, Neolitsea cassia (L.) Kosterm. [Lauraceae] 12g, Paeonia lactiflora Pall. [Paeoniaceae] 12g, Zingiber officinale Roscoe [Zingiberaceae] 30g, Ziziphus jujuba Mill. [Rhamnaceae] 4 pieces	Decoction / Oral administration
Wu 2011	EAWP	Astragali Radix 30g, Puerariae Radix 30g, Dioscoreae Rhizoma 30g, Trichosanthis Radix 30g, Scrophulariae Radix 15g, Atractylodis Rhizoma Alba 10g, Poria Sclerotium 10g, Cnidii Rhizoma 10g, Moutan Radicis Cortex 10g, Angelicae Sinensis Radix 6g	Astragalus mongholicus Bunge [Fabaceae] 30g, Pueraria montana var. lobata (Willd.) Maesen & S.M.Almeida ex Sanjappa & Predeep [Fabaceae] 30g, Dioscorea polystachya Turcz. [Dioscoreaceae] 30g, Trichosanthes kirilowii Maxim. [Cucurbitaceae] 30g, Scrophularia buergeriana Miq. [Scrophulariaceae] 15g, Atractylodes macrocephala Koidz. [Asteraceae] 10g, Poria cocos Wolf 10g, Conioselinum anthriscoides 'Chuanxiong' [Apiaceae] 10g, Paeonia × suffruticosa Andrews [Paeoniaceae] 10g, Angelica sinensis (Oliv.) Diels [Apiaceae] 6g	Decoction / Oral administration
Gao 2012	ECWP	Paeoniae Radix Alba 20g, Gastrodiae Rhizoma 15g, Angelicae Sinensis Radix 10g, Cnidii Rhizoma 10g, Batryticatus Bombyx 10g, Luffae Fructus Retinervus 10g, Brassicae Semen 6g, Glycyrrhizae Radix et Rhizoma 6g	Paeonia lactiflora Pall. [Paeoniaceae] 20g, Gastrodia elata Blume [Orchidaceae] 15g, Angelica sinensis (Oliv.) Diels [Apiaceae] 10g, Conioselinum anthriscoides 'Chuanxiong' [Apiaceae] 10g, Bombyx mori [Linné] 10g, Cucumis melo L. [Cucurbitaceae] 10g, Brassica rapa L. [Brassicaceae] 6g, Glycyrrhiza uralensis Fisch. ex DC. [Fabaceae] 6g	Decoction / Oral administration
Gong 2013	ECWP	Aconiti Lateralis Radix Preparata 15g, Paeoniae Radix Alba 12g, Poria Sclerotium 12g, Atractylodis Rhizoma Alba 15g, Cnidii Rhizoma 10g, Scorpio 4g	Aconitum carmichaeli Debeaux [Ranunculaceae] 15g, Paeonia lactiflora Pall. [Paeoniaceae] 12g, Poria cocos Wolf 12g, Atractylodes macrocephala Koidz. [Asteraceae] 15g, Conioselinum	Decoction / Oral administration

			anthriscoides 'Chuanxiong' [Apiaceae] 10g, Buthus martensii Karsch 4g	
Han 2013	ECCP	Astragali Radix 20g, Cinnamomi Ramulus 6g, Spatholobi Caulis 20g, Paeoniae Radix Alba 15g, Glycyrrhizae Radix et Rhizoma 6g	Astragalus mongholicus Bunge [Fabaceae] 20g, Neolitsea cassia (L.) Kosterm. [Lauraceae] 6g, Spatholobus suberectus Dunn [Fabaceae] 20g, Paeonia lactiflora Pall. [Paeoniaceae] 15g, Glycyrrhiza uralensis Fisch. ex DC. [Fabaceae] 6g	Decoction / Oral administration
Guo 2014	ECCP	Astragali Radix 30g, Cinnamomi Ramulus 12g, Spatholobi Caulis 30g, Paeoniae Radix Alba 9g, Paeniae Radix Rubra 9g, Angelicae Sinensis Radix 9g, Salviae Miltiorrhizae Radix 15g, Zizyphi Fructus 7 pieces, Zingiberis Rhizoma Recens 10g, Curcumae Longae Rhizoma 10g	Astragalus mongholicus Bunge [Fabaceae] 30g, Neolitsea cassia (L.) Kosterm. [Lauraceae] 12g, Spatholobus suberectus Dunn [Fabaceae] 30g, Paeonia lactiflora Pall. [Paeoniaceae] 9g, Paeonia anomala subsp. veitchii (Lynch) D.Y.Hong & K.Y.Pan [Paeoniaceae] 9g, Angelica sinensis (Oliv.) Diels [Apiaceae] 9g, Ziziphus jujuba Mill. [Rhamnaceae] 7 pieces, Salvia miltiorrhiza Bunge [Lamiaceae] 15g, Zingiber officinale Roscoe [Zingiberaceae] 10g, Curcuma longa L. [Zingiberaceae] 10g	Decoction / Oral administration
Zhang 2013a	ECWP	Chaenomelis Fructus 30g, Moutan Radicis Cortex 20g, Coptidis Rhizoma 6g, Rehmanniae Radix Preparata 12g, Spatholobi Caulis 15g	Pseudocydonia sinensis (Dum.Cours.) C.K.Schneid. [Rosaceae] 30g, Paeonia × suffruticosa Andrews [Paeoniaceae] 20g, Coptis japonica (Thunb.) Makino [Ranunculaceae] 6g, Rehmannia glutinosa (Gaertn.) DC. [Orobanchaceae] 12g, Spatholobus suberectus Dunn [Fabaceae] 15g	Decoction / Oral administration
Zhang 2013b	EAWP	Astragali Radix 3g, Salviae Miltiorrhizae Radix 3g, Polygonati Rhizoma 3g, Atractylodis Rhizoma 2g, Cnidii Rhizoma 2g, Mori Ramulus 2g, Piperis Kadsurae Caulis 1g, Mori Radicis Cortex 1g	Astragalus mongholicus Bunge [Fabaceae] 3g, Salvia miltiorrhiza Bunge [Lamiaceae] 3g, Polygonatum sibiricum Redouté [Asparagaceae] 3g, Atractylodes macrocephala Koidz. [Asteraceae] 2g, Conioselinum anthriscoides 'Chuanxiong' [Apiaceae] 2g, Morus alba L. [Moraceae] 1g, Piper kadsura (Choisy) Ohwi [Piperaceae]1g, Morus alba L. [Moraceae] 1g	Pill / Oral administration
Yang 2014a	ECCP	Astragali Radix 60g, Cinnamomi Ramulus 15g, Spatholobi Caulis 30g, Panacis Quinquefolii Radix 12g,	Astragalus mongholicus Bunge [Fabaceae] 60g, Neolitsea cassia (L.) Kosterm. [Lauraceae] 15g, Spatholobus suberectus Dunn	Decoction / Oral administration

		Poria Sclerotium 30g, Angelicae Sinensis Radix 20g, Rehmanniae Radix Preparata 30g, Corni Fructus 25g, Bupleuri Radix 12g, Hirudo 6g, Cnidii Rhizoma 30g, Sappan Lignum 15g, Paeoniae Radix Alba 20g, Lonicerae Flos 15g, Cyathulae Radix 30g	[Fabaceae] 30g, Panax quinquefolius L. [Araliaceae] 12g, Poria cocos Wolf 30g, Angelica sinensis (Oliv.) Diels [Apiaceae] 20g, Rehmannia glutinosa (Gaertn.) DC. [Orobanchaceae] 30g, Cornus officinalis Siebold & Zucc. [Cornaceae] 25g, Bupleurum falcatum L. [Apiaceae] 12g, Hirudo niponica Whitman 6g, Conioselinum anthriscoides 'Chuanxiong' [Apiaceae] 30g, Biancaea sappan (L.) Tod. [Fabaceae] 15g, Paeonia lactiflora Pall. [Paeoniaceae] 20g, Lonicera japonica Thunb. [Caprifoliaceae] 15g, Cyathula officinalis K.C.Kuan [Amaranthaceae] 30g	
Yang 2014b	ECCP	Astragali Radix 50g, Cinnamomi Ramulus 10g, Spatholobi Caulis 25g, Angelicae Sinenssis Radix 15g, Paeoniae Radix Alba 12g, Melandrii Herba 15g, Zingiberis Rhizoma Recens 10g, Zizyphi Fructus 15g	Astragalus mongholicus Bunge [Fabaceae] 50g, Neolitsea cassia (L.) Kosterm. [Lauraceae] 10g, Spatholobus suberectus Dunn [Fabaceae] 25g, Angelica sinensis (Oliv.) Diels [Apiaceae] 15g, Paeonia lactiflora Pall. [Paeoniaceae] 12g, Silene firma Siebold & Zucc. [Caryophyllaceae] 15g, Zingiber officinale Roscoe [Zingiberaceae] 10g, Ziziphus jujuba Mill. [Rhamnaceae] 15g	Decoction / Oral administration
Qi 2015	ECWP	Astragali Radix, Spatholobi Caulis, Corydalis Tuber, Notoginseng Radix et Rhizoma, Paeoniae Radix Rubra, Salviae Miltiorrhizae Radix, Cnidii Rhizoma, Carthami Flos, Sappan Lignum	Astragalus mongholicus Bunge [Fabaceae], Spatholobus suberectus Dunn [Fabaceae], Corydalis ternata (Nakai) Nakai [Papaveraceae], Panax notoginseng (Burkill) F.H.Chen [Araliaceae], Paeonia anomala subsp. veitchii (Lynch) D.Y.Hong & K.Y.Pan [Paeoniaceae] , Salvia miltiorrhiza Bunge [Lamiaceae], Conioselinum anthriscoides 'Chuanxiong' [Apiaceae], Carthamus tinctorius L. [Asteraceae], Biancaea sappan (L.) Tod. [Fabaceae]	Granule / Oral administration
Wang 2015	EAWP	Astragali Radix, Ginseng Radix, Liriopis seu Ophiopogonis Tuber, Schisandrae Fructus, Cnidii Rhizoma, Salviae Miltiorrhizae Radix, Crataegi Fructus, Hirudo	Astragalus mongholicus Bunge [Fabaceae], Panax ginseng C.A.Mey. [Araliaceae], Liriope muscari (Decne.) L.H.Bailey [Asparagaceae], Schisandra chinensis (Turcz.) Baill. [Schisandraceae], Conioselinum anthriscoides 'Chuanxiong' [Apiaceae], Salvia miltiorrhiza Bunge [Lamiaceae],	Capsule / Oral administration

			Crataegus pinnatifida Bunge [Rosaceae], Hirudo niponica Whitman	
Xue 2015	EACP	Astragali Radix 30g, Cinnamomi Ramulus 20g, Spatholobi Caulis 30g, Piperis Kadsurae Caulis 30g, Sinomeni Caulis et Rhizoma 30g, Trachelospermi Caulis 30g, Lonicerae Folium et Caulis 30g, Uncariae Ramulus cum Uncus 30g, Clematidis Radix 15g, Zaocys 15g, Hirudo 10g, Persicae Semen 20g, Carthami Flos 20g	Astragalus mongholicus Bunge [Fabaceae] 30g, Neolitsea cassia (L.) Kosterm. [Lauraceae] 20g, Spatholobus suberectus Dunn [Fabaceae] 30g, Piper kadsura (Choisy) Ohwi [Piperaceae] 30g, Sinomenium acutum (Thunb.) Rehder & E.H.Wilson [Menispermaceae] 30g, Trachelospermum asiaticum (Siebold & Zucc.) Nakai [Apocynaceae] 30g, Uncaria sinensis (Oliv.) Havil. [Rubiaceae] 30g, Clematis terniflora var. mandshurica (Rupr.) Ohwi [Ranunculaceae] 15g, Zaocys dhumnades Cantor 15g, Hirudo niponica Whitman 10g, Prunus persica (L.) Batsch [Rosaceae] 20g, Carthamus tinctorius L. [Asteraceae] 20g	Decoction / oral administration
Guo 2016	ECWP	Astragali Radix 30g, Spatholobi Caulis 25g, Chaenomelis Fructus 15g, Coicis Semen 15g, Atractylodis Rhizoma 15g, Tetrapanacis Medulla 15g, Benincasae Semen 15g, Mori Ramulus 15g, Indigo Pulverata Levis 15g, Tokoro Rhizoma 15g, Achyranthis Radix 15g, Phellodendri Cortex 15g, Hirudo 15g	Astragalus mongholicus Bunge [Fabaceae] 30g, Spatholobus suberectus Dunn [Fabaceae] 25g, Chaenomeles lagenaria (Loisel.) Koidz. [Rosaceae] 15g, Coix lacryma-jobi var. ma-yuen (Rom.Caill.) Stapf [Poaceae] 15g, Atractylodes macrocephala Koidz. [Asteraceae] 15g, Tetrapanax papyrifer (Hook.) K.Koch [Araliaceae] 15g, Benincasa hispida (Thunb.) Cogn. [Cucurbitaceae] 15g, Morus alba L. [Moraceae] 15g, Persicaria tinctoria (Aiton) Spach [Polygonaceae] 15g, Dioscorea tokoro Makino ex Miyabe [Dioscoreaceae] 15g, Achyranthes bidentata Blume [Amaranthaceae] 15g, Phellodendron amurense Rupr. [Rutaceae] 15g, Hirudo niponica Whitman 15g	Decoction / Oral administration
Han 2016	ECWP	Angelicae Sinensis Radix 6g, Rehmanniae Radix Recens 10g, Cnidii Rhizoma 15g, Paeoniae Radix Rubra 10g, Salviae Miltiorrhizae Radix 10g, Leonuri Herba 6g, Aucklandiae Radix 6g, Puerariae Radix 10g	Angelica sinensis (Oliv.) Diels [Apiaceae] 6g, Rehmannia glutinosa (Gaertn.) DC. [Orobanchaceae] 10g, Conioselinum anthriscoides 'Chuanxiong' [Apiaceae] 15g, Paeonia anomala subsp. veitchii (Lynch) D.Y.Hong & K.Y.Pan [Paeoniaceae] 10g,	Decoction / Oral administration

			Salvia miltiorrhiza Bunge [Lamiaceae] 10g, Leonurus japonicus Houtt. [Lamiaceae] 6g, Aucklandia costus Falc. [Asteraceae] 6g, Pueraria montana var. lobata (Willd.) Maesen & S.M.Almeida ex Sanjappa & Predeep [Fabaceae] 10g	
Lan 2016	EACP	Astragali Radix, Cinnamomi Ramulus, Spatholobi Caulis, Siegesbeckiae Herba, Clematidis Radix, Achyranthis Radix, Scorpio	Astragalus mongholicus Bunge [Fabaceae], Neolitsea cassia (L.) Kosterm. [Lauraceae], Spatholobus suberectus Dunn [Fabaceae], Sigesbeckia glabrescens (Makino) Makino [Asteraceae], Clematis terniflora var. mandshurica (Rupr.) Ohwi [Ranunculaceae], Achyranthes bidentata Blume [Amaranthaceae], Buthus martensii Karsch	Capsule / Oral administration
Mo 2016	EAWP	Astragali Radix 20g, Lonicerae Flos 30g, Scrophulariae Radix 30g, Schisandrae Fructus 12g, Puerariae Radix 20g, Polygonati Odorati Rhizoma 20g, Salviae Miltiorrhizae Radix 30g, Litchi Semen 30g, Glycyrrhizae Radix et Rhizoma 10g	Astragalus mongholicus Bunge [Fabaceae] 20g, Lonicera japonica Thunb. [Caprifoliaceae] 30g, Scrophularia buergeriana Miq. [Scrophulariaceae] 30g, Schisandra chinensis (Turcz.) Baill. [Schisandraceae] 12g, Pueraria montana var. lobata (Willd.) Maesen & S.M.Almeida ex Sanjappa & Predeep [Fabaceae] 20g, Polygonatum odoratum var. pluriflorum (Miq.) Ohwi [Asparagaceae] 20g, Salvia miltiorrhiza Bunge [Lamiaceae] 30g, Litchi chinensis Sonn. [Sapindaceae] 30g, Glycyrrhiza uralensis Fisch. ex DC. [Fabaceae] 10g	Decoction / Oral administration
Wang 2016	EACP	Astragali Radix 30g, Cinnamomi Ramulus 10g, Spatholobi Caulis 30g, Paeoniae Radix Alba 15g, Paeoniae Radix Rubra 15g, Corydalis Tuber 15g, Angelicae Sinensis Radix 10g, Salviae Miltiorrhizae Radix 20g, Lumbricus 10g, Clematidis Radix 30g, Lycopodii Herba 15g, Achyranthis Radix 10g, Liriopis seu Ophiopogonis Tuber 10g	Astragalus mongholicus Bunge [Fabaceae] 30g, Neolitsea cassia (L.) Kosterm. [Lauraceae] 10g, Spatholobus suberectus Dunn [Fabaceae] 30g, Paeonia lactiflora Pall. [Paeoniaceae] 15g, Paeonia anomala subsp. veitchii (Lynch) D.Y.Hong & K.Y.Pan [Paeoniaceae] 15g, Corydalis yanhusuo (Y.H.Chou & Chun C.Hsu) W.T.Wang ex Z.Y.Su & C.Y.Wu [Papaveraceae] 15g, Angelica sinensis (Oliv.) Diels [Apiaceae] 10g, Salvia miltiorrhiza Bunge [Lamiaceae] 20g, Pericaeta communisma Gate et Hatai 10g, Clematis terniflora var. mandshurica (Rupr.) Ohwi [Ranunculaceae] 30g, Lycopodium	Decoction / Oral administration

			clavatum L. [Lycopodiaceae] 15g, Achyranthes bidentata Blume [Amaranthaceae] 10g, Liriope muscari (Decne.) L.H.Bailey [Asparagaceae] 10g	
Li 2016a	ECCP	Astragali Radix 15g, Cinnamomi Ramulus 10g, Spatholobi Caulis 30g, Aconiti Lateralis Radix Preparata 6g, Eucommiae Cortex 15g, Achyranthis Radix 15g, Angelicae Sinensis Radix 10g, Codonopsis Pilosulae Radix 15g, Brassicae Semen 10g, Angelicae Dahuricae Radix 15g, Lycopodii Herba 10g, Myrrha Scolopendra 2 pieces, Corydalis Tuber, 20g Cnidii Rhizoma 10g, Lumbricus 10g	Astragalus mongholicus Bunge [Fabaceae] 15g, Neolitsea cassia (L.) Kosterm. [Lauraceae] 10g, Spatholobus suberectus Dunn [Fabaceae] 30g, Aconitum carmichaeli Debeaux [Ranunculaceae] 6g, Eucommia ulmoides Oliv. [Eucommiaceae] 15g, Achyranthes bidentata Blume [Amaranthaceae] 15g, Angelica sinensis (Oliv.) Diels [Apiaceae] 10g, Codonopsis pilosula (Franch.) Nannf. [Campanulaceae] 15g, Brassica rapa L. [Brassicaceae] 10g, Angelica dahurica (Hoffm.) Benth. & Hook.f. ex Franch. & Sav. [Apiaceae] 15g, Lycopodium clavatum L. [Lycopodiaceae] 10g, Scolopendra subspinipes mutilans Linné Koch pieces, Corydalis yanhusuo (Y.H.Chou & Chun C.Hsu) W.T.Wang ex Z.Y.Su & C.Y.Wu [Papaveraceae] 20g, Conioselinum anthriscoides 'Chuanxiong' [Apiaceae] 10g, Pericaeta communisma Gate et Hatai 10g	Decoction / oral administration
Zhang 2016a	ECCP	Astragali Radix 30g, Cinnamomi Ramulus 15g, Paeoniae Radix Rubra 15g, Saposhnikoviae Radix 10g, Angelicae Sinenis Radix 15g, Tetrapanacis Medulla 10g, Asiasari Radix et Rhizoma 5g, Glycyrrhizae Radix et Rhizoma 6g, Cnidii Rhizoma 15g, Speranskiae Tuberculatae Herba 20g, Lycopodii Herba 20g	Astragalus mongholicus Bunge [Fabaceae] 30g, Neolitsea cassia (L.) Kosterm. [Lauraceae] 15g, Paeonia anomala subsp. veitchii (Lynch) D.Y.Hong & K.Y.Pan [Paeoniaceae] 15g, Saposhnikovia divaricata (Turcz. ex Ledeb.) Schischk. [Apiaceae] 10g, Angelica sinensis (Oliv.) Diels [Apiaceae] 15g, Tetrapanax papyrifer (Hook.) K.Koch [Araliaceae] 10g, Asarum sieboldii Miq. [Aristolochiaceae] 5g, Glycyrrhiza uralensis Fisch. ex DC. [Fabaceae] 6g, Conioselinum anthriscoides 'Chuanxiong' [Apiaceae] 15g, Speranskia tuberculata (Bunge) Baill. [Euphorbiaceae] 20g, Lycopodium clavatum L. [Lycopodiaceae] 20g	Decoction / Oral administration
Li 2016b	EAWP	Astragali Radix, Notoginseng Radix et Rhizoma, Hirudo,	Astragalus mongholicus Bunge [Fabaceae], Panax notoginseng	Capsule / Oral

		Clematidis Radix, Achyranthis Radix	(Burkill) F.H.Chen [Araliaceae], Hirudo niponica Whitman, Clematis terniflora var. mandshurica (Rupr.) Ohwi [Ranunculaceae], Achyranthes bidentata Blume [Amaranthaceae]	administration
Zhang 2016b	ECCP	Astragali Radix, Puerariae Radix, Rehmanniae Radix, Lycii Fructus, Cassiae Semen, Leonuri Semen, Typhae Pollen, Hirudo	Astragalus mongholicus Bunge [Fabaceae], Pueraria montana var. lobata (Willd.) Maesen & S.M.Almeida ex Sanjappa & Predeep [Fabaceae], Rehmannia glutinosa (Gaertn.) DC. [Orobanchaceae], Lycium chinense Mill. [Solanaceae], Senna obtusifolia (L.) H.S.Irwin & Barneby [Fabaceae], Leonurus sibiricus L. [Lamiaceae], Typha orientalis C.Presl [Typhaceae], Hirudo niponica Whitman	Granule / Oral administration
Chen 2017	EACP	Astragali Radix 30g, Cinnamomi Ramulus 30g, Paeoniae Radix Rubra 30g, Zingiberis Rhizoma Recens 30g, Angelicae Sinensis Radix 20g, Glycyrrhizae Radix et Rhizoma 20g, Asiasari Radix et Rhizoma 15g, Aconiti Lateralis Radix Preparata 10g, Zingiberis Rhizoma 10g, Tetrapanacis Medulla 10g	Astragalus mongholicus Bunge [Fabaceae] 30g, Neolitsea cassia (L.) Kosterm. [Lauraceae] 30g, Paeonia anomala subsp. veitchii (Lynch) D.Y.Hong & K.Y.Pan [Paeoniaceae] 30g , Zingiber officinale Roscoe [Zingiberaceae] 10g, Angelica sinensis (Oliv.) Diels [Apiaceae] 20g, Glycyrrhiza uralensis Fisch. ex DC. [Fabaceae] 20g, Asarum sieboldii Miq. [Aristolochiaceae] 15g, Aconitum carmichaeli Debeaux [Ranunculaceae] 10g, Zingiber officinale Roscoe [Zingiberaceae] 10g, Tetrapanax papyrifer (Hook.) K.Koch [Araliaceae] 10g	Decoction / Oral administration
Shi 2017	EAWP	Salviae Miltiorrhizae Radix, Bomeolum, Notoginseng Radix et Rhizoma	Salvia miltiorrhiza Bunge [Lamiaceae], Dryobalanops aromatica C.F.Gaertn. [Dipterocarpaceae], Panax notoginseng (Burkill) F.H.Chen [Araliaceae]	Pill / Oral administration
Wang 2017	EAWP	Cinnamomi Ramulus 15g, Asiasari Radix et Rhizoma 5g, Angelicae Sinensis Radix 15g, Glycyrrhizae Radix et Rhizoma 10g, Paeoniae Radix Alba 15g, Zizyphi Fructus 10g, Tetrapanacis Medulla 10g	Neolitsea cassia (L.) Kosterm. [Lauraceae] 15g, Asarum sieboldii Miq. [Aristolochiaceae] 5g, Angelica sinensis (Oliv.) Diels [Apiaceae] 15g, Glycyrrhiza uralensis Fisch. ex DC. [Fabaceae] 10g, Paeonia lactiflora Pall. [Paeoniaceae] 15g, Ziziphus jujuba Mill. [Rhamnaceae] 10g, Tetrapanax papyrifer (Hook.) K.Koch [Araliaceae] 10g	Decoction / Oral administration
Chen 2018	ECWP	Angelicae Sinensis Radix 20g, Paeoniae Radix Alba 15g,	Angelica sinensis (Oliv.) Diels [Apiaceae] 20g, Paeonia lactiflora	Decoction / Oral

		Asiasari Radix et Rhizoma 10g, Cinnamomi Ramulus 15g, Tetrapanacis Medulla 10g, Zizyphi Fructus 5 pieces, Glycyrrhizae Radix et Rhizoma 10g	Pall. [Paeoniaceae] 15g, Asarum sieboldii Miq. [Aristolochiaceae] 10g, Neolitsea cassia (L.) Kosterm. [Lauraceae] 15g, Tetrapanax papyrifer (Hook.) K.Koch [Araliaceae] 10g, Ziziphus jujuba Mill. [Rhamnaceae] pieces, Glycyrrhiza uralensis Fisch. ex DC. [Fabaceae] 10g	administration
Dai 2018	EACP	Astragali Radix 30g, Cinnamomi Ramulus 10g, Carthami Flos 10g, Paeoniae Radix Alba 30g, Rehmanniae Radix Preparata 30g, Achyranthis Radix 15g, Gypsum Fibrosum 15g, Liriopis seu Ophiopogonis Tuber 10g, Anemarrhenae Rhizoma 10g, Angelicae Sinensis Radix 10g, Persicae Semen 10g, Zingiberis Rhizoma Recens 10g, Zizyphi Fructus 10g, Scorpio 10g, Lumbricus 10g, Scolopendra 10g	Astragalus mongholicus Bunge [Fabaceae] 30g, Neolitsea cassia (L.) Kosterm. [Lauraceae] 10g, Carthamus tinctorius L. [Asteraceae] 10g, Paeonia lactiflora Pall. [Paeoniaceae] 30g, Rehmannia glutinosa (Gaertn.) DC. [Orobanchaceae] 30g, Achyranthes bidentata Blume [Amaranthaceae] 15g, Gypsum Fibrosum 15g, Liriope muscari (Decne.) L.H.Bailey [Asparagaceae] 10g, Anemarrhena asphodeloides Bunge [Asparagaceae] 10g, Angelica sinensis (Oliv.) Diels [Apiaceae] 10g, Prunus persica (L.) Batsch [Rosaceae] 10g, Zingiber officinale Roscoe [Zingiberaceae] 10g, Ziziphus jujuba Mill. [Rhamnaceae] 10g, Buthus martensii Karsch 10g, Pericaeta communisma Gate et Hatai 10g, Scolopendra subspinipes mutilans Linné Koch 10g	Decoction / Oral administration
Hu 2018	ECCP	Astragali Radix 30g, Cinnamomi Ramulus 10g, Spatholobi Caulis 15g, Paeoniae Radix Rubra 20g, Zingiberis Rhizoma Recens 15g, Carthami Flos 12g, Angelicae Sinensis Radix 12g, Persicae Semen 12g, Rehmanniae Radix Recens 20g, Codonopsis Pilosulae Radix 15g, Glycyrrhizae Radix et Rhizoma 6g, Cnidii Rhizoma 10g	Astragalus mongholicus Bunge [Fabaceae] 30g, Neolitsea cassia (L.) Kosterm. [Lauraceae] 10g, Spatholobus suberectus Dunn [Fabaceae] 15g, Paeonia anomala subsp. veitchii (Lynch) D.Y.Hong & K.Y.Pan [Paeoniaceae] 20g, Zingiber officinale Roscoe [Zingiberaceae] 15g, Carthamus tinctorius L. [Asteraceae] 12g, Angelica sinensis (Oliv.) Diels [Apiaceae] 12g, Prunus persica (L.) Batsch [Rosaceae] 12g, Rehmannia glutinosa (Gaertn.) DC. [Orobanchaceae] 20g, Codonopsis pilosula (Franch.) Nannf. [Campanulaceae] 15g, Glycyrrhiza uralensis Fisch. ex DC. [Fabaceae] 6g, Conioselinum anthriscoides 'Chuanxiong' [Apiaceae] 10g	Decoction / Oral administration

Huang 2018	EAWP	Myrrha 6g, Achyranthis Radix 13g, Aconiti Lateralis Radix Preparata 6g, Astragali Radix 20g, Mori Ramulus 13g, Persicae Semen 10g, Paeoniae Radix Alba 10g, Carthami Flos 3g, Ephedrae Herba 3g, Codonopsis Pilosulae Radix 15g, Notoginseng Radix et Rhizoma 3g, Olibanum 6g, Eupolyphaga 6g, Angelicae Sinensis Radix 10g, Asiasari Radix et Rhizoma 3g	Commiphora myrrha (T.Nees) Engl. [Burseraceae] 6g, Achyranthes bidentata Blume [Amaranthaceae] 13g, Aconitum carmichaeli Debeaux [Ranunculaceae] 6g, Astragalus mongholicus Bunge [Fabaceae] 20g, Morus alba L. [Moraceae] 13g, Prunus persica (L.) Batsch [Rosaceae] 10g, Paeonia lactiflora Pall. [Paeoniaceae] 10g, Carthamus tinctorius L. [Asteraceae] 3g, Ephedra intermedia Schrenk & C.A.Mey. [Ephedraceae] 3g, Codonopsis pilosula (Franch.) Nannf. [Campanulaceae] 15g, Panax notoginseng (Burkill) F.H.Chen [Araliaceae] 3g, Boswellia carteri Birdw. [Burseraceae] 6g, Eupolyphaga sinensis Walker 6g, Angelica sinensis (Oliv.) Diels [Apiaceae] 10g, Asarum sieboldii Miq. [Aristolochiaceae] 3g	Powder / Oral administration
She 2018	ECCP	Astragali Radix 20g, Cinnamomi Ramulus 10g, Paeoniae Radix 10g, Zingiberis Rhizoma Recens 20g, Zizyphi Fructus 3 pieces	Astragalus mongholicus Bunge [Fabaceae] 20g, Neolitsea cassia (L.) Kosterm. [Lauraceae] 10g, Paeonia lactiflora Pall. [Paeoniaceae] 10g, Zingiber officinale Roscoe [Zingiberaceae] 20g, Ziziphus jujuba Mill. [Rhamnaceae] 3 pieces	Granule / Oral administration
Xin 2018	EAWP	Aconiti Kusnezoffii Tuber, Moschus, Acori Graminei Rhizoma, Aucklandiae Radix, Terminaliae Fructus, Magenetitum, Margarita, Glycyrrhizae Radix et Rhizoma, Syzygii Flos, Myristicae Semen, Aquilariae Lignum, Limonitum	Aconitum kusnezoffii Rchb. [Ranunculaceae], Abelmoschus moschatus Medik. [Malvaceae], Acorus gramineus Aiton [Acoraceae], Aucklandia costus Falc. [Asteraceae], Terminalia chebula Retz. [Combretaceae], Magenetitum, Pinctada fucada martensii (Dunker), Glycyrrhiza uralensis Fisch. ex DC. [Fabaceae], Syzygium aromaticum (L.) Merr. & L.M.Perry [Myrtaceae], Myristica fragrans Houtt. [Myristicaceae], Aquilaria malaccensis Lam. [Thymelaeaceae], Limonitum	Pill / Oral administration
Gao 2019	ECWP	Astragali Radix 20g, Scorpio 6g, Euonymi Ramuli Suberalatum 10g, Salviae Miltiorrhizae Radix 15g, Cibotii Rhizoma 15g, Dipsaci Radix 15g, Liriopis seu Ophiopogonis Tuber 15g, Corni Fructus 15g, Ginseng Radix 20g, Schisandrae Fructus 15g	Astragalus mongholicus Bunge [Fabaceae] 20g, Buthus martensii Karsch 6g, Euonymus alatus (Thunb.) Siebold [Celastraceae] 10g, Salvia miltiorrhiza Bunge [Lamiaceae] 15g, Cibotium barometz (L.) J.Sm. [Cyatheaceae] 15g, Dipsacus asper Wall. ex DC. [Caprifoliaceae] 15g, Liriope muscari (Decne.) L.H.Bailey	Decoction / Oral administration

			[Asparagaceae] 15g, Cornus officinalis Siebold & Zucc. [Cornaceae] 15g, Panax ginseng C.A.Mey. [Araliaceae] 20g, Schisandra chinensis (Turcz.) Baill. [Schisandraceae] 15g	
Wu 2019	EAWP	Angelicae Sinensis Radix 15g, Paeoniae Radix Alba 15g, Cnidii Rhizoma 10g, Rehmanniae Radix Preparata 15g, Persicae Semen 15g, Carthami Flos 15g	Angelica sinensis (Oliv.) Diels [Apiaceae] 15g, Paeonia lactiflora Pall. [Paeoniaceae] 15g, Conioselinum anthriscoides 'Chuanxiong' [Apiaceae] 10g, Rehmannia glutinosa (Gaertn.) DC. [Orobanchaceae] 15g, Prunus persica (L.) Batsch [Rosaceae] 15g, Carthamus tinctorius L. [Asteraceae] 15g	Decoction / Oral administration
Yi 2019	EAWP	Moschus, Dalbergiae Odoriferae Lignum, Inula racemosa, Aucklandiae Radix, Carthami Flos, Lagotis brachystachya Maxim, Piperis Longi Fructus, Syzygii Flos, Alpiniae Officinari Rhizoma, Fructus cymini, Cinnamomi Cortex, Myristicae Semen, Margarita, Olibanum, Bubali Cornu, Bovis Calculus Artifactus	Abelmoschus moschatus Medik. [Malvaceae], Dalbergia odorifera T.C.Chen [Fabaceae], Inula racemosa Hook.f. [Asteraceae], Aucklandia costus Falc. [Asteraceae], Carthamus tinctorius L. [Asteraceae], Lagotis brachystachya Maxim. [Plantaginaceae], Piper longum L. [Piperaceae], Syzygium aromaticum (L.) Merr. & L.M.Perry [Myrtaceae], Alpinia officinarum Hance [Zingiberaceae], Cuminum cyminum L. [Apiaceae], Neolitsea cassia (L.) Kosterm. [Lauraceae], Myristica fragrans Houtt. [Myristicaceae], Pinctada fucada martensii (Dunker), Boswellia carteri Birdw. [Burseraceae], Bubalus bubalis L., Bos taurus L.	Pill / Oral administration
Ji 2019	ECCP	Astragali Radix 10g, Cinnamomi Ramulus 10g, Codonopsis Pilosulae Radix 15g, Liriopis seu Ophiopogonis Tuber 15g, Schisandrae Fructus 10g, Salviae Miltiorrhizae Radix 15g, Amomi Fructus 5g, Dalbergiae Odoriferae Lignum 8g, Asiasari Radix et Rhizoma 3g, Angelicae Sinensis Radix 12g, Cnidii Rhizoma 10g, Paeoniae Radix Alba 10g, Carthami Flos 10g, Achyranthis Radix 10g, Notoginseng Radix et Rhizoma 2g, Lumbricus 10g, Glycyrrhizae Radix et Rhizoma 3g	Astragalus mongholicus Bunge [Fabaceae] 10g, Neolitsea cassia (L.) Kosterm. [Lauraceae] 10g, Codonopsis pilosula (Franch.) Nannf. [Campanulaceae] 15g, Liriope muscari (Decne.) L.H.Bailey [Asparagaceae] 15g, Schisandra chinensis (Turcz.) Baill. [Schisandraceae] 10g, Salvia miltiorrhiza Bunge [Lamiaceae] 15g, Lanxangia tsao-ko (Crevost & Lemarié) M.F.Newman & Skornick. [Zingiberaceae] 5g, Dalbergia odorifera T.C.Chen [Fabaceae] 8g, Asarum sieboldii Miq. [Aristolochiaceae] 3g, Angelica sinensis (Oliv.) Diels [Apiaceae] 12g, Conioselinum anthriscoides	Decoction / Oral administration

			'Chuanxiong' [Apiaceae] 10g, Paeonia lactiflora Pall. [Paeoniaceae] 10g, Carthamus tinctorius L. [Asteraceae] 10g, Achyranthes bidentata Blume [Amaranthaceae] 10g, Panax notoginseng (Burkill) F.H.Chen [Araliaceae] 2g, Pericaeta communisma Gate et Hatai 10g, Glycyrrhiza uralensis Fisch. ex DC. [Fabaceae] 3g	
Liu 2019a	ECWP	Batryticatus Bombyx 10g, Cicadidae Periostracum 10g, Rhei Radix et Rhizoma 10g, Curcumae Longae Rhizoma 10g, Persicae Semen 12g, Carthami Flos 10g, Cnidii Rhizoma 10g, Angelicae Sinensis Radix 15g, Clematidis Radix 20g	Bombyx mori L. 10g, Cryptotympana dubia (Haupt) 10g, Rheum officinale Baill. [Polygonaceae] 10g, Curcuma longa L. [Zingiberaceae] 10g, Prunus persica (L.) Batsch [Rosaceae] 12g, Carthamus tinctorius L. [Asteraceae] 10g, Conioselinum anthriscoides 'Chuanxiong' [Apiaceae] 10g, Angelica sinensis (Oliv.) Diels [Apiaceae] 15g, Clematis terniflora var. mandshurica (Rupr.) Ohwi [Ranunculaceae] 20g	Decoction / Oral administration
Liu 2019b	ECCP	Astragali Radix 30g, Cinnamomi Ramulus 10g, Spatholobi Caulis 30g, Zingiberis Rhizoma Recens 12g, Atractylodis Rhizoma Alba 12g, Lumbricus 10g, Codonopsis Pilosulae Radix 10g, Paeoniae Radix Alba 12g, Zizyphi Fructus 9g, Glycyrrhizae Radix et Rhizoma 6g	Astragalus mongholicus Bunge [Fabaceae] 30g, Neolitsea cassia (L.) Kosterm. [Lauraceae] 10g, Spatholobus suberectus Dunn [Fabaceae] 30g, Zingiber officinale Roscoe [Zingiberaceae] 12g, Atractylodes macrocephala Koidz. [Asteraceae] 12g, Pericaeta communisma Gate et Hatai 10g, Codonopsis pilosula (Franch.) Nannf. [Campanulaceae] 10g, Paeonia lactiflora Pall. [Paeoniaceae] 12g, Ziziphus jujuba Mill. [Rhamnaceae] 9g, Glycyrrhiza uralensis Fisch. ex DC. [Fabaceae] 6g	Decoction / Oral administration
Chen 2021	EACP	Astragali Radix 15g, Cinnamomi Ramulus 10g, Spatholobi Caulis 15g, Rehmanniae Radix Recens 10g, Dioscoreae Rhizoma 15g, Paeoniae Radix Alba 10g, Corni Fructus 10g, Euonymi Ramuli Suberalatum 10g, Cnidii Rhizoma 10g, Lumbricus 10g, Corydalis Tuber 15g, Chaenomelis Fructus 15g, Achyranthis Radix 6g	Astragalus mongholicus Bunge [Fabaceae] 15g, Neolitsea cassia (L.) Kosterm. [Lauraceae] 10g, Spatholobus suberectus Dunn [Fabaceae] 15g, Rehmannia glutinosa (Gaertn.) DC. [Orobanchaceae] 10g, Dioscorea polystachya Turcz. [Dioscoreaceae] 15g, Paeonia lactiflora Pall. [Paeoniaceae] 10g, Cornus officinalis Siebold & Zucc. [Cornaceae] 10g, Euonymus alatus (Thunb.) Siebold [Celastraceae] 10g, Conioselinum anthriscoides 'Chuanxiong' [Apiaceae] 10g, Pericaeta communisma	Decoction / Oral administration

			Gate et Hatai 10g, Corydalis yanhusuo (Y.H.Chou & Chun C.Hsu) W.T.Wang ex Z.Y.Su & C.Y.Wu [Papaveraceae] 15g, Pseudocydonia sinensis (Dum.Cours.) C.K.Schneid. [Rosaceae] 15g, Achyranthes bidentata Blume [Amaranthaceae] 6g	
Hou 2021	EACP	Astragali Radix, Cinnamomi Ramulus, Spatholobi Caulis, Puerariae Radix, Curcumae Radix, Lumbricus, Hirudo, Poria Sclerotium, Rehmanniae Radix Recens	Astragalus mongholicus Bunge [Fabaceae], Neolitsea cassia (L.) Kosterm. [Lauraceae], Spatholobus suberectus Dunn [Fabaceae], Pueraria montana var. lobata (Willd.) Maesen & S.M.Almeida ex Sanjappa & Predeep [Fabaceae], Curcuma longa L. [Zingiberaceae], Pericaeta communisma Gate et Hatai, Hirudo niponica Whitman, Poria cocos Wolf, Rehmannia glutinosa (Gaertn.) DC. [Orobanchaceae]	Pill / Oral administration
Li 2021	ECCP	Astragali Radix 30g, Cinnamomi Ramulus 15g, Angelicae Sinensis Radix 15g, Paeoniae Radix Alba 15g, Cnidii Rhizoma 15g, Salviae Miltiorrhizae Radix 15g, Moutan Radicis Cortex 15g, Carthami Flos 5g, Glycyrrhizae Radix et Rhizoma 5g	Astragalus mongholicus Bunge [Fabaceae] 30g, Neolitsea cassia (L.) Kosterm. [Lauraceae] 15g, Angelica sinensis (Oliv.) Diels [Apiaceae] 15g, Paeonia lactiflora Pall. [Paeoniaceae] 15g, Conioselinum anthriscoides 'Chuanxiong' [Apiaceae] 15g, Salvia miltiorrhiza Bunge [Lamiaceae] 15g, Paeonia × suffruticosa Andrews [Paeoniaceae] 15g, Carthamus tinctorius L. [Asteraceae] 5g, Glycyrrhiza uralensis Fisch. ex DC. [Fabaceae] 5g	Decoction / Oral administration
Wang 2021a	ECWP	Astragali Radix 30g, Codonopsis Pilosulae Radix 15g, Angelicae Sinensis Radix 15g, Persicae Semen 15g, Carthami Flos 15g, Rehmanniae Radix Preparata 15g, Cnidii Rhizoma 10g, Paeoniae Radix Rubra 10g, Lumbricus 10g, Paeoniae Radix Alba 10g, Glycyrrhizae Radix et Rhizoma 10g	Astragalus mongholicus Bunge [Fabaceae] 30g, Codonopsis pilosula (Franch.) Nannf. [Campanulaceae] 15g, Angelica sinensis (Oliv.) Diels [Apiaceae] 15g, Prunus persica (L.) Batsch [Rosaceae] 15g, Carthamus tinctorius L. [Asteraceae] 15g, Rehmannia glutinosa (Gaertn.) DC. [Orobanchaceae] 15g, Conioselinum anthriscoides 'Chuanxiong' [Apiaceae] 10g, Paeonia anomala subsp. veitchii (Lynch) D.Y.Hong & K.Y.Pan [Paeoniaceae] 10g, Pericaeta communisma Gate et Hatai 10g, Paeonia lactiflora Pall. [Paeoniaceae]10g, Glycyrrhiza uralensis Fisch. ex DC. [Fabaceae] 10g	Decoction / Oral administration
Wang 2021b	ECWP	Cinnamomi Ramulus 15g, Spatholobi Caulis 20g,	Neolitsea cassia (L.) Kosterm. [Lauraceae] 15g, Spatholobus	Decoction / Oral

		Angelicae Sinensis Radix 20g, Rehmanniae Radix Preparata 15g, Paeoniae Radix Rubra 15g, Cnidii Rhizoma 10g, Persicae Semen 15g, Carthami Flos 10g, Mori Ramulus 15g, Corydalis Tuber 15g, Chaenomelis Fructus 20g, Achyranthis Radix 15g	suberectus Dunn [Fabaceae] 20g, Angelica sinensis (Oliv.) Diels [Apiaceae] 20g, Rehmannia glutinosa (Gaertn.) DC. [Orobanchaceae] 15g, Paeonia anomala subsp. veitchii (Lynch) D.Y.Hong & K.Y.Pan [Paeoniaceae] 15g, Conioselinum anthriscoides 'Chuanxiong' [Apiaceae] 10g, Prunus persica (L.) Batsch [Rosaceae] 15g, Carthamus tinctorius L. [Asteraceae] 10g, Morus alba L. [Moraceae] 15g, Corydalis yanhusuo (Y.H.Chou & Chun C.Hsu) W.T.Wang ex Z.Y.Su & C.Y.Wu [Papaveraceae] 15g, Pseudocydonia sinensis (Dum.Cours.) C.K.Schneid. [Rosaceae] 20g, Achyranthes bidentata Blume [Amaranthaceae] 15g	administration
Zhang 2021	ECCP	Astragali Radix 30g, Cinnamomi Ramulus 12g, Spatholobi Caulis 30g, Paeoniae Radix Alba 15g, Angelicae Sinenesis Radix 15g, Clematidis Radix 15g, Zingiberis Rhizoma Recens 6g, Zizyphi Fructus 9g	Astragalus mongholicus Bunge [Fabaceae] 30g, Neolitsea cassia (L.) Kosterm. [Lauraceae] 12g, Spatholobus suberectus Dunn [Fabaceae] 30g, Angelica sinensis (Oliv.) Diels [Apiaceae] 15g, Clematis terniflora var. mandshurica (Rupr.) Ohwi [Ranunculaceae] 15g, Zingiber officinale Roscoe [Zingiberaceae] 6g, Ziziphus jujuba Mill. [Rhamnaceae] 9g	Decoction / Oral administration

EACP: East Asian herbal medicine monotherapy containing the Astragali Radix-Cinnamomi Ramulus herb-pair; EAHM: East Asian herbal medicine; EAWP: East Asian herbal medicine monotherap y without the Astragali Radix-Cinnamomi Ramulus herb-pair; ECCP: East Asian herbal medicine and conventional medicine combined therapy containing the Astragali Radix-Cinnamomi Ramulus h erb-pair; ECWP: East Asian herbal medicine and conventional medicine combined therapy without the Astragali Radix-Cinnamomi Ramulus herb-pair; The Latin names of the herbal constituents we re prepared based on the Korean Pharmacopeia (KP) and the Korean Herbal Pharmacopeia (KHP), and the accepted scientific names of the herbal constituents were prepared based on the Royal B otanic Gardens Kew Science database (https://www.kew.org/science/our-science/science-services/medicinal-plant-names-services)
