# Supplementary material for: Comparative Efficacy of East Asian Herbal Formulae Containing Astragali Radix–Cinnamomi Ramulus Herb-Pair against Diabetic Peripheral Neuropathy and Mechanism Prediction: A Bayesian Network Meta-Analysis Integrated with Network Pharmacology
Source: Pharmaceutics. 2023 Apr 28;15(5):1361. doi: 10.3390/pharmaceutics15051361 (PMC10221388; doi:10.3390/pharmaceutics15051361)
Supplement: Supplementary file 1 [file pharmaceutics-15-01361-s001.zip › Supplementary Table S3.pdf]

Supplementary Table S3. Quality of evidence ratings for secondary outcomes in pairwise meta-analysis

Intervention and comparator intervention	Outcomes	Number of participants (Studies)	Anticipated absolute effects (95% CI)	Quality of the evidence (GRADE)
EACP compared to CM for diabetic peripheral neuropathy	MMNCV	NA	NA	NA
	PMNCV	188(2)	MD 3.27 higher (2.01 higher to 4.54 higher)	⊕⊕⊕◯ MODERATE[a]
	TMNCV	NA	NA	NA
	UMNCV	80(1)	MD 4.55 higher (2.87 higher to 6.22 higher)	⊕⊕◯◯ LOW[a,c]
	MSNCV	151(2)	MD 4.12 higher (3.13 higher to 5.10 higher)	⊕⊕⊕◯ MODERATE[a]
	PSNCV	231(3)	MD 2.89 higher (1.80 higher to 3.98 higher)	⊕⊕⊕◯ MODERATE[a]
	TSNCV	84(1)	MD 2.10 higher (0.94 higher to 3.26 higher)	⊕⊕◯◯ LOW[a,c]
	USNCV	147(2)	MD 3.45 higher (1.52 higher to 5.39 higher)	⊕⊕⊕◯ MODERATE[a]
ECCP compared to CM for diabetic peripheral neuropathy	MMNCV	423(4)	MD 5.01 higher (3.47 higher to 6.56 higher)	⊕⊕◯◯ LOW[a,c]
	PMNCV	379(4)	MD 3.40 higher (2.04 higher to 4.75 higher)	⊕⊕◯◯ LOW[a,c]
	TMNCV	216(2)	MD 2.98 higher (1.92 higher to 4.05 higher)	⊕⊕⊕◯ MODERATE[a]
	UMNCV	120(1)	MD 3.30 higher (2.28 higher to 4.32 higher)	⊕⊕◯◯ LOW[a,c]
	MSNCV	437(4)	MD 4.93 higher (4.14 higher to 5.72 higher)	⊕⊕⊕◯ MODERATE[a]
	PSNCV	511(5)	MD 3.51 higher (2.07 higher to 4.96 higher)	⊕⊕◯◯ LOW[a,c]
	TSNCV	328(3)	MD 4.51 higher (3.36 higher to 5.65 higher)	⊕⊕◯◯ LOW[a,c]
	USNCV	216(2)	MD 5.05 higher (4.23 higher to 5.88 higher)	⊕⊕⊕◯ MODERATE[a]

EAWP compared to CM for diabetic peripheral neuropathy	MMNCV	422(4)	MD 1.64 higher (0.72 higher to 2.57 higher)	⊕⊕⊕◯ MODERATE[a]
	PMNCV	782(6)	MD 2.20 higher (1.18 higher to 3.22 higher)	⊕⊕◯◯ LOW[a,c]
	TMNCV	280(3)	MD 3.94 higher (0.01 lower to 7.8982 higher)	⊕◯◯◯ VERY LOW[a,b,c]
	UMNCV	160(2)	MD 2.52 higher (0.61 higher to 4.43 higher)	⊕⊕◯◯ LOW[a,c]
	MSNCV	722(7)	MD 2.42 higher (1.20 higher to 3.63 higher)	⊕⊕◯◯ LOW[a,c]
	PSNCV	659(7)	MD 3.30 higher (2.07 higher to 4.54 higher)	⊕⊕◯◯ LOW[a,c]
	TSNCV	464(4)	MD 3.16 higher (2.55 higher to 3.77 higher)	⊕⊕⊕◯ MODERATE[a]
	USNCV	160(2)	MD 1.94 higher (0.03 higher to 3.84 higher)	⊕⊕◯◯ LOW[a,c]
ECWP compared to CM for diabetic peripheral neuropathy	MMNCV	520(6)	MD 2.66 higher (1.38 higher to 3.93 higher)	⊕⊕◯◯ LOW[a,c]
	PMNCV	743(9)	MD 3.20 higher (2.22 higher to 4.19 higher)	⊕⊕◯◯ LOW[a,c]
	TMNCV	370(4)	MD 3.79 higher (1.82 higher to 5.77 higher)	⊕⊕◯◯ LOW[a,c]
	UMNCV	NA	NA	NA
	MSNCV	584(7)	MD 2.22 higher (1.20 higher to 3.24 higher)	⊕⊕◯◯ LOW[a,c]
	PSNCV	656(8)	MD 2.05 higher (1.05 higher to 3.04 higher)	⊕⊕◯◯ LOW[a,c]
	TSNCV	472(5)	MD 3.16 higher (2.07 higher to 4.25 higher)	⊕⊕◯◯ LOW[a,c]
	USNCV	NA	NA	NA

CM: conventional medicine ; EACP: east Asian herbal medicine monotherapy contained astragali radix-cinnamomi ramulus herb pair ; EAHM; east Asian herbal medicine ; EAWP: east Asian herbal medicine monotherapy without astragali radix-cinnamomi ramulus herb pair ; ECCP: east Asian herbal medicine and conventional medicine combined therapy contained astragali radix-cinnamomi ramulus herb pair ; ECWP: east Asian herbal medicine and conventional medicine combined therapy without astragali radix-cinnamomi ramulus herb pair; MD, mean difference; MMNCV, median motor nerve conduction velocity; MSNCV, median sensory nerve conduction velocity; PMNCV, peroneal motor nerve conduction velocity ; PSNCV, peroneal sensory nerve conduction velocity; TMNCV, tibial motor nerve conduction velocity; TSNCV NA, not applicable; RR, risk ratio; RCT, randomized clinical trial; SD, standardized difference; SMD, standardized mean difference

GRADE working group grades of evidence.

High quality: Further research is unlikely to change our confidence in estimating the effect.

Moderate quality: Further research is likely to have an important impact on our confidence in estimating the effect and may change the estimate.

Low quality: Further research is very likely to impact our confidence in the estimate of the effect and is likely to change the estimate.

Very low quality: Very uncertain about the estimate.

a: Study design with some bias in randomized or distributed blind.

b: The 95% confidence interval passes 0 (MD and SMD) or 1 (RR and OR), and other interventions were not satisfied.

c: The confidence intervals are less overlapping, or the heterogeneity is high.
