# Supplementary figures and images for: Comparative Efficacy of East Asian Herbal Formulae Containing Astragali Radix–Cinnamomi Ramulus Herb-Pair against Diabetic Peripheral Neuropathy and Mechanism Prediction: A Bayesian Network Meta-Analysis Integrated with Network Pharmacology
Source: Pharmaceutics. 2023 Apr 28;15(5):1361. doi: 10.3390/pharmaceutics15051361 (PMC10221388; doi:10.3390/pharmaceutics15051361)

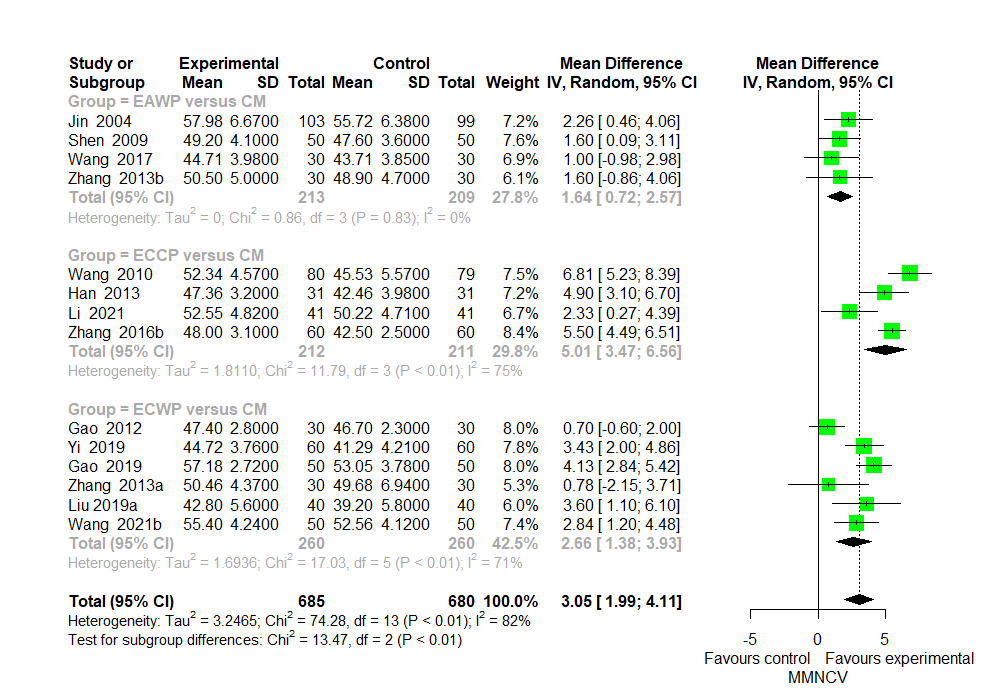

Supplement: Supplementary file 1 [file pharmaceutics-15-01361-s001.zip › Supplementary Figure S1.png]

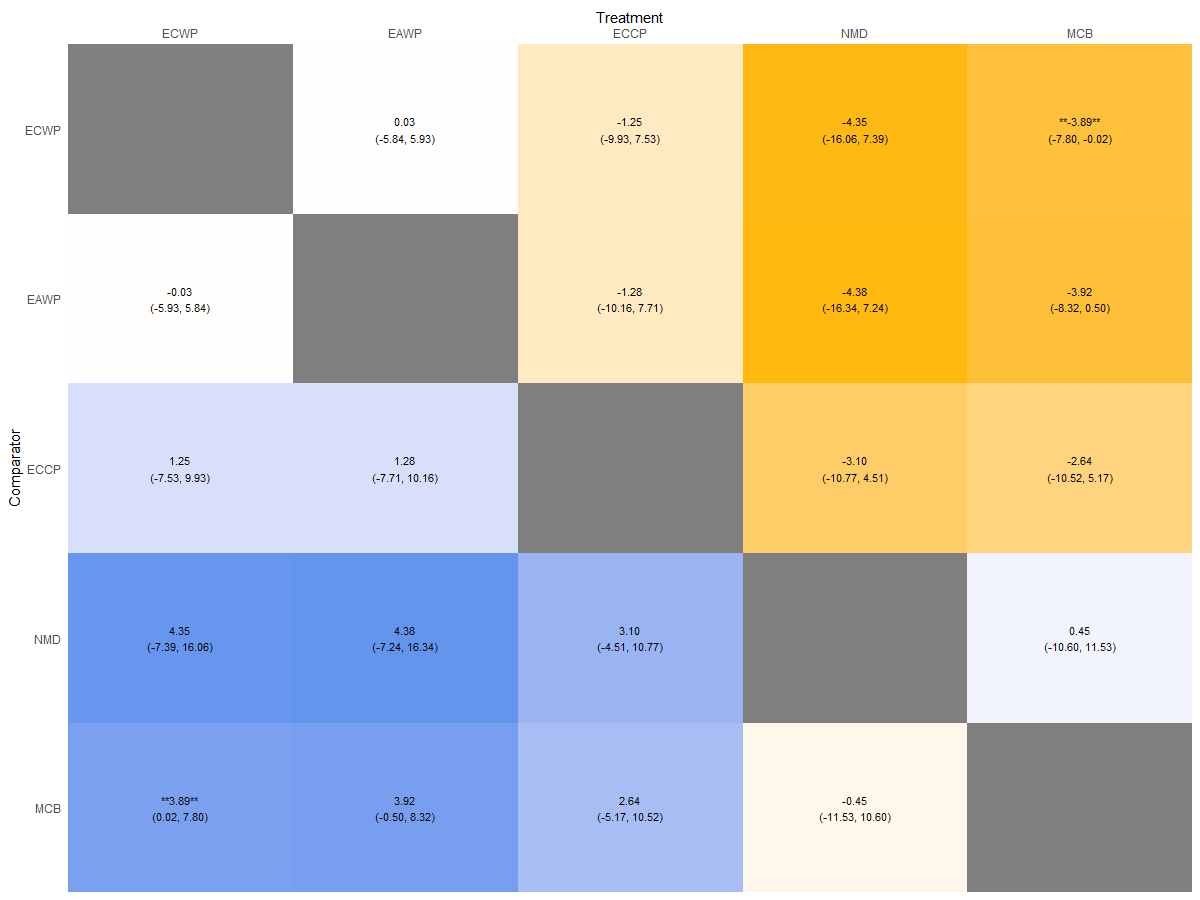

Supplement: Supplementary file 1 [file pharmaceutics-15-01361-s001.zip › Supplementary Figure S10 League heat plot for TMNCV.png]

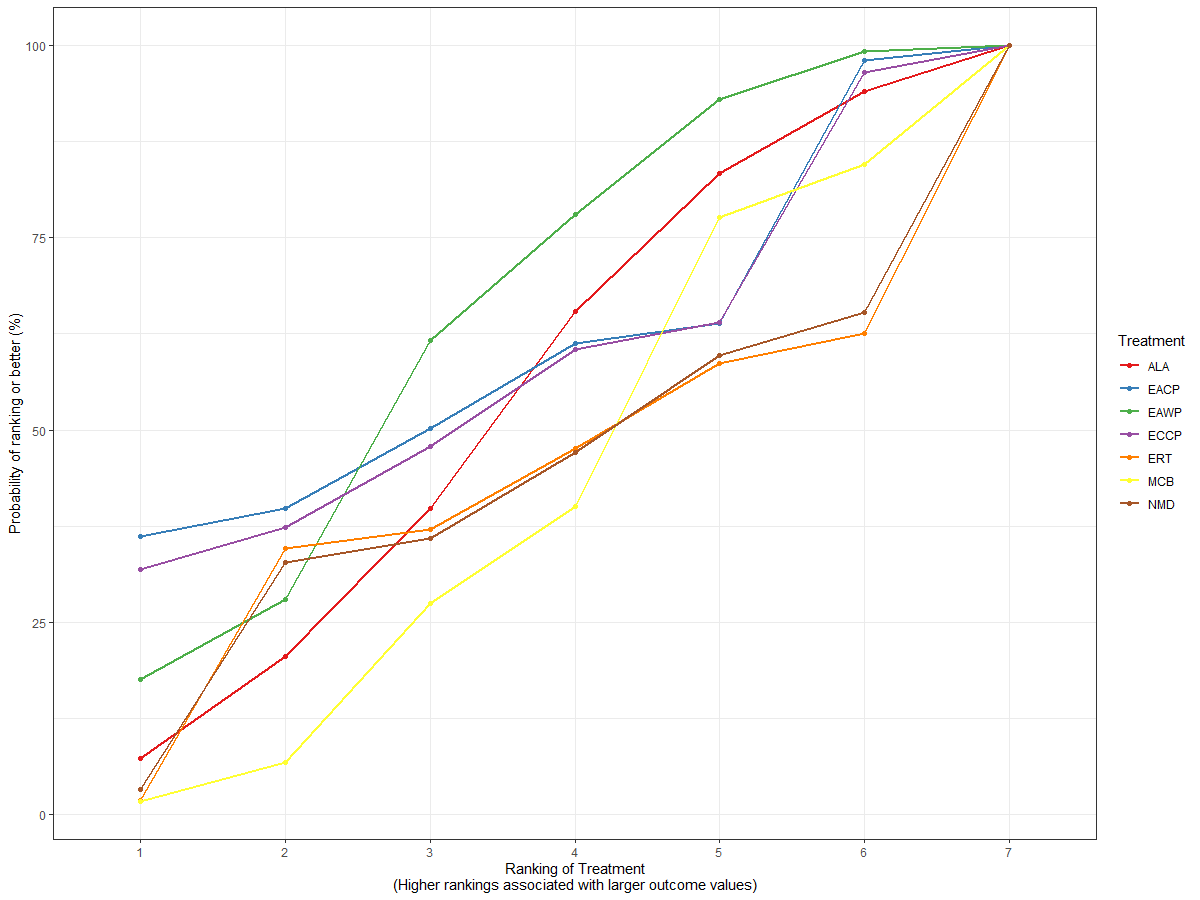

Supplement: Supplementary file 1 [file pharmaceutics-15-01361-s001.zip › Supplementary Figure S11 SUCRA plot for UMNCV.png]

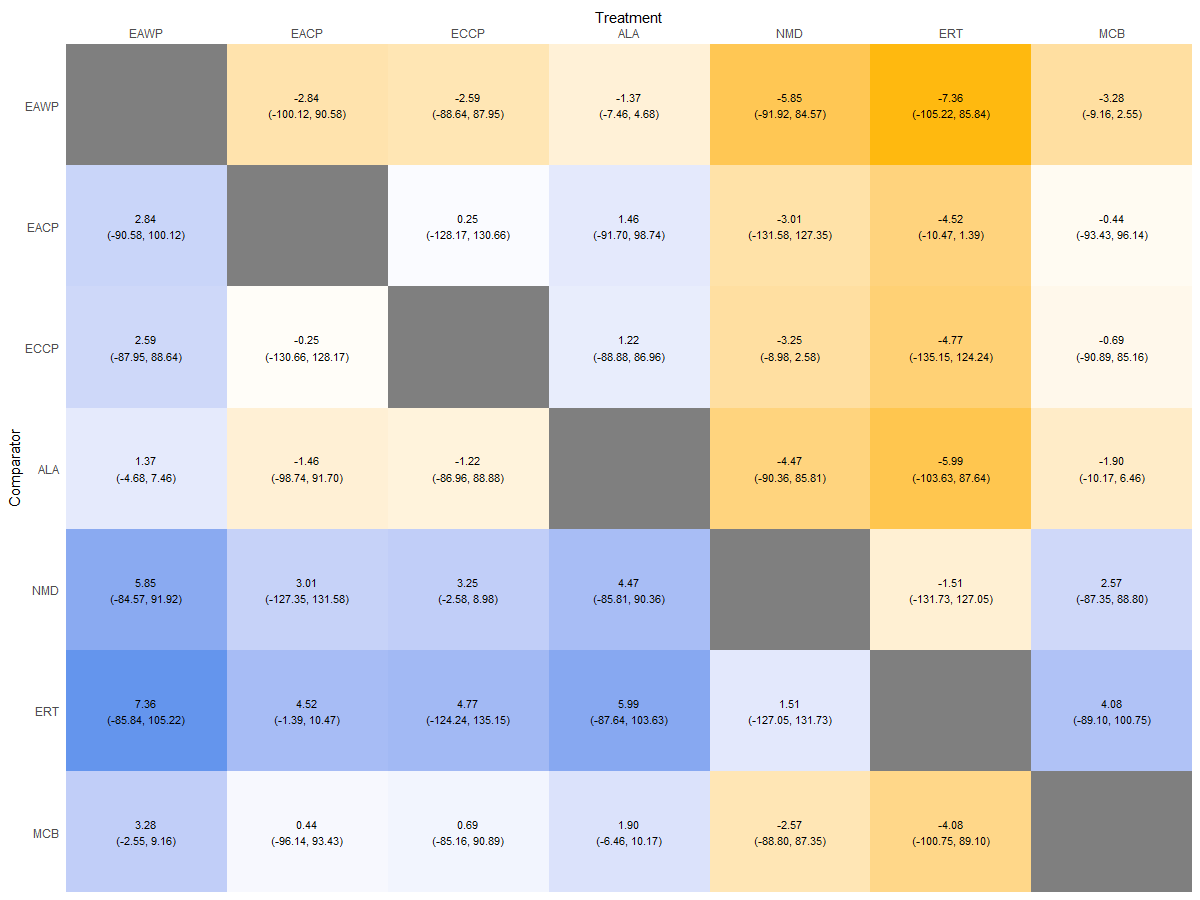

Supplement: Supplementary file 1 [file pharmaceutics-15-01361-s001.zip › Supplementary Figure S12 League heat plot for UMNCV.png]

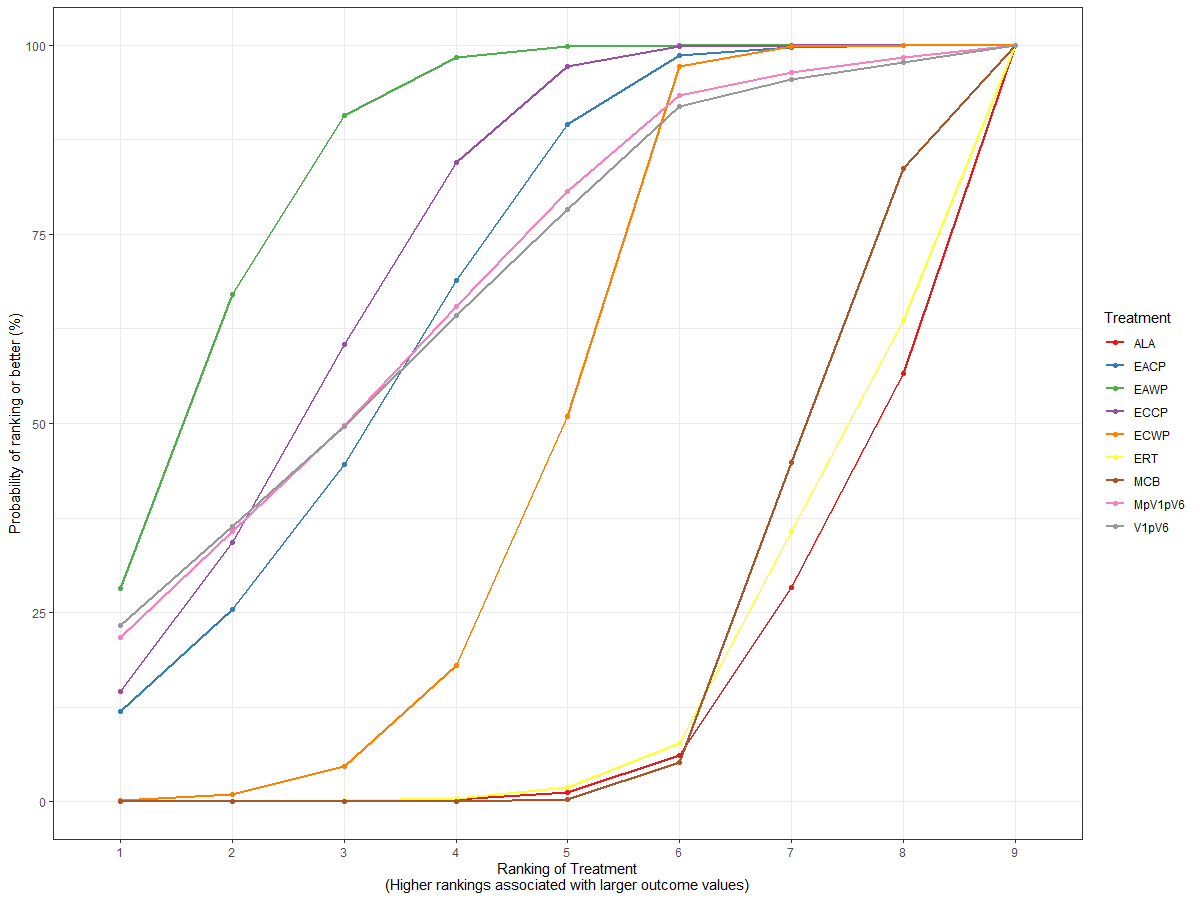

Supplement: Supplementary file 1 [file pharmaceutics-15-01361-s001.zip › Supplementary Figure S13 SUCRA plot for PSNCV.png]

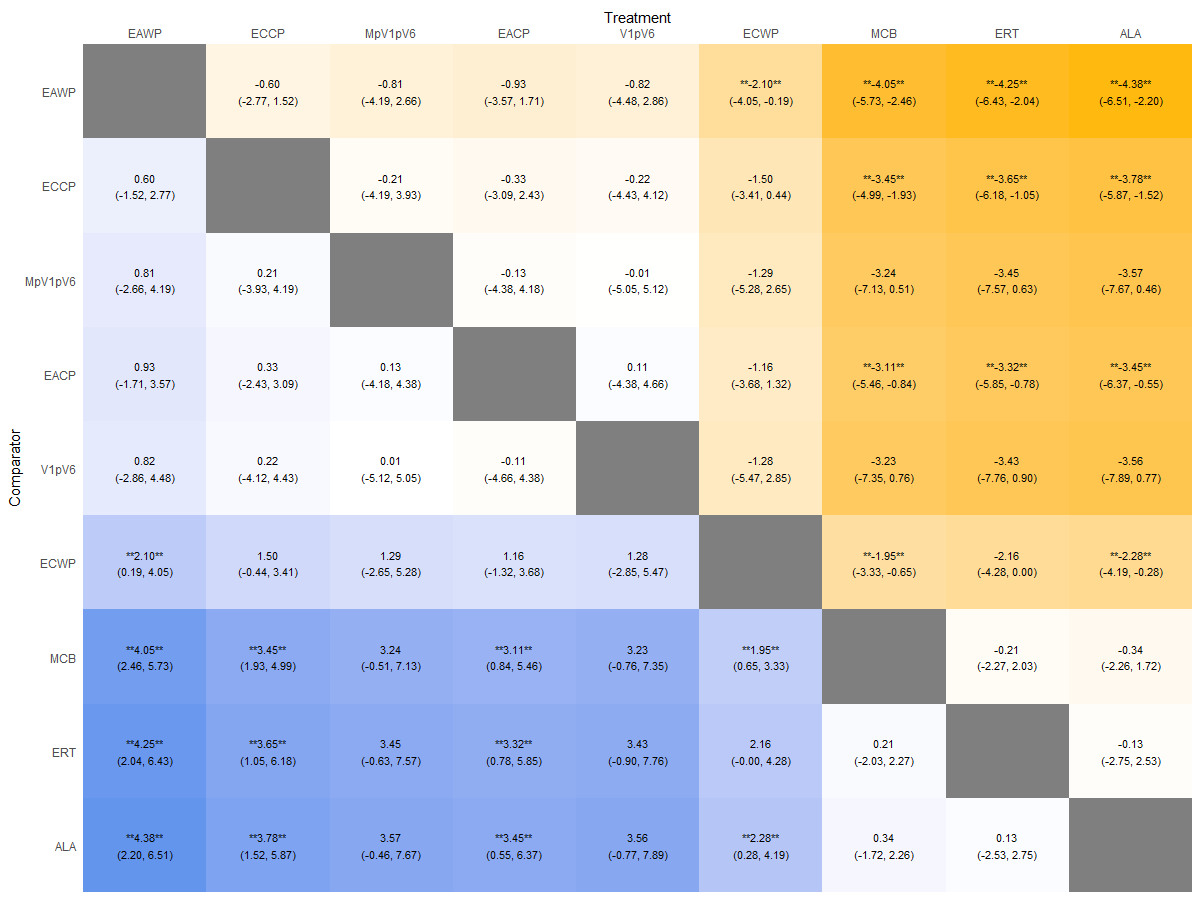

Supplement: Supplementary file 1 [file pharmaceutics-15-01361-s001.zip › Supplementary Figure S14 League heat plot for PSNCV.png]

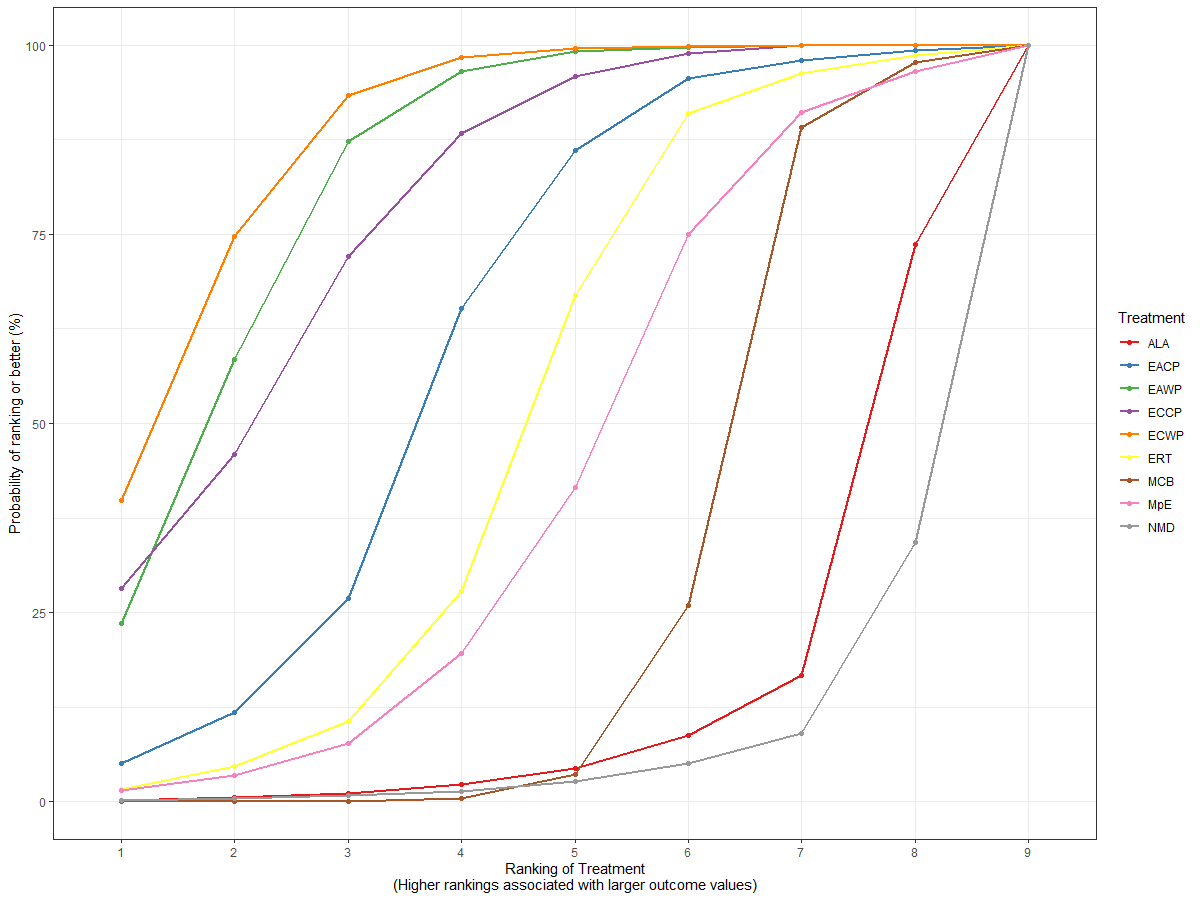

Supplement: Supplementary file 1 [file pharmaceutics-15-01361-s001.zip › Supplementary FIgure S15 SUCRA plot for TSNCV.png]

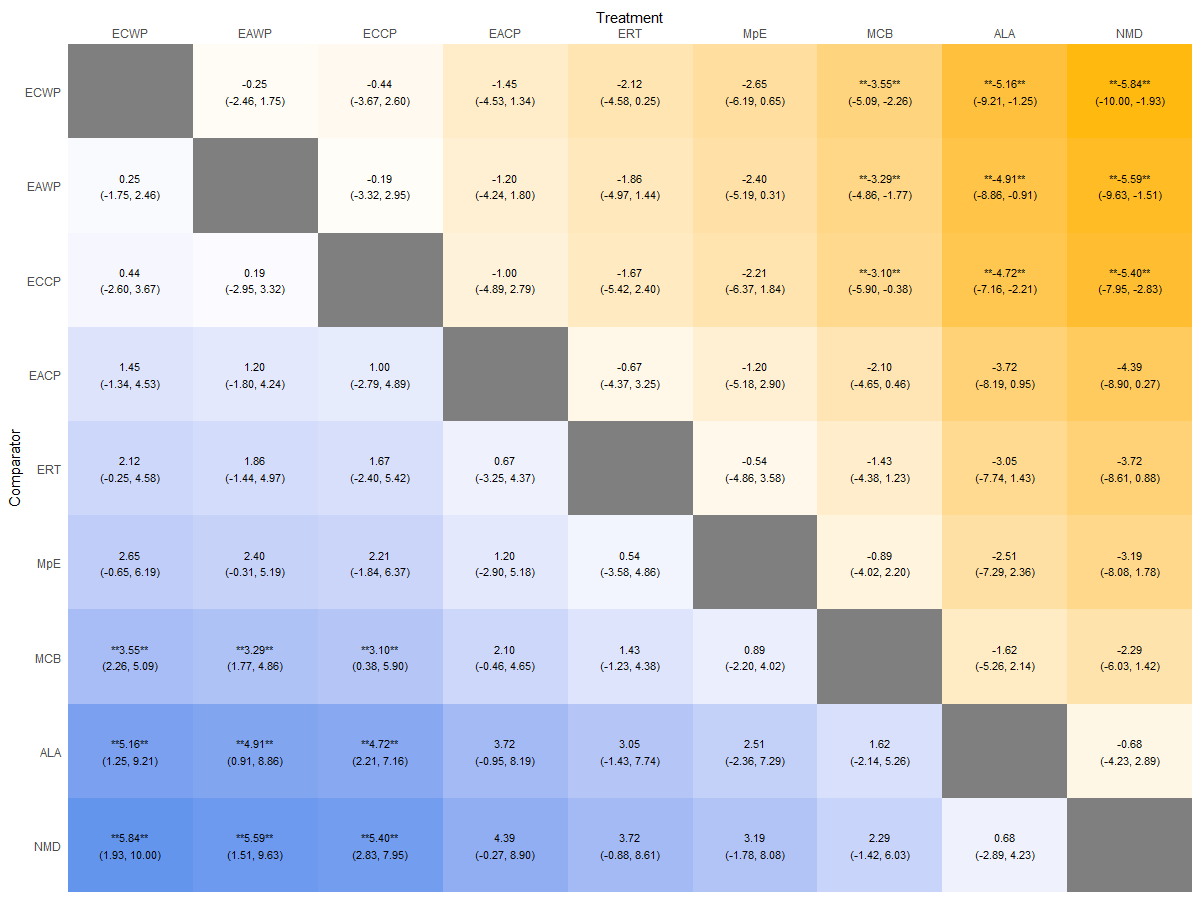

Supplement: Supplementary file 1 [file pharmaceutics-15-01361-s001.zip › Supplementary FIgure S16 League heat plot for TSNCV.png]

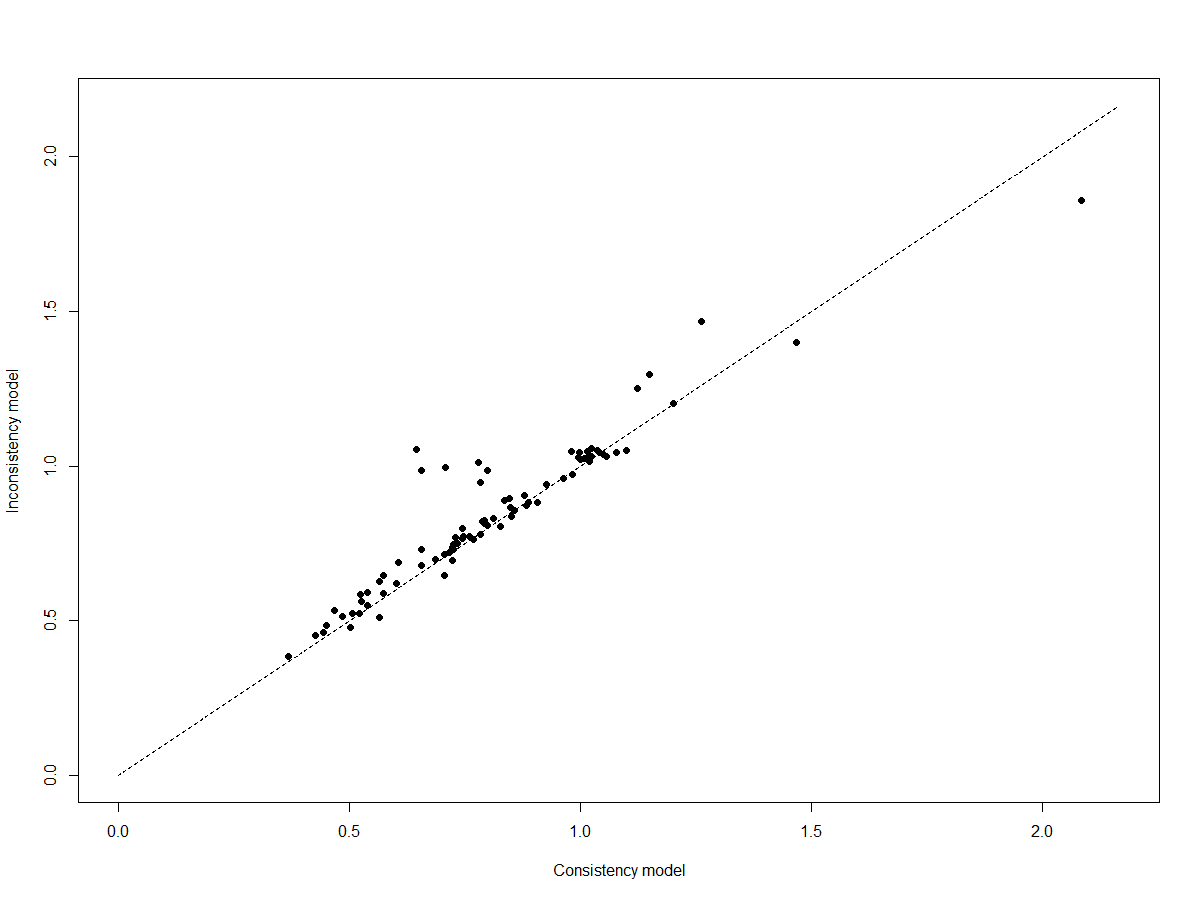

Supplement: Supplementary file 1 [file pharmaceutics-15-01361-s001.zip › Supplementary Figure S17.png]

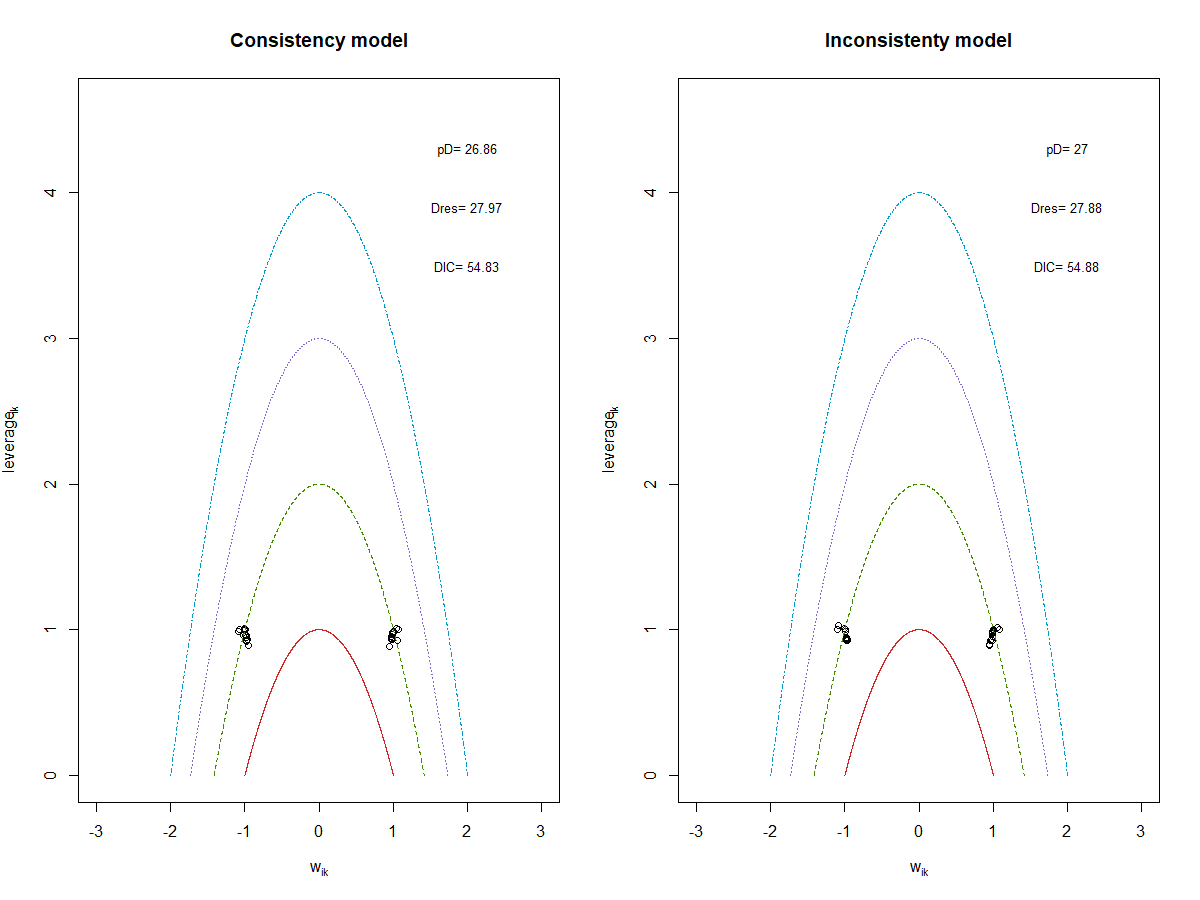

Supplement: Supplementary file 1 [file pharmaceutics-15-01361-s001.zip › Supplementary Figure S18 (A).png]

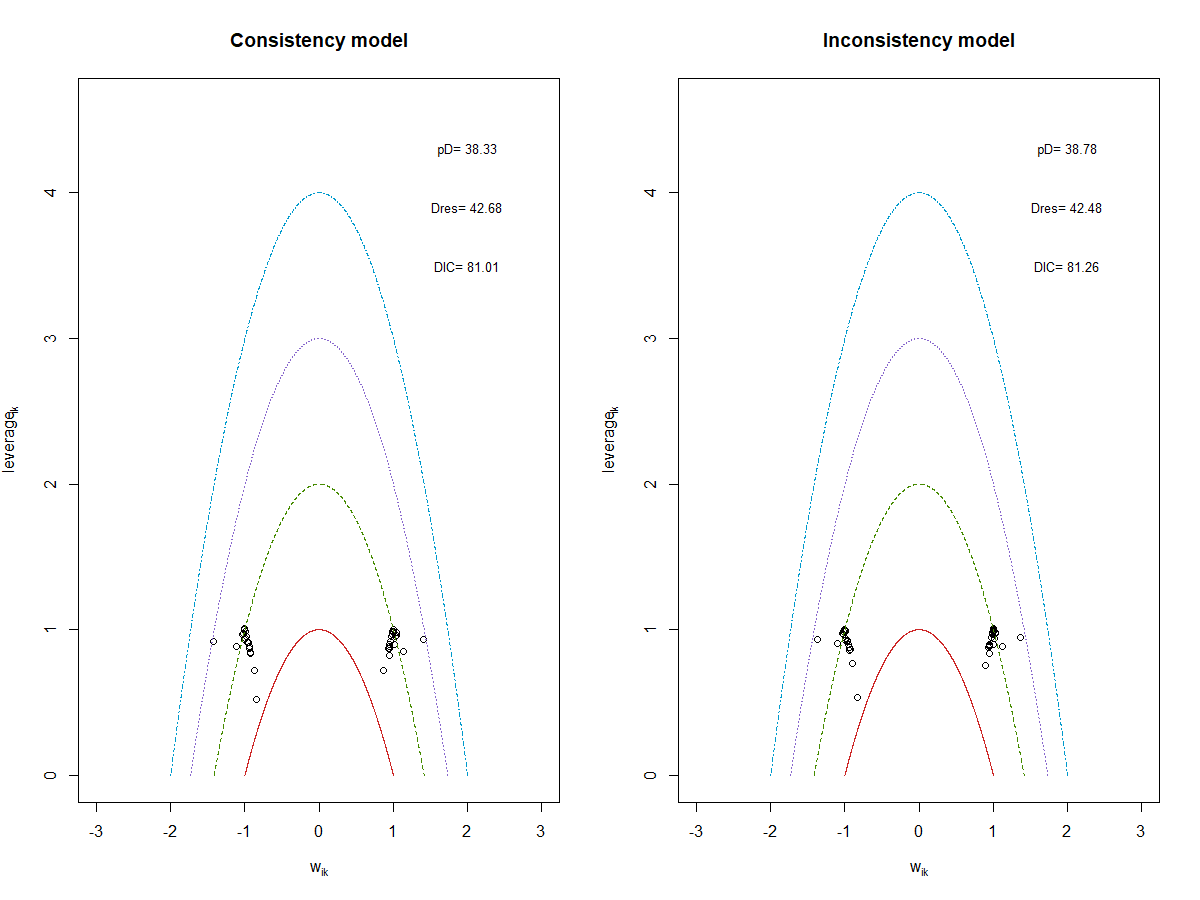

Supplement: Supplementary file 1 [file pharmaceutics-15-01361-s001.zip › Supplementary Figure S18 (B).png]

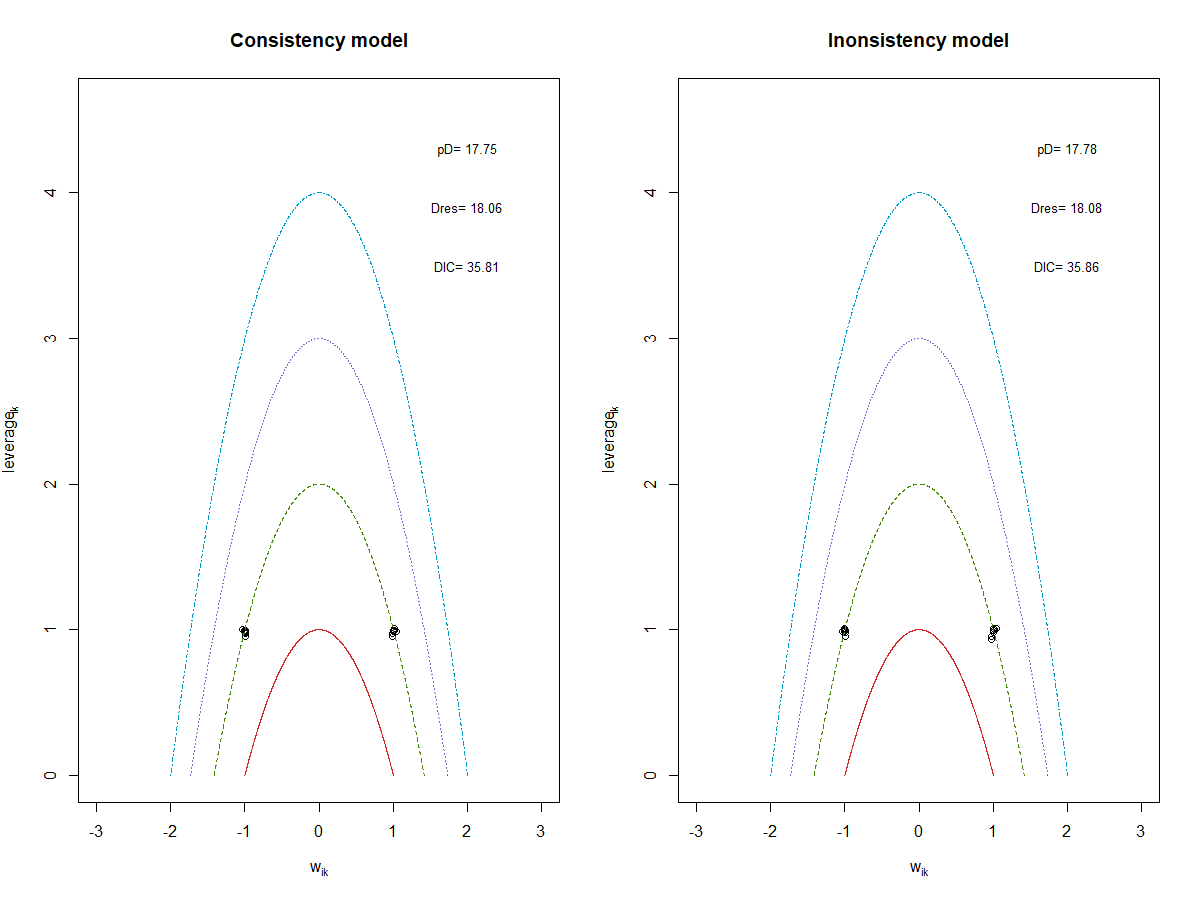

Supplement: Supplementary file 1 [file pharmaceutics-15-01361-s001.zip › Supplementary Figure S18 (C).png]

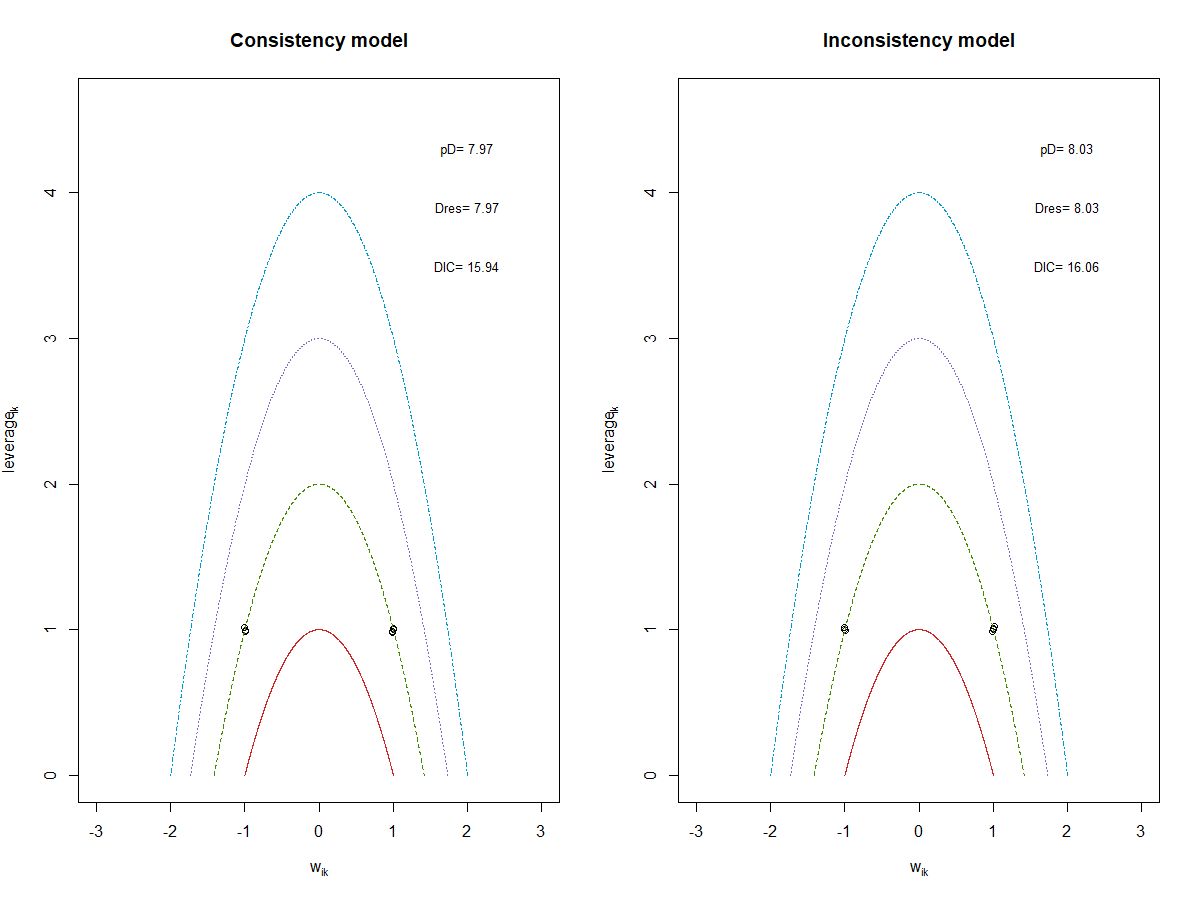

Supplement: Supplementary file 1 [file pharmaceutics-15-01361-s001.zip › Supplementary Figure S18 (D).png]

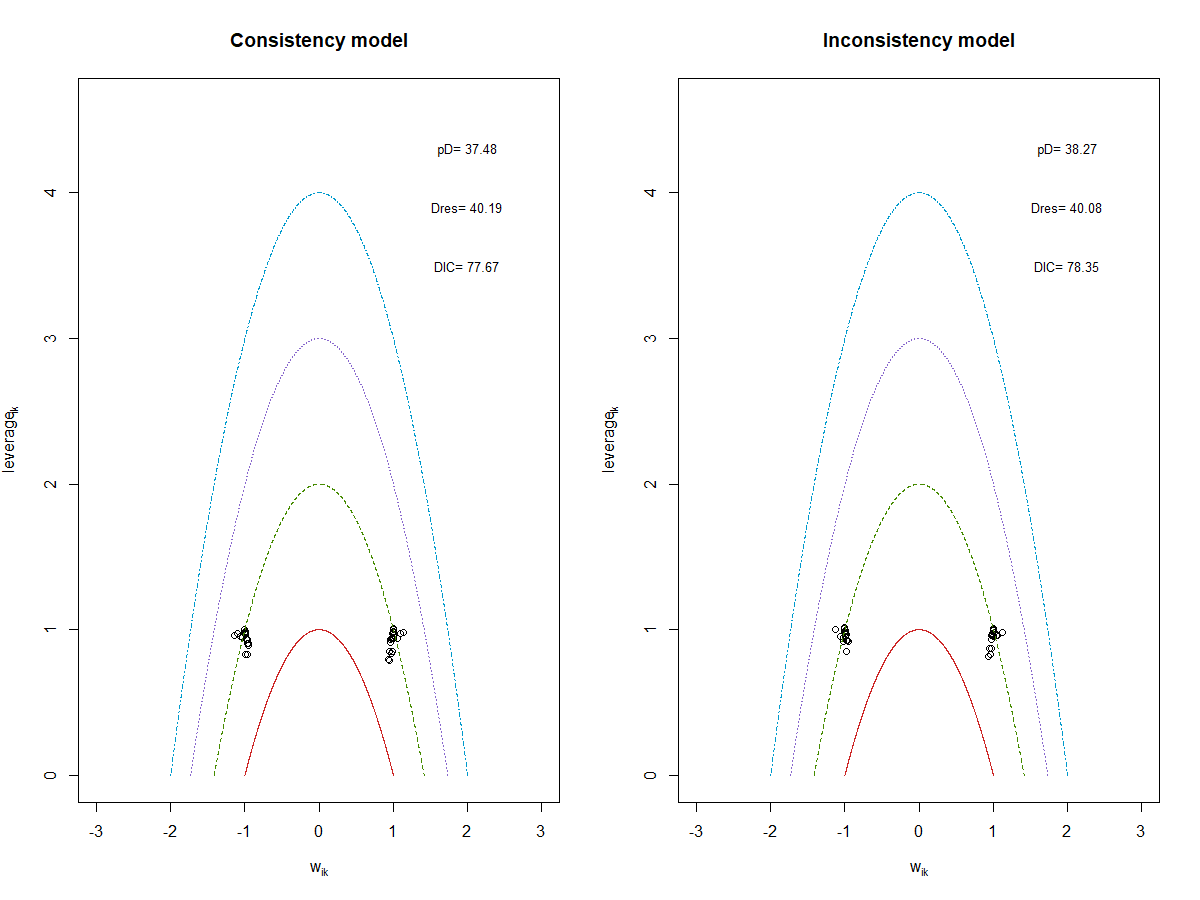

Supplement: Supplementary file 1 [file pharmaceutics-15-01361-s001.zip › Supplementary Figure S18 (E).png]

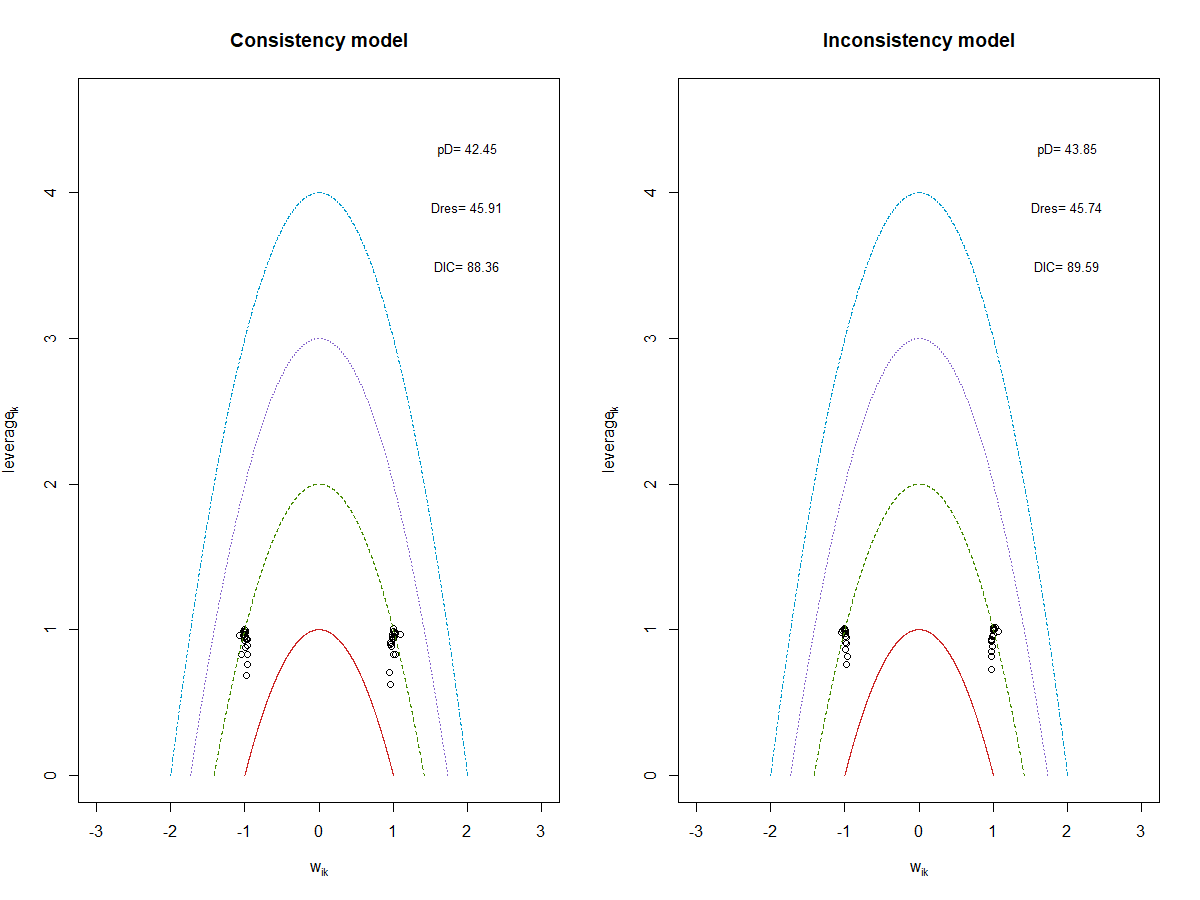

Supplement: Supplementary file 1 [file pharmaceutics-15-01361-s001.zip › Supplementary Figure S18 (F).png]

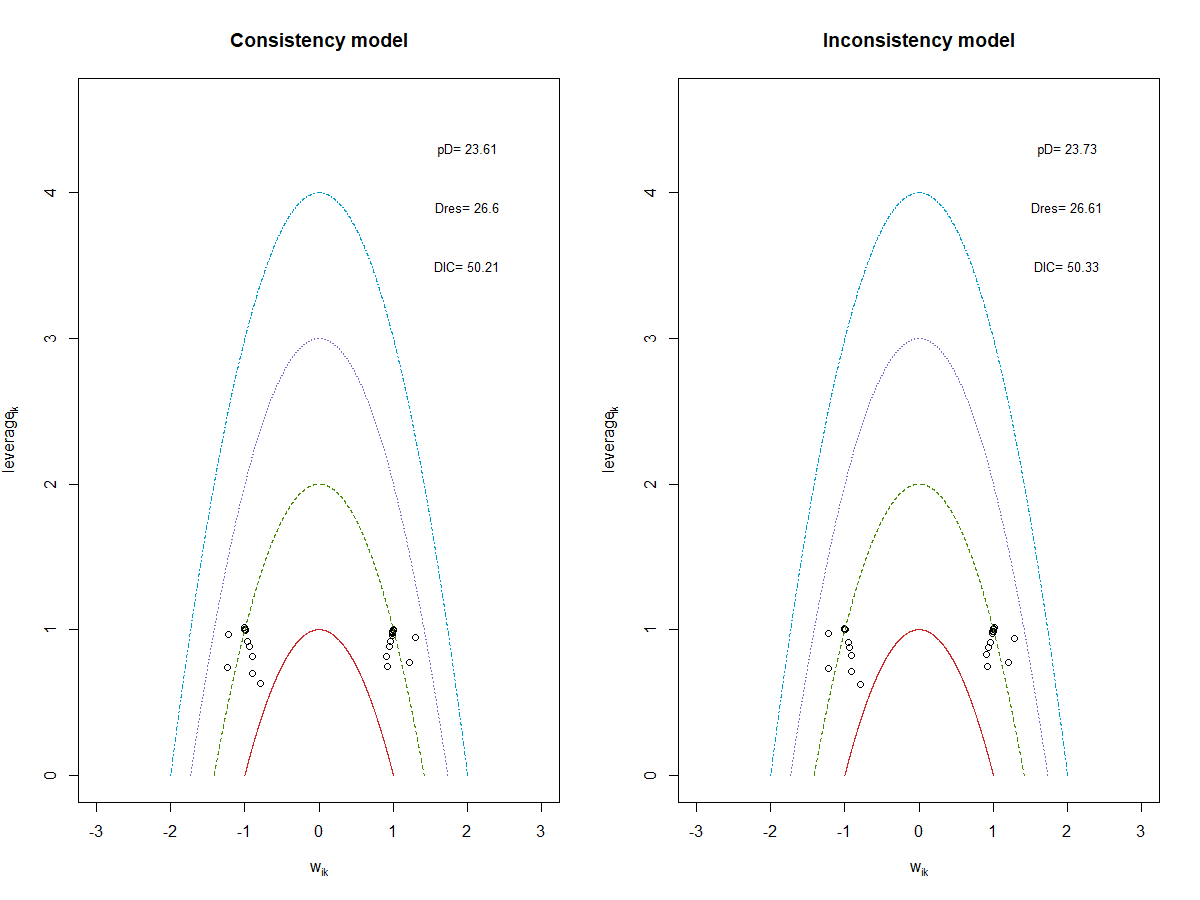

Supplement: Supplementary file 1 [file pharmaceutics-15-01361-s001.zip › Supplementary Figure S18 (G).png]

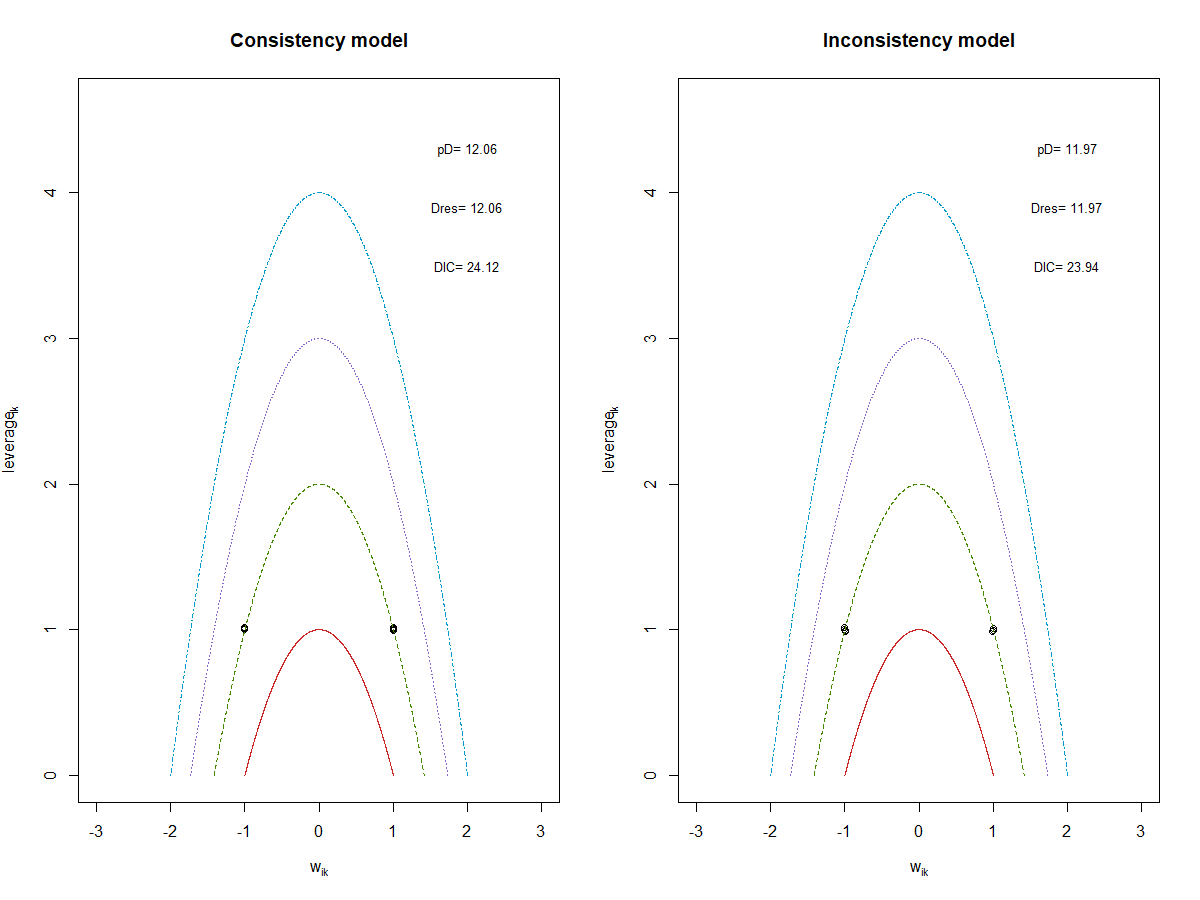

Supplement: Supplementary file 1 [file pharmaceutics-15-01361-s001.zip › Supplementary Figure S18 (H).png]

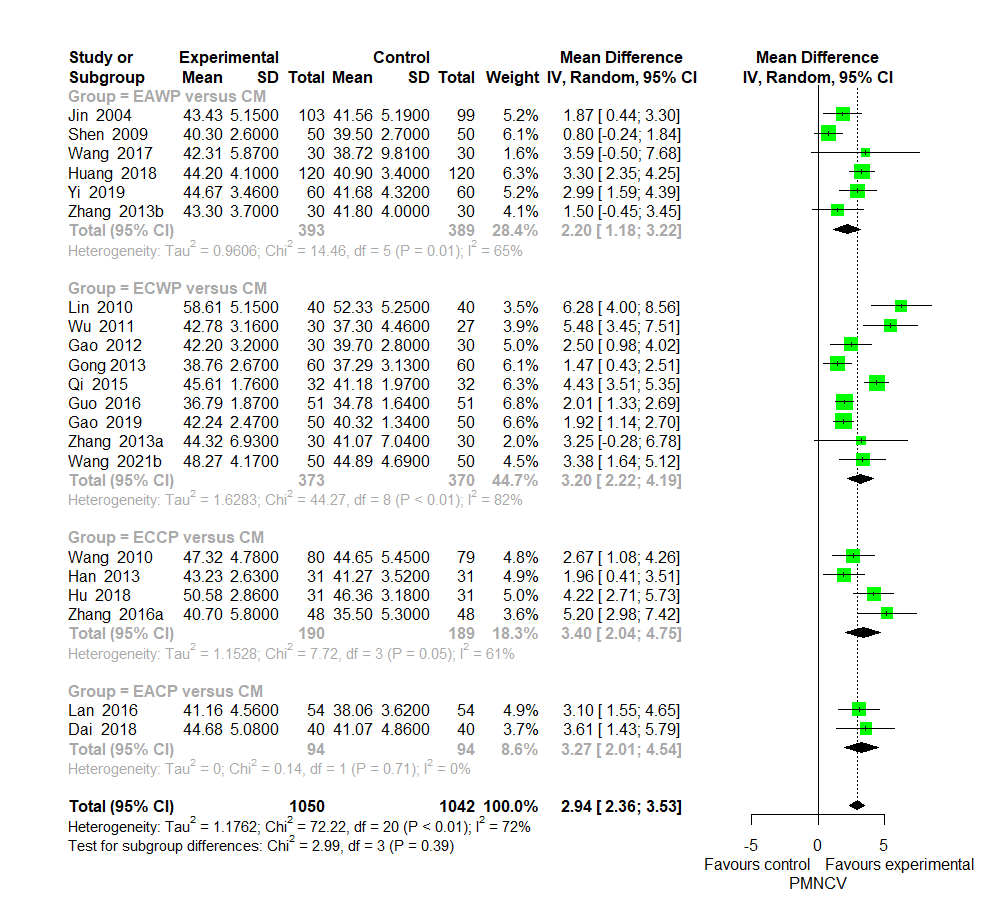

Supplement: Supplementary file 1 [file pharmaceutics-15-01361-s001.zip › Supplementary Figure S2.png]

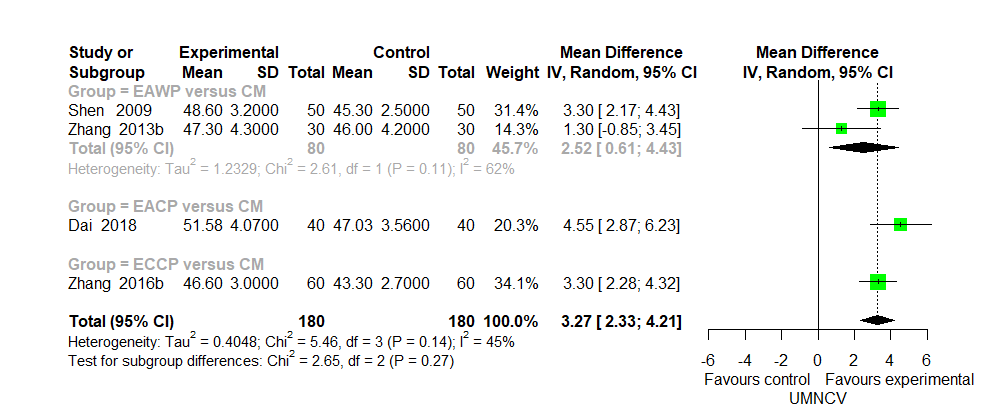

Supplement: Supplementary file 1 [file pharmaceutics-15-01361-s001.zip › Supplementary Figure S3.png]

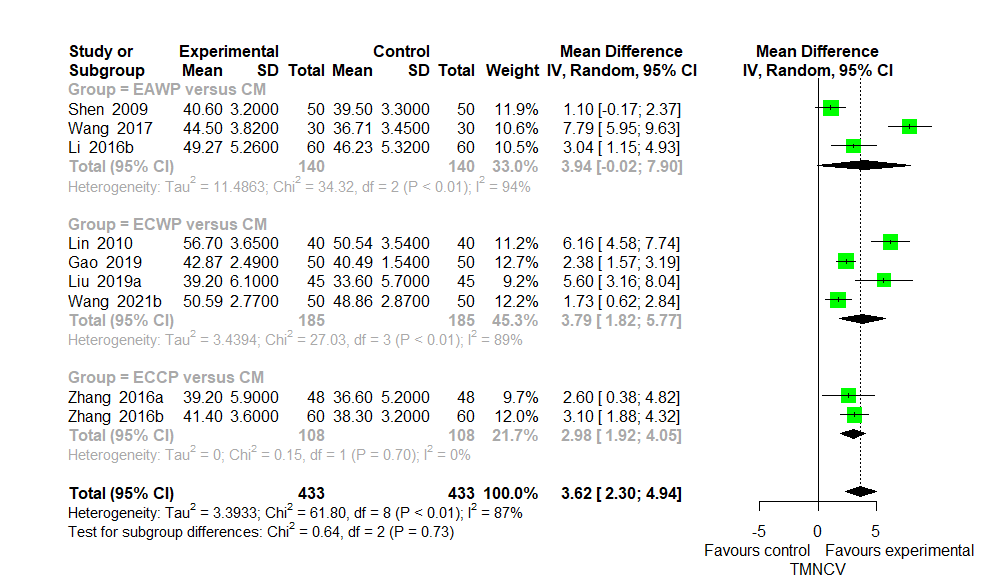

Supplement: Supplementary file 1 [file pharmaceutics-15-01361-s001.zip › Supplementary Figure S4.png]

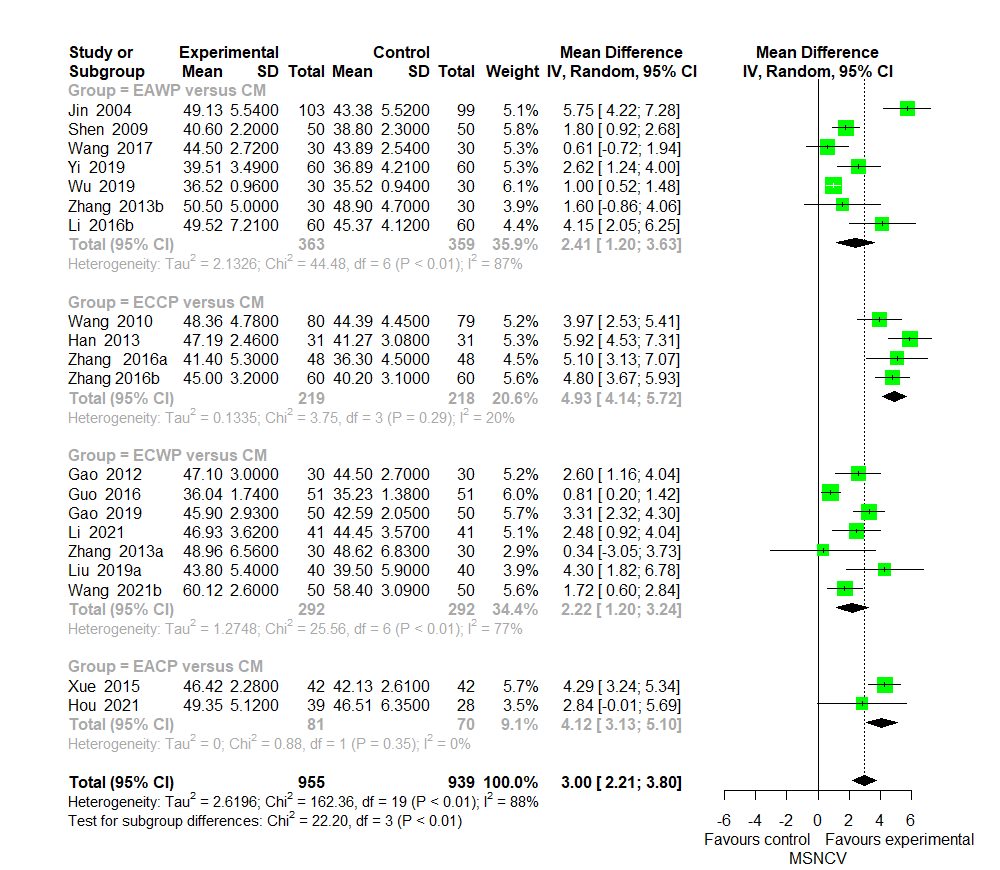

Supplement: Supplementary file 1 [file pharmaceutics-15-01361-s001.zip › Supplementary Figure S5.png]

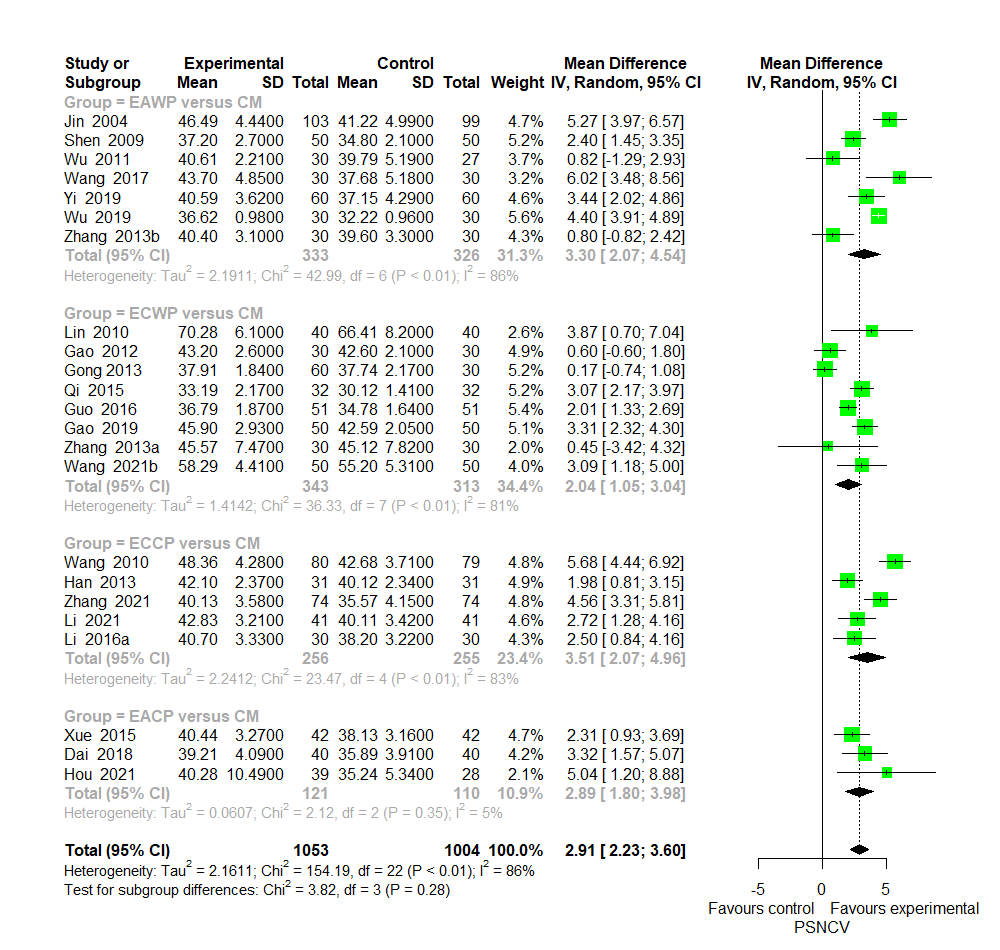

Supplement: Supplementary file 1 [file pharmaceutics-15-01361-s001.zip › Supplementary Figure S6.png]

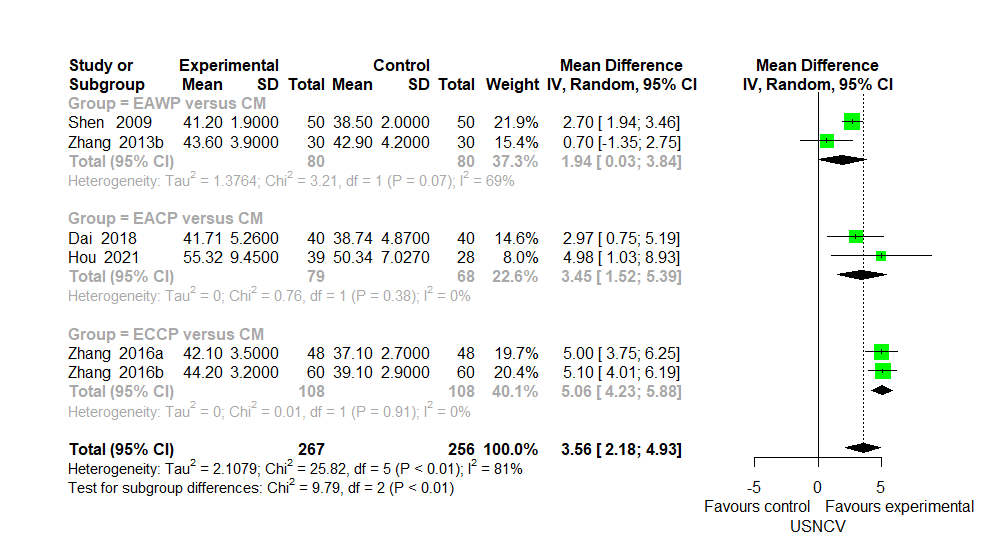

Supplement: Supplementary file 1 [file pharmaceutics-15-01361-s001.zip › Supplementary Figure S7.png]

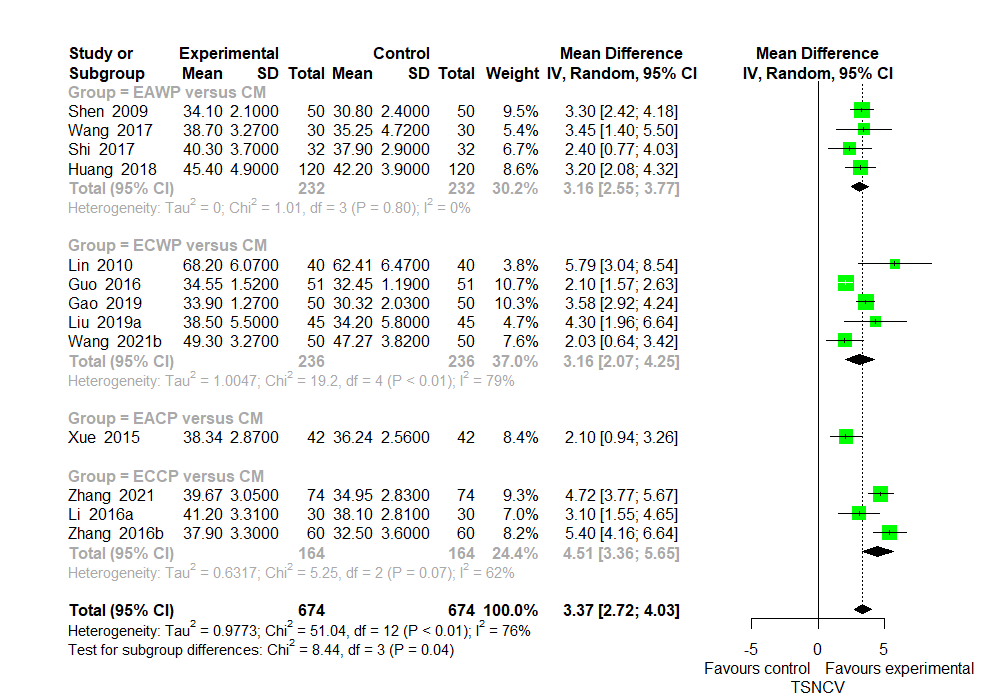

Supplement: Supplementary file 1 [file pharmaceutics-15-01361-s001.zip › Supplementary Figure S8.png]

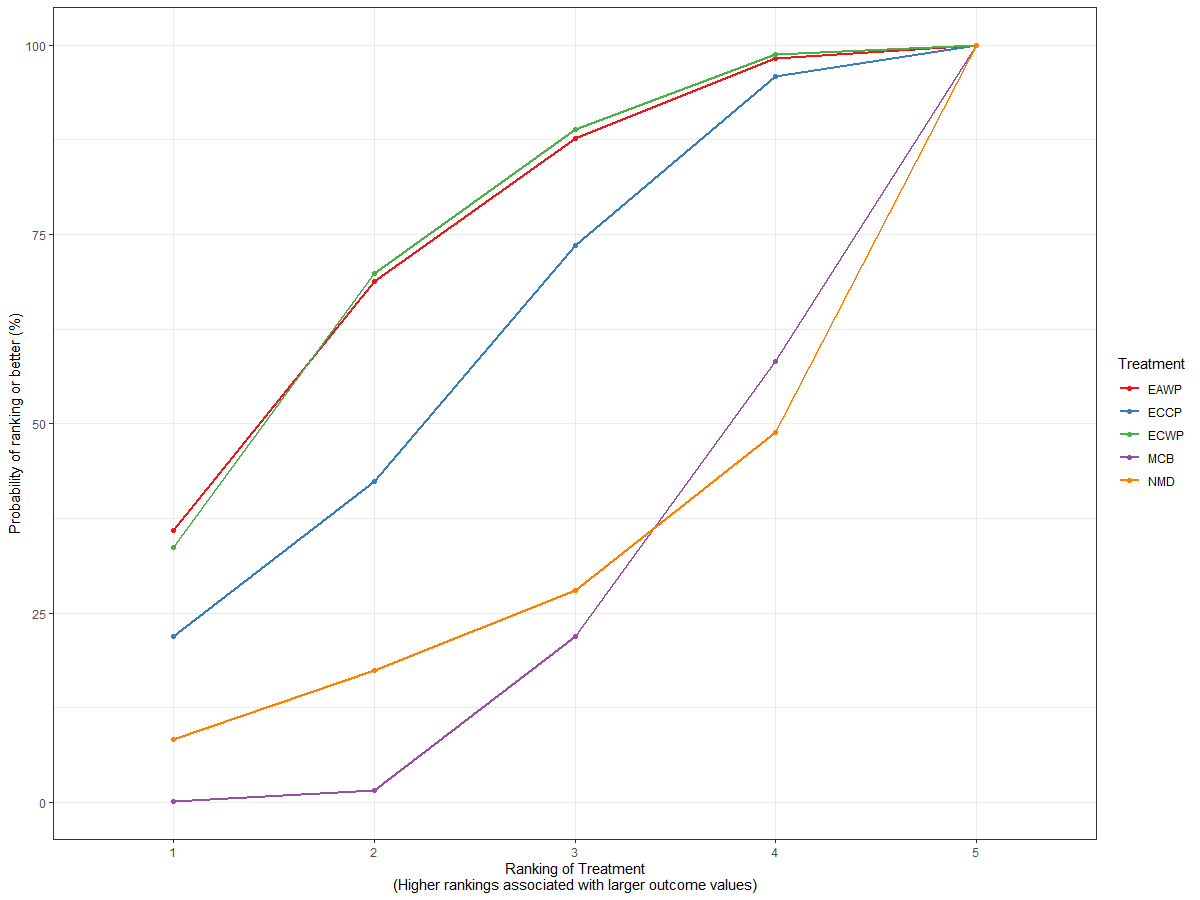

Supplement: Supplementary file 1 [file pharmaceutics-15-01361-s001.zip › Supplementary Figure S9 SUCRA plot for TMNCV.png]
